# Forecasting disability-adjusted life years for chronic diseases: reference and alternative scenarios of salt intake for 2017–2040 in Japan

**DOI:** 10.1186/s12889-020-09596-3

**Published:** 2020-09-29

**Authors:** Shuhei Nomura, Daisuke Yoneoka, Shiori Tanaka, Aya Ishizuka, Peter Ueda, Keiji Nakamura, Hisayuki Uneyama, Naoki Hayashi, Kenji Shibuya

**Affiliations:** 1 Department of Global Health Policy, Graduate School of Medicine, The University of Tokyo, Tokyo, Japan; 2 Department of Health Policy and Management, School of Medicine, Keio University, 35 Shinanomachi, Shinjuku-ku, Tokyo, 160-8582 Japan; 3 Epidemiology and Prevention Group, Center for Public Health Sciences, National Cancer Center, Tokyo, Japan; 4 Graduate School of Public Health, St. Luke’s International University, Tokyo, Japan; 5 Clinical Epidemiology Division, Department of Medicine, Karolinska Institutet, Solna, Stockholm, Sweden; 6 Graduate School of Environmental and Information Studies, Tokyo City University, Yokohama, Japan; 7 Ajinomoto Co., Inc., Tokyo, Japan; 8 Department of Applied Biological Chemistry, Graduate School of Agriculture and Life Sciences, The University of Tokyo, Tokyo, Japan; 9 Institute for Population Health, King’s College London, London, UK

**Keywords:** Disability-adjusted life years, Salt intake, Japan, Future prediction, Cardiovascular diseases, Chronic kidney diseases, Stomach cancer

## Abstract

**Background:**

In Japan, a high-sodium diet is the most important dietary risk factor and is known to cause a range of health problems. This study aimed to forecast Japan’s disability-adjusted life year (DALYs) for chronic diseases that would be associated with high-sodium diet in different future scenarios of salt intake. We modelled DALY forecast and alternative future scenarios of salt intake for cardiovascular diseases (CVDs), chronic kidney diseases (CKDs), and stomach cancer (SC) from 2017 to 2040.

**Methods:**

We developed a three-component model of disease-specific DALYs: a component on the changes in major behavioural and metabolic risk predictors including salt intake; a component on the income per person, educational attainment, and total fertility rate under 25 years; and an autoregressive integrated moving average model to capture the unexplained component correlated over time. Data on risk predictors were obtained from Japan’s National Health and Nutrition Surveys and from the Global Burden of Disease Study 2017. To generate a reference forecast of disease-specific DALY rates for 2017–2040, we modelled the three diseases using the data for 1990–2016. Additionally, we generated better, moderate, and worse scenarios to evaluate the impact of change in salt intake on the DALY rate for the diseases.

**Results:**

In our reference forecast, the DALY rates across all ages were predicted to be stable for CVDs, continuously increasing for CKDs, and continuously decreasing for SC. Meanwhile, the age group-specific DALY rates for these three diseases were forecasted to decrease, with some exceptions. Except for the ≥70 age group, there were remarkable differences in DALY rates between scenarios, with the best scenario having the lowest DALY rates in 2040 for SC. This represents a wide scope of future trajectories by 2040 with a potential for tremendous decrease in SC burden.

**Conclusions:**

The gap between scenarios provides some quantification of the range of policy impacts on future trajectories of salt intake. Even though we do not yet know the policy mix used to achieve these scenarios, the result that there can be differences between scenarios means that policies today can have a significant impact on the future DALYs.

## Background

High-sodium diet has been confirmed to be associated with the risk of cardiovascular diseases (CVDs); chronic kidney diseases (CKDs); and neoplasms, including stomach cancer (SC) [1, 2]. Disability-adjusted life year (DALY) is a comprehensive measurement of health gaps that represents premature mortality in populations along with the extent and the severity of morbidities. One DALY is the equivalent of losing 1 year in good health due to illnesses or health conditions. In Japan, of the 67 risk factors covered in the latest Global Burden of Disease Study (GBD) in 2017 (e.g. behavioural, metabolic, environmental, and occupational factors), high-sodium diet was the fifth highest risk factor for DALYs after smoking, high blood pressure, high fasting plasma glucose, and high body mass index (BMI). A high-sodium diet accounted for 3.8% of total DALYs in Japan in 2017 [2], and it was the most significant dietary risk factor affecting DALYs compared with diets low in whole grains, fruits, nuts and seeds, and vegetables [3].

High salt intake is more likely to be seen in Japan than in other countries [4]. Health Japan 21 (the second term), which was formulated in 2012 to improve the lifestyle and to extend healthy life expectancy of the population, sets a goal of consuming less than 8.0 g of salt per day [5]. While this value is much larger than the World Health Organization (WHO)‘s recommendation of less than 5.0 g per day [6], it seems reasonable given that the latest National Health and Nutrition Survey (NHNS) in 2016 showed that the average daily intake of salt in the Japanese population was 9.9 g (10.8 g for men and 9.1 g for women) [7]. These are far from the target of Health Japan 21 (the second term) as well as that of the WHO.

Daily salt intake has decreased by about 1 g over the past 10 years in Japan, but the speed of decline has stagnated [7]. Similarly, while the rate of DALYs attributable to a high-sodium diet has also decreased by approximately 15% over the past 10 years, it has levelled off in the recent years [2]. A high-sodium diet is a modifiable risk factor, and the management of this risk factor is an important intervention to prevent the onset of diseases, prolong healthy life, and efficiently maintain and improve population health [8]. Accordingly, this study aimed to model and forecast Japan’s DALYs for chronic diseases that would be, by scientific consensus, associated with high-sodium diet in different future scenarios of salt intake up to 2040. The ultimate goal of the study is to provide an empirical basis to support future salt interventions in Japan.

## Methods

### Overall forecasting model structure

To generate predictions from 2017 to 2040, we used data from 1990 to 2016 and modelled three disease groups that have been found to be associated with a high-sodium diet by the GBD: CVDs and CKDs from level 2 and SC from level 3 of the GDB hierarchy casual structure. The GBD’s latest study (GBD 2017) covered 195 countries and territories (including Japan) and estimated DALYs and other health metrics for 359 diseases and injuries for each year from 1990 until 2017. The results of the GBD study are available by county and the estimates have been widely used by researchers for scientific studies, and policy-makers and several other stakeholders to argue for decision making, prioritization, and strategic resource allocation [9–16]. The GBD hierarchy causal structure ranges from level 1 to 4. The three cause groups at level 1 are communicable, maternal and neonatal conditions, and nutritional deficiencies; non-communicable diseases; and injuries. These are broken down into 22 diseases and injury categories as level 2 of causes, which are then further disaggregation into level 3, and finally into level 4 of causes with the most detailed 293 diseases groups. Ischaemic heart disease, for example, is classified as non-communicable diseases (level 1), cardiovascular diseases (level 2), cerebrovascular diseases (level 3), and ischaemic heart disease (level 4).

Following the GBD’s forecasting study methodology [17], we developed a three-component model of disease-specific DALYs for the three diseases associated with high salt intake. The model consists of a component on the changes in major behavioural and metabolic risk predictors including salt intake as a main risk predictor of interest in this study; a component on the income per capita, educational attainment, and total fertility rate under 25 years, which were combined into a socio-demographic index (SDI) expressed on a scale from 0 to 1, and time; and an autoregressive integrated moving average (ARIMA) model that captures the unexplained component correlated with time course. Further detail, including data sources, and model formulae are described below.

### DALYs and SDI data, 1990–2016

We used the estimates of DALY rate per 100,000 population for CVDs, CKDs, and SC as well as SDI in Japan for the years 1990–2016 published in GBD 2017 [18]. The detailed methodologies for estimating DALYs and SDI are provided in the GBD 2017 summary publications [18, 19]. Data extraction and analysis were performed by sex (men, women, and both sexes combined) and age group (20–49, 50–69, ≥70 years, and all ages). The 0–19 years age group was not considered due to a lack of risk predictor data (see below).

### Behavioural and metabolic risk predictor data, 1990–2016

We considered the population level average of salt intake (grams per day) and the prevalence of current smokers, current alcohol drinkers, and obesity for each sex and age groups based on the availability of data. These were obtained from Japan’s NHNS for 1990–2016 by sex and age groups. The NHNS is a nationally representative household survey, which is conducted annually by the Japanese Ministry of Health, Labour and Welfare to clarify dietary habits, nutrition intake, and lifestyle at the population level in Japan [20]. The NHNS consists of three parts: 1) physical tests, such as a blood test, performed by physicians at community centres; 2) an in-person survey of a (weighted) single-day dietary record of households; and 3) a self-reported lifestyle questionnaire (including smoking status and alcohol consumption) accompanying the dietary survey. No urine sodium was measured by NHNS. Detailed descriptions of survey procedures of NHNS are available elsewhere [20, 21]. To explain briefly, dietary intake data survey was conducted on a designated day excluding Sundays and public holidays. Trained interviewers (mainly registered dietitians) instructed household representatives (usually those who were responsible for food preparation) on how to measure food and beverage quantities consumed by the household members using an open-ended recording form. The allocation of shared dishes taken by each household members, food waste, leftovers, and foods eaten away from home were also recorded, as well as the portion size consumed or quantity of foods when weighing was not possible. The trained interviewers visited each household to check the participants’ survey compliance and, if necessary, confirmed portion sizes and converted estimates of portion sizes or quantity of foods. Each food item was coded according to the dietary record and the corresponding food composition list in the sixth edition of the Japanese Standard Tables of Food Composition [22].

In this study, salt intake (grams per day) was calculated as sodium (mg) × 2.54/1000. Obesity was defined as BMI of ≥25 kg/m^2^ according to the Japan Society for the Study of Obesity [23, 24]. Only those aged 20 years or older were considered in this study because the lifestyle questionnaire was not administered to the younger population aged less than 20 years old. Analytic sample sizes ranged from 6149 to 26,594 between 1990 and 2016. A total of 275,468 (127,571 men and 147,897 women) who were aged 20 or older and who completed the salt intake assessment were used to calculate the average daily salt intake and the prevalence of other predictors from the individual surveys.

### ARIMA model for forecast

The ARIMA model was used to forecast future DALY rates with adjustment for several risk predictors. It produces forecasts based on its own past values in the time series (an autoregressive: AR term) with the error made by previous predictions (a moving average: MA term) using the shift and lag of historical information. Integrated (I term) in ARIMA model represents the differencing of raw observed data in order to make the time series stationary, that is, data values are replaced by the difference from the previous values.

In the ARIMA model, a standard notation is ARIMA with p, d, and q, where integer values substitute for the parameters to represent the type of the model described as ARIMA (p, d, q). The parameter is defined as follow: p is the order of the autoregressive; d is the degree of differencing involved; and q is the order of the moving average. Zero value can be used as a parameter, indicating that a particular component is not used in the model. For example, ARIMA (1, 0, 2) indicates no differencing, one AR term and two MA terms in the model. As generally known, the ARIMA model is given by

1 $$ \left(1-\sum \limits_{i=1}^p{\alpha}_i{L}^i\right){\left(1-L\right)}^d{y}_t=\left(1+\sum \limits_{i=1}^q{\beta}_i{L}^d\right){\varepsilon}_t,\kern0.5em $$

where *y*_*t*_ is the outcome of interest; *ε*_*t*_ is an (white noise) error term, which is the residual defined as a time series of the difference between an observed and a predicted value at time *t*; *L* is time lag operator defined as *L*^*k*^*y*_*t*_ = *y*_*t* − *k*_; and *α*_*i*_ and *β*_*i*_ are the *i*th coefficient parameters of p (AR part) and q (MA part) [25]. The key point is that the time series model should have a serial correlation in the observed data, thus the residuals themselves are independent and identically distributed with zero mean and covariance. If the left-hand side of the Eq. (1) contains the differenced value, appropriate adjustments were also applied on the right-hand side. Before fitting the models, the stationary state of observed data in the series, which means there is constancy to the data over time, was examined using Dickey-Fuller test [25]. If non-stationary was assumed to be plausible, the data were transformed into a stationary time series by taking a suitable difference with order d. The autocorrelation function and partial autocorrelation function were used to identify the stationary status and to decide the range of grid search for the orders of the models. Model parameters were estimated by using maximum likelihood methods. Akaike’s Information Criterion (AIC) was calculated to select optimal models with the orders.

A two-step approach was used to forecast the future DALY rates: the first step was to independently forecast the values of each predictor at the population level (i.e. SDI, the average salt intake (grams per day), the prevalence of obesity (%), current smoker (%), and current alcohol drinker (%)) from 2017 until 2040 using the Equation (1), and the second step was to forecast the log-scaled DALYs rates *y*_*t*_ by using the following Equation (2) after plugging the predicted values of the above predictors into *x*_*tj*_:

2 $$ \left(1-\sum \limits_{i=1}^p{\alpha}_i{L}^i\right){\left(1-L\right)}^d{y}_t=\sum \limits_{j=1}^4{\gamma}_j{L}^d{x}_{tj}+\left(1+\sum \limits_{i=1}^q{\beta}_i{L}^d\right){\varepsilon}_t, $$

where *x*_*tj*_ is the value of *j* th predictor at time *t*, and *γ*_*j*_ is a coefficient parameter for the *j* th predictor. Equation (2) is the general form of so-called ARIMAX model (ARIMA with eXogeneous inputs), which capture the influence of external factors [26]. As widely adopted in epidemiological time series studies [27–29], ARIMAX has the capacity to generate predictions while identifying the underlying patterns of changes of both internal and external nature. All analyses were conducted using R version 3.6.1. The sets of parameters in Equation (2) were separately estimated by age and sex categories.

### Future scenarios for salt intake

We assumed several future scenarios to evaluate the impact of salt intake on the DALY rates for the three diseases (CVDs, CKDs, and SC) for 2017–2040: a reference forecasts and three alternative scenarios (best, moderate, worse). The reference forecast assumed that the current trend is maintained: i.e. future salt intake during 2017–2040 was predicted using the ARIMA model defined in Equation (1). In the best scenario, a target daily salt intake (8 g per day) will be achieved in 2023 as per Health Japan 21 (the second term) targets [5] and continue to decline to reach 5 g per day in 2040 as per WHO’s guideline [6]. This scenario assumed a constant monotonic decreasing function from 2017 to 2023, when the 8 g per day is achieved, and a further monotonic decreasing function from 2024 to 2040, when 5 g per day is achieved. The moderate scenario assumed that less than the 8.0 g per day set in the best scenario is achieved in 2040 rather than in 2023, with a monotonic decrease function. The worse scenario is where the most recent salt intake (i.e. the value in 2016) remains constant through 2017 to 2040. By entering these assumed scenario values into the Equation (2) as a predictor, we can then obtain the final prediction value of DALY rates until 2040 for these alternative scenarios. Note that the salt intake as of 2040 is the same by definition in the best and moderate scenarios, and the predicted DALY rates converge mathematically to the same values in 2040.

## Results

While the average salt intake and the prevalence of current smokers and alcohol drinkers have declined since 1990, SDI and the prevalence of obesity have increased. Table 1 summarizes the sex- and age group-specific SDI and the behavioural and metabolic risk predictor data in 1990–2016.

**Table 1:** Sex- and age group-specific DALY rate, SDI, and behavioural and metabolic risk predictor data

		GBD 2017 data				NHNS data					
		All ages DALYs rate per 100,000 population [18]			SDI	Number of NHNS participant	Mean age (SD)	Salt intake (g/day)	Obesity (%)	Current smoker (%)	Current alcohol drinker (%)
Year	Sex	Cardiovascular diseases	Chronic kidney diseases	Stomach cancer							
1990	Men	4649.6	400.2	1164.3	0.803	6182	47.4 (16.1)	14.3	22.3	53.1	52.1
	Women	3674.8	378.3	622.9	0.803	7025	48.7 (16.8)	11.8	21.8	9.7	6.1
	Both sexes combined	4154.1	389.1	889.1	0.803	13,207	48.1 (16.5)	13.0	22.0	28.5	26.0
1995	Men	4626.0	458.2	1164.2	0.823	4976	47.8 (16.5)	15.0	23.9	52.7	54.4
	Women	3573.7	412.2	579.8	0.823	5766	49.0 (17.3)	13.0	20.9	10.7	7.4
	Both sexes combined	4090.4	434.8	866.8	0.823	10,742	48.4 (17.0)	13.9	22.2	28.2	27.0
2000	Men	4523.5	497.9	1117.1	0.834	4513	50.1 (17.0)	13.8	26.8	47.4	50.8
	Women	3344.3	427.2	520.1	0.834	5149	51.7 (17.5)	12.2	21.3	11.5	9.0
	Both sexes combined	3922.2	461.9	812.7	0.834	9662	50.8 (17.2)	12.9	23.8	27.0	27.0
2005	Men	4646.2	528.7	1077.1	0.844	3591	52.5 (17.5)	12.4	28.6	39.3	36.7
	Women	3313.6	439.7	470.2	0.844	4155	54.5 (17.8)	10.7	22.0	11.4	7.4
	Both sexes combined	3964.9	483.2	766.8	0.844	7746	53.6 (17.7)	11.5	24.9	24.3	20.9
2010	Men	4664.1	577.6	1017.6	0.853	3740	53.9 (17.5)	11.4	30.4	32.2	35.4
	Women	3300.7	466.2	424.7	0.853	4239	55.6 (17.8)	9.8	21.1	8.4	7.0
	Both sexes combined	3965.6	520.5	713.8	0.853	7746	54.8 (17.7)	10.6	25.3	19.5	20.3
2016	Men	4590.3	555.3	931.8	0.863	12,132	56.6 (17.6)	10.8	31.7	30.5	34.0
	Women	3400.8	480.5	385.1	0.863	14,010	58.1 (18.0)	9.2	21.3	7.6	7.5
	Both sexes combined	3980.4	517.0	651.5	0.863	26,142	57.4 (17.8)	9.9	25.9	18.2	19.8

*GBD* Global Burden of Disease study, *NHNS* National Health and Nutrition Survey of Japan, *DALYs* Disability-adjusted life years, *SDI* Socio-demographic index, *SD* Standard deviation. Note that we used data for each year, but the table lists only selected years

Supplementary Table 1 shows the predicted (2017–2040) salt intake for the reference forecast and the three alternative scenarios. Overall, women consumed less salt than men. In 1990, the older population consumed less salt than did the younger population. However, this trend was reversed in 2016, and today, the younger population consumes less salt than the older population. In other words, the rate of decline in salt intake is faster in the younger population. Predicted values for other covariates are presented in Supplementary Table 2.

Future trends of DALY rate for cardiovascular disease, chronic kidney diseases, and stomach cancer.

### Cardiovascular disease

**Fig. 1:**
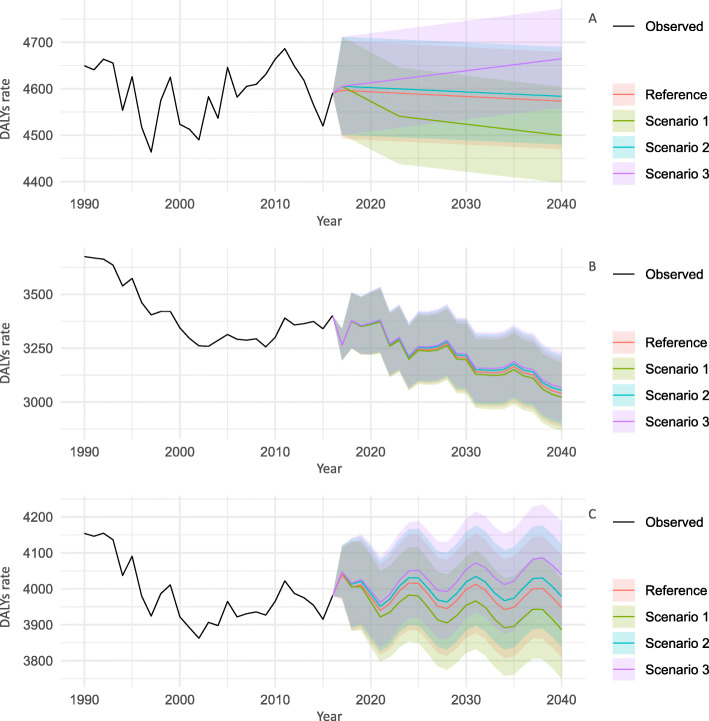
Observed and predicted all-age DALY rate (per 100,000 population) for cardiovascular diseases, 1990–2040: **a** men, **b** women, and **c** both sexes combined. 1: best scenario; 2: moderate scenario; 3: worse scenario. It is important to note that the y-axis scales are different for each panel in order to make the differences between scenarios easier to understand

The estimated sets of parameters (p, d, and q) in the ARIMA models given by the Equation (2) and the associated AIC are shown in Supplementary Table 3. All-age and age-group specific trends of DALY rates for CVDs for 2017–2040 by sex and scenarios are shown in Fig. 1 and Supplementary Figures, respectively (Supplementary Figure 1 for 20–49 years, Supplementary Figure 2 for 50–69 years, and Supplementary Figure 3 for ≥70 years). In all scenarios including reference forecast, the all-age and age group-specific DALY rate estimates until 2040 were not remarkably different, with overlapping prediction intervals (PIs).

The DALY rates continued to decline until 2040 regardless of the scenarios, and the sex, or the age groups. In the reference forecast, the greatest decline in the DALY rates was expected in the ≥70 age group, with a decline of 45.1% for men and 40.9% for women from 2016 to 2040. Meanwhile, in all ages, the sex-combined DALY rates demonstrated a stable time-trend during 2016 to 2040. The difference between the age group-specific estimates and all-age estimates is that the latter was greatly affected by population aging. The exact values of the DALY rates for the three diseases in 2040 for the 20–49, 50–69, and ≥ 70 age groups, and all ages; for men, women, and both sexes combined; and for all scenarios are presented in Supplementary Table 4.

### Chronic kidney diseases

**Fig. 2:**
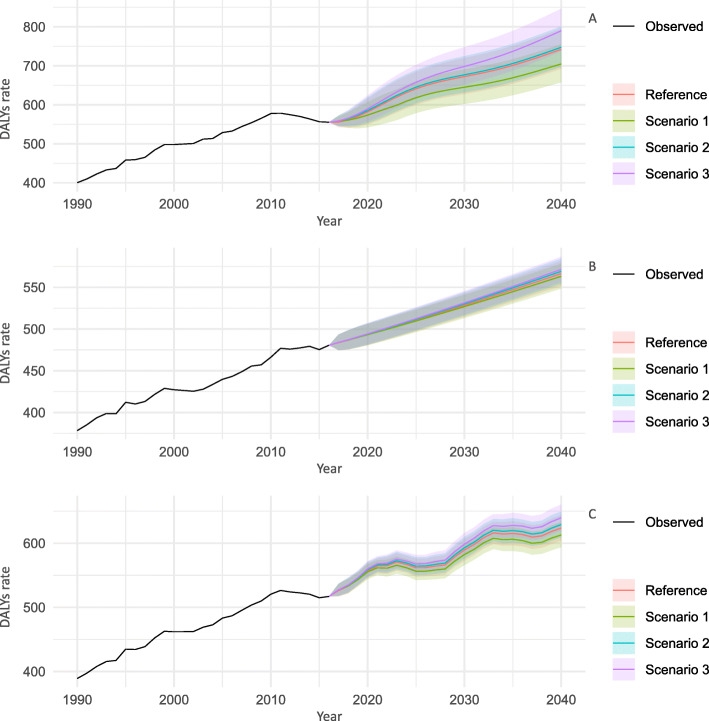
Observed and predicted all-ages DALY rate (per 100,000 population) for chronic kidney diseases, 1990–2040: **a** men, **b** women, and **c** both sexes combined. 1: best scenario; 2: moderate scenario; 3: worse scenario. It is important to note that the y-axis scales are different for each panel in order to make the differences between scenarios easier to understand

Figure 2 shows the trends of all-age DALY rates for CKDs for 2017–2040 by sex and scenarios, while those for age group-specific DALY rates are shown in Supplementary Figures (Supplementary Figure 4 for 20–49 years, Supplementary Figure 5 for 50–69 years, and Supplementary Figure 6 for ≥70 years). As with the trends in CVDs, the all-age and age group-specific DALY rate estimates for CKDs in all scenarios up to 2040 were not remarkably different, with overlapping PIs.

For all scenarios, the age group-specific DALY rates tended to decline until 2040, except in men aged 50–69 years. In the reference forecast, the greatest decline in the DALY rates was expected in men aged 20–49 years and women aged ≥70 years, with an average decline of 18.7 and 21.2% for women between 2016 and 2040, respectively. Meanwhile, in contrast to the trend for each age group, there was an upward trend for all-age DALY rate, suggesting that the aging of the population may have significant effects on the future DALY rates of CKDs.

### Stomach cancer

**Fig. 3:**
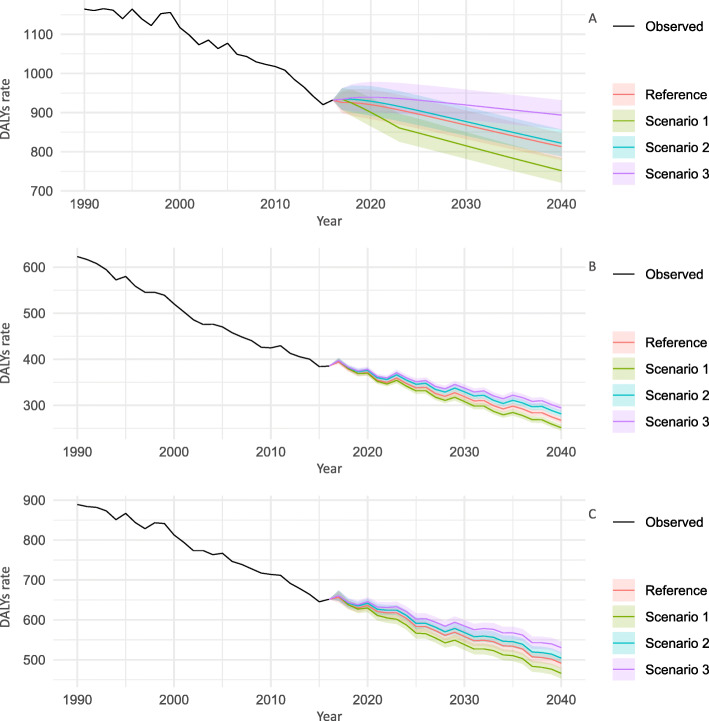
Observed and predicted all ages DALYs rate (per 100,000 population) for stomach cancer, 1990–2040: **a** men, **b** women, and **c** both sexes combined. 1: best scenario; 2: moderate scenario; 3: worse scenario. It is important to note that the y-axis scales are different for each panel in order to make the differences between scenarios easier to understand

Figure 3 shows the trends of the all-age DALY rates for SC for 2017–2040 by sex and scenarios. For both men and women and both sexes combined, the best scenarios had the lowest DALY rates in 2040 (751.6, 95% PIs 720.9–783.7 for men; 251.3, 245.0–257.7 for women; and 465.9, 453.1–479.1 for both sexes combined, respectively) than the reference forecasts (812.8, 779.6–847.5; 267.3, 260.6–274.1; and 491.2, 477.7–505.1, respectively), with declining rates of 19.3, 34.7, and 28.5%, respectively.

All-age and age group-specific DALY rates continued to decline until 2040 regardless of the scenarios or the sex. The trends of the age group-specific DALY rates for SC for 2017–2040 by sex and scenarios are shown in Supplementary Figures (Supplementary Figure 7 for 20–49 years, Supplementary Figure 8 for 50–69 years, and Supplementary Figure 9 for ≥70 years). Except for the ≥70 age group, there were remarkable differences in DALY rates between scenarios, with the best scenario likely to have the lowest DALY rate in 2040. The largest decline in the rates from 2016 to 2040 was observed in the 20–49 age group: 67.3% for men and 59.8% for women. Meanwhile, a significant difference was also observed between the worse scenario and the reference forecast in the predicted DALY rates among these groups.

## Discussion

To guide a long-term investment and policy implementation, it is important to understand the trajectories and drivers of health. In Japan, high salt intake is a national epidemic [30, 31] and plays an important role in addressing the major policy challenge of increasing health care costs due to the aging of the population and the extension of life expectancy [8]. To our best knowledge, this was the first study to forecast a set of disease-, sex-, and age group-specific DALY rates for chronic diseases that would be, by scientific consensus, associated with a high-sodium diet using a framework that allows for exploring different scenarios of salt intake and other independent risk predictors. In our reference forecast, all-age DALY rates were predicted to be stable for CVDs, continue increasing for CKDs, and continue decreasing for SC. Meanwhile, age group-specific DALY rates for these three diseases were forecasted to decrease, except in men aged 50–69 years for CKDs.

For men, women and both sexes combined, aged 20–49 years and 50–69 years, there were massive gaps between the best forecast and other alternative scenarios for the predicted DALY rates for SC, representing a wide scope of future trajectories by 2040 with the potential decrease in SC burden. Meanwhile, the gap between worse scenario and reference forecast for the predicted DALY rates for stomach cancer among these groups indicate alarming challenges if Japan fall behind their reference forecast.

Our findings are in accordance with previous analyses showing an association of lower risk of stomach cancer with lower salt intake [32]. A population reduction in salt intake is recognized as a global priority for a highly cost-effective prevention of the epidemic of chronic diseases regardless of the level of socioeconomic development [33, 34]. Although our models do not conclusively prove the remarkable benefit of reduced salt intake with respect to CVDs and CKDs in Japan, previous studies suggest the potential for a further benefit by the population level reduction of salt intake in addition to its effects on stomach cancer morbidity and mortality [35]. The lack of a remarkable difference in trends of future DALY rates for CVDs and CKDs among the scenarios of salt intake may be because of the fact that many other health drivers are also influential, such as, social determinants of health, making it difficult to see the effects of salt reduction alone.

In Japan, sodium intake is mostly derived from seasonings (e.g. soy sauce, table salt, and miso), bread, noodles, and other processed foods [36, 37]. Using the data of the 2012 NHNS [38], Takimoto et al. reported in 2018 that 82.1% of the Japanese consumed soy sauce on the day of the survey, equivalent to an average salt intake of 2.2 g. Many Japanese also used table salt and miso (81.4 and 47.1%, respectively), which are equivalent to an average salt intake of 1.6 g and 1.9 g, respectively [38]. In addition to these seasonings, approximately 1–3% of the people consumed processed seafood, which corresponded to about 1 g of salt intake, depending on the product [38]. Approximately 1.0% of the people ate instant Chinese noodles, equivalent to 2.2 g of salt intake [38]. These results mean that it is not the food itself, but the way it is cooked or seasoned, that ultimately affects sodium levels in the body. Sodium sources should be explicitly considered when addressing salt reduction.

Health Japan 21 (the second term) not only promotes national action to raise awareness and modify behaviour to reduce salt intake, but also encourages the food industry to reduce salt in processed foods. Although the average salt intake in the Japanese population decreased from 13.0 g per day in 1990 to 9.9 g per day in 2016 [7], this is still far from the target of Health Japan 21 (the second term) as well as that of the WHO. A recent subnational study on salt intake in Japan demonstrated that salt intake is high nationwide and with a small regional variance [39], suggesting that a nationwide strategy would be more effective in reducing salt intake than strategies oriented toward regional differences [39]. For example, Fiji, Hungary, and Portugal have already introduced taxation on high-salt foods [40]. Hungary introduced a “chips tax” on food high in salt in 2011, resulting in the decline in the sales of some food products (e.g. salty snacks, soup, and other powders and artificial seasonings) [41]. It is suggested that the cost effectiveness of a salt taxation would be similar to that of information campaigns and the development of new food products [42]. Although such taxation policies are politically challenging, they could achieve substantial and surprisingly rapid reductions in chronic diseases.

### Limitations

As in any forecasting study, this study is also subject to several limitations. First, many independent risk predictors not included in our model may alter the nature of future health outcome. Therefore, the forecasting effort must recognize that this task is very difficult [17]. For example, the risk of high blood pressure, which is known to be associated with CVDs and CKDs [43–45], was not included in our models to avoid over-adjustment. There is an abundant evidence on a causal relation between salt intake and high blood pressure [46, 47], and therefore, blood pressure can hide the real impact of salt intake if it was included in our model [48]. The effects of high blood pressure on DALY rates for CVDs and CKDs can be partly explained by the changes in BMI and salt intake included in the model [49]. However, other independent effects of high blood pressure have not been considered. Second, our models for forecasting independent risk predictors of DALY rates were relatively simple extrapolations of the past trends that were weighted to some degree for recency. Our forecasts were thus limited by the validity of these simple extrapolations, despite a reasonable AIC-based model selection. In Japan, it has also been suggested that health outcomes may be largely explained by social determinants of health and health system performance [8]. The trajectories of future health outcome may be influenced by these health drivers more than by individual risk factors.

In addition, while DALY is an important population health measure and is widely used as an important benchmark for health policy, it has its limitations [50]. In particular, DALY cannot clearly indicate how much investment is needed to avoid the burden of diseases. It also focuses solely on health and does not capture the broader societal impact of diseases.

Further, our study is also subject to similar limitations as described for other studies concerning dietary patterns [51, 52]. First, in the NHNS, dietary intake was based on a weighted single-day dietary record and may not show reproducibility of long-term dietary patterns. The daily data do not reflect seasonal changes in the dietary patterns. In addition, self-reporting in dietary surveys and lifestyle questionnaires, based on participants’ subjectivity, tends to be accompanied by social desirability and recall biases. Unfortunately, there is no data to deeply test the validity of this response. In addition, reliance on household representatives to record dietary intake during the survey may result in biased estimates of the dietary intake for individual respondents, especially for those who work and eat lunch outside the home (at restaurants, etc.) on weekdays [20]. Finally, there is a possibility that a selection bias was introduced through the stratified two-cluster sampling design in the NHNS. This in turn may have led to biased estimations.

## Conclusion

The gap between scenarios provides a certain quantification of the range of policy impacts on future trajectories of salt intake. We believe that each independent scenario could occur in Japan. Even though we do not yet know the policy mix used to achieve these scenarios, the differences between scenarios indicated by the results in the study illustrates that the policies today can have a significant impact on the future DALYs attributable to a high-sodium diet.

## Supplementary information

**Additional file 1: Supplementary Figure 1.** Observed and predicted DALY rate (per 100,000 population) in the 20–49 age group for cardiovascular diseases for reference forecast and three alternative scenarios, 1990–2040: (A) men, (B) women, and (C) both sexes combined. 1: best scenario; 2: moderate scenario; 3: worse scenario. It is important to note that the y-axis scales are different for each panel in order to make the differences between scenarios easier to understand. **Supplementary Figure 2.** Observed and predicted DALY rate (per 100,000 population) in the 50–69 age group for cardiovascular diseases for reference forecast and three alternative scenarios, 1990–2040: (A) men, (B) women, and (C) both sexes combined. 1: best scenario; 2: moderate scenario; 3: worse scenario. It is important to note that the y-axis scales are different for each panel in order to make the differences between scenarios easier to understand. **Supplementary Figure 3.** Observed and predicted DALY rate (per 100,000 population) in the ≥70 age group for cardiovascular diseases for reference forecast and three alternative scenarios, 1990–2040: (A) men, (B) women, and (C) both sexes combined. 1: best scenario; 2: moderate scenario; 3: worse scenario. It is important to note that the y-axis scales are different for each panel in order to make the differences between scenarios easier to understand. **Supplementary Figure 4.** Observed and predicted DALY rate (per 100,000 population) for chronic kidney diseases in the 20–49 age group for reference forecast and three alternative scenarios, 1990–2040: (A) men, (B) women, and (C) both sexes combined. 1: best scenario; 2: moderate scenario; 3: worse scenario. It is important to note that the y-axis scales are different for each panel in order to make the differences between scenarios easier to understand. **Supplementary Figure 5.** Observed and predicted DALY rate (per 100,000 population) in the 50–69 age group for chronic kidney diseases for reference forecast and three alternative scenarios, 1990–2040: (A) men, (B) women, and (C) both sexes combined. 1: best scenario; 2: moderate scenario; 3: worse scenario. It is important to note that the y-axis scales are different for each panel in order to make the differences between scenarios easier to understand. **Supplementary Figure 6.** Observed and predicted DALY rate (per 100,000 population) in the ≥70 age group for chronic kidney diseases for reference forecast and three alternative scenarios, 1990–2040: (A) men, (B) women, and (C) both sexes combined. 1: best scenario; 2: moderate scenario; 3: worse scenario. It is important to note that the y-axis scales are different for each panel in order to make the differences between scenarios easier to understand. **Supplementary Figure 7.** Observed and predicted DALY rate (per 100,000 population) in the 20–49 age group for stomach cancer for reference forecast and three alternative scenarios, 1990–2040: (A) men, (B) women, and (C) both sexes combined. 1: best scenario; 2: moderate scenario; 3: worse scenario. It is important to note that the y-axis scales are different for each panel in order to make the differences between scenarios easier to understand. **Supplementary Figure 8.** Observed and predicted DALY rate (per 100,000 population) in the 50–69 age group for stomach cancer for reference forecast and three alternative scenarios, 1990–2040: (A) men, (B) women, and (C) both sexes combined. 1: best scenario; 2: moderate scenario; 3: worse scenario. It is important to note that the y-axis scales are different for each panel in order to make the differences between scenarios easier to understand. **Supplementary Figure 9.** Observed and predicted DALY rate (per 100,000 population) in the ≥70 age group for stomach cancer for reference forecast and three alternative scenarios, 1990–2040: (A) men, (B) women, and (C) both sexes combined. 1: best scenario; 2: moderate scenario; 3: worse scenario. It is important to note that the y-axis scales are different for each panel in order to make the differences between scenarios easier to understand. **Supplementary Table 1.** Predicted values of average salt intake (grams per day) for reference forecast and three alternative scenarios, by age groups for men (A), women (B), and both sexes combined (C); and for all ages by sex (D), 2017–2040**.** 1: best scenario; 2: moderate scenario; 3: worse scenario. **Supplementary Table 1.** Predicted values of covariates by age groups for men (A), women (B), and both sexes combined (C); and for all ages by sex (D), 2017–2040. SDI: socio-demographic index. **Supplementary Table 2.** Estimated sets of parameters in ARIMA (p, d, q) and Akaike Information Criteria (AIC). **Supplementary Table 3.** Projected DALY rates for cardiovascular diseases (1), chronic kidney diseases (2), and stomach cancer (3) by age groups for men (A), women (B), and both sexes combined (C); and for all ages by sex (D), 2017–2040. 1: best scenario; 2: moderate scenario; 3: worse scenario. **Supplementary Table 4.** Predicted DALY rates (95% prediction intervals) for cardiovascular diseases (1), chronic kidney diseases (2), and stomach cancer (3) by age groups for men (A), women (B), and both sexes combined (C); and for all ages by sex (D), 2017–2040**.**

## Data Availability

All data generated or analysed during this study are included in this published article [and its supplementary information files].

## Abbreviations

AIC: Akaike’s Information Criterion
ARIMA: Autoregressive integrated moving average
BMI: Body mass index
CVDs: Cardiovascular diseases
CKDs: Chronic kidney diseases
DALYs: Disability-adjusted life years
GBD: Global Burden of Disease
NHNS: National Health and Nutrition Survey
PIs: Prediction intervals
SC: Stomach cancer
SDI: Socio-demographic index
WHO: World Health Organization

## References

[1] World Cancer Research Fund and American Institute for Cancer Research (2007). Food, nutrition, physical activity, and the prevention of Cancer: a global perspective.

[2] GBD 2017 Risk Factor Collaborators (2018). Global, regional, and national comparative risk assessment of 84 behavioural, environmental and occupational, and metabolic risks or clusters of risks for 195 countries and territories, 1990–2017: A systematic analysis for the global burden of disease study 2017. Lancet.

[3] GBD 2017 Diet Collaborators (2019). Health effects of dietary risks in 195 countries, 1990–2017: A systematic analysis for the global burden of disease study 2017. Lancet.

[4] Powles J, Fahimi S, Micha R (2013). Global, regional and national sodium intakes in 1990 and 2010: a systematic analysis of 24 h urinary sodium excretion and dietary surveys worldwide. BMJ Open.

[5] MHLW (2012). A basic direction for comprehensive implementation of National Health Promotio.

[6] WHO (2012). Guideline: Sodium intake for adults and children.

[7] MHLW (2017). The National Health and nutrition survey in Japan, 2016.

[8] Nomura S, Sakamoto H, Glenn S (2017). Population health and regional variations of disease burden in Japan, 1990-2015: a systematic subnational analysis for the global burden of disease study 2015. Lancet.

[9] Achoki T, Miller-Petrie MK, Glenn SD (2019). Health disparities across the counties of Kenya and implications for policy makers, 1990-2016: a systematic analysis for the global burden of disease study 2016. Lancet Glob Health.

[10] GBD Brazil Collaborators (2018). Burden of disease in Brazil, 1990–2016: A systematic subnational analysis for the global burden of disease study 2016. Lancet.

[11] Misganaw A, Melaku YA, Tessema GA (2017). National disability-adjusted life years (DALYs) for 257 diseases and injuries in Ethiopia, 1990-2015: findings from the global burden of disease study 2015. Popul Health Metrics.

[12] Tessema GA, Laurence CO, Melaku YA (2017). Trends and causes of maternal mortality in Ethiopia during 1990-2013: findings from the global burden of diseases study 2013. BMC Public Health.

[13] Gomez-Dantes H, Fullman N, Lamadrid-Figueroa H (2016). Dissonant health transition in the states of Mexico, 1990-2013: a systematic analysis for the global burden of disease study 2013. Lancet.

[14] Steel N, Ford JA, Newton JN (2018). Changes in health in the countries of the UK and 150 English local authority areas 1990-2016: a systematic analysis for the global burden of disease study 2016. Lancet.

[15] Mboi N, Murty Surbakti I, Trihandini I (2018). On the road to universal health care in Indonesia, 1990-2016: a systematic analysis for the global burden of disease study 2016. Lancet.

[16] Newton JN, Briggs AD, Murray CJ (2015). Changes in health in England, with analysis by English regions and areas of deprivation, 1990-2013: a systematic analysis for the global burden of disease study 2013. Lancet.

[17] Foreman KJ, Marquez N, Dolgert A (2018). Forecasting life expectancy, years of life lost, and all-cause and cause-specific mortality for 250 causes of death: reference and alternative scenarios for 2016-40 for 195 countries and territories. Lancet.

[18] GBD 2017 DALYs and HALE Collaborators (2018). Global, regional, and national disability-adjusted life-years (DALYs) for 359 diseases and injuries and healthy life expectancy (HALE) for 195 countries and territories, 1990–2017: A systematic analysis for the global burden of disease study 2017. Lancet.

[19] GBD 2017 Mortality Collaborators (2018). Global, regional, and national age-sex-specific mortality and life expectancy, 1950–2017: A systematic analysis for the global burden of disease study 2017. Lancet.

[20] Ikeda N, Takimoto H, Imai S, Miyachi M, Nishi N (2015). Data resource profile: the Japan National Health and nutrition survey (NHNS). Int J Epidemiol.

[21] Katanoda K, Matsumura Y (2002). National Nutrition Survey in Japan--its methodological transition and current findings. J Nutr Sci Vitaminol (Tokyo).

[22] MEXT (2015). Standards tables of food composition in Japan – 2015 (seventh revised edition).

[23] Kanazawa M, Yoshiike N, Osaka T, Numba Y, Zimmet P, Inoue S (2005). Criteria and classification of obesity in Japan and Asia-Oceania. World Rev Nutr Diet.

[24] Haneda M, Noda M, Origasa H (2018). Japanese clinical practice guideline for diabetes 2016. Diabetol Int.

[25] Hamilton JD (1994). Princeton University P. Time Series Analysis: Princeton University Press.

[26] Yan L, Wang H, Zhang X, Li MY, He J (2017). Impact of meteorological factors on the incidence of bacillary dysentery in Beijing, China: a time series analysis (1970-2012). PLoS One.

[27] Zhang X, Zhang T, Pei J, Liu Y, Li X, Medrano-Gracia P (2016). Time series Modelling of syphilis incidence in China from 2005 to 2012. PLoS One.

[28] Molina LL, Angon E, Garcia A, Moralejo RH, Caballero-Villalobos J, Perea J (2018). Time series analysis of bovine venereal diseases in La Pampa, Argentina. PLoS One.

[29] Zhao C, Yang Y, Wu S (2020). Search trends and prediction of human brucellosis using Baidu index data from 2011 to 2018 in China. Sci Rep.

[30] Asakura K, Uechi K, Masayasu S, Sasaki S (2016). Sodium sources in the Japanese diet: difference between generations and sexes. Public Health Nutr.

[31] Brown IJ, Tzoulaki I, Candeias V, Elliott P (2009). Salt intakes around the world: implications for public health. Int J Epidemiol.

[32] D'Elia L, Rossi G, Ippolito R, Cappuccio FP, Strazzullo P (2012). Habitual salt intake and risk of gastric cancer: a meta-analysis of prospective studies. Clin Nutr.

[33] Geneau R, Stuckler D, Stachenko S (2010). Raising the priority of preventing chronic diseases: a political process. Lancet.

[34] Asaria P, Chisholm D, Mathers C, Ezzati M, Beaglehole R (2007). Chronic disease prevention: health effects and financial costs of strategies to reduce salt intake and control tobacco use. Lancet.

[35] Capewell S, O'Flaherty M (2011). Rapid mortality falls after risk-factor changes in populations. Lancet.

[36] NIBIOHN (2016). What foodstuffs do Japanese take sodium from? [Japanese].

[37] Anderson CA, Appel LJ, Okuda N (2010). Dietary sources of sodium in China, Japan, the United Kingdom, and the United States, women and men aged 40 to 59 years: the INTERMAP study. J Am Diet Assoc.

[38] Takimoto H, Saito A, Htun NC, Abe K (2018). Food items contributing to high dietary salt intake among Japanese adults in the 2012 National Health and nutrition survey. Hypertens Res.

[39] Uechi K, Asakura K, Masayasu S, Sasaki S (2017). Within-country variation of salt intake assessed via urinary excretion in Japan: a multilevel analysis in all 47 prefectures. Hypertens Res.

[40] Trieu K, Neal B, Hawkes C (2015). Salt reduction initiatives around the world - a systematic review of Progress towards the global target. PLoS One.

[41] WHO Regional Office for Europe (2015). Mapping salt reduction initiatives in the WHO European region.

[42] Selmer RM, Kristiansen IS, Haglerod A (2000). Cost and health consequences of reducing the population intake of salt. J Epidemiol Community Health.

[43] Brunstrom M, Carlberg B (2018). Association of Blood Pressure Lowering with Mortality and Cardiovascular Disease across Blood Pressure Levels: a systematic review and meta-analysis. JAMA Intern Med.

[44] Jafar TH, Stark PC, Schmid CH (2003). Progression of chronic kidney disease: the role of blood pressure control, proteinuria, and angiotensin-converting enzyme inhibition: a patient-level meta-analysis. Ann Intern Med.

[45] Lv J, Ehteshami P, Sarnak MJ (2013). Effects of intensive blood pressure lowering on the progression of chronic kidney disease: a systematic review and meta-analysis. CMAJ.

[46] Meneton P, Jeunemaitre X, de Wardener HE, MacGregor GA (2005). Links between dietary salt intake, renal salt handling, blood pressure, and cardiovascular diseases. Physiol Rev.

[47] He FJ, MacGregor GA (2007). Salt, blood pressure and cardiovascular disease. Curr Opin Cardiol.

[48] Schisterman EF, Perkins NJ, Mumford SL, Ahrens KA, Mitchell EM (2017). Collinearity and causal diagrams: a lesson on the importance of model specification. Epidemiology.

[49] Aburto NJ, Ziolkovska A, Hooper L, Elliott P, Cappuccio FP, Meerpohl JJ (2013). Effect of lower sodium intake on health: systematic review and meta-analyses. BMJ.

[50] Anand S, Hanson K (1997). Disability-adjusted life years: a critical review. J Health Econ.

[51] Kurotani K, Akter S, Kashino I (2016). Quality of diet and mortality among Japanese men and women: Japan public health center based prospective study. BMJ.

[52] Oba S, Nagata C, Nakamura K (2009). Diet based on the Japanese food guide spinning top and subsequent mortality among men and women in a general Japanese population. J Am Diet Assoc.

