# Supplementary material for: Forecasting disability-adjusted life years for chronic diseases: reference and alternative scenarios of salt intake for 2017–2040 in Japan
Source: BMC Public Health. 2020 Sep 29;20:1475. doi: 10.1186/s12889-020-09596-3 (PMC7526266; doi:10.1186/s12889-020-09596-3)
Supplement: Supplementary file 1 — Additional file 1: Supplementary Figure 1. Observed and predicted DALY rate (per 100,000 population) in the 20–49 age group for cardiovascular diseases for reference forecast and three alternative scenarios, 1990–2040: (A) men, (B) women, and (C) both sexes combined. 1: best scenario; 2: moderate scenario; 3: worse scenario. It is important to note that the y-axis scales are different for each panel in order to make the differences between scenarios easier to understand. Supplementary Figure 2. Observed and predicted DALY rate (per 100,000 population) in the 50–69 age group for cardiovascular diseases for reference forecast and three alternative scenarios, 1990–2040: (A) men, (B) women, and (C) both sexes combined. 1: best scenario; 2: moderate scenario; 3: worse scenario. It is important to note that the y-axis scales are different for each panel in order to make the differences between scenarios easier to understand. Supplementary Figure 3. Observed and predicted DALY rate (per 100,000 population) in the ≥70 age group for cardiovascular diseases for reference forecast and three alternative scenarios, 1990–2040: (A) men, (B) women, and (C) both sexes combined. 1: best scenario; 2: moderate scenario; 3: worse scenario. It is important to note that the y-axis scales are different for each panel in order to make the differences between scenarios easier to understand. Supplementary Figure 4. Observed and predicted DALY rate (per 100,000 population) for chronic kidney diseases in the 20–49 age group for reference forecast and three alternative scenarios, 1990–2040: (A) men, (B) women, and (C) both sexes combined. 1: best scenario; 2: moderate scenario; 3: worse scenario. It is important to note that the y-axis scales are different for each panel in order to make the differences between scenarios easier to understand. Supplementary Figure 5. Observed and predicted DALY rate (per 100,000 population) in the 50–69 age group for chronic kidney diseases for reference forecast [file 12889_2020_9596_MOESM1_ESM.pdf]

Supplementary Figure 1: Observed and predicted DALY rate (per 100,000 population) in the 20–49 age group for cardiovascular diseases for reference forecast and three alternative scenarios, 1990–2040: (A) men, (B) women, and (C) both sexes combined

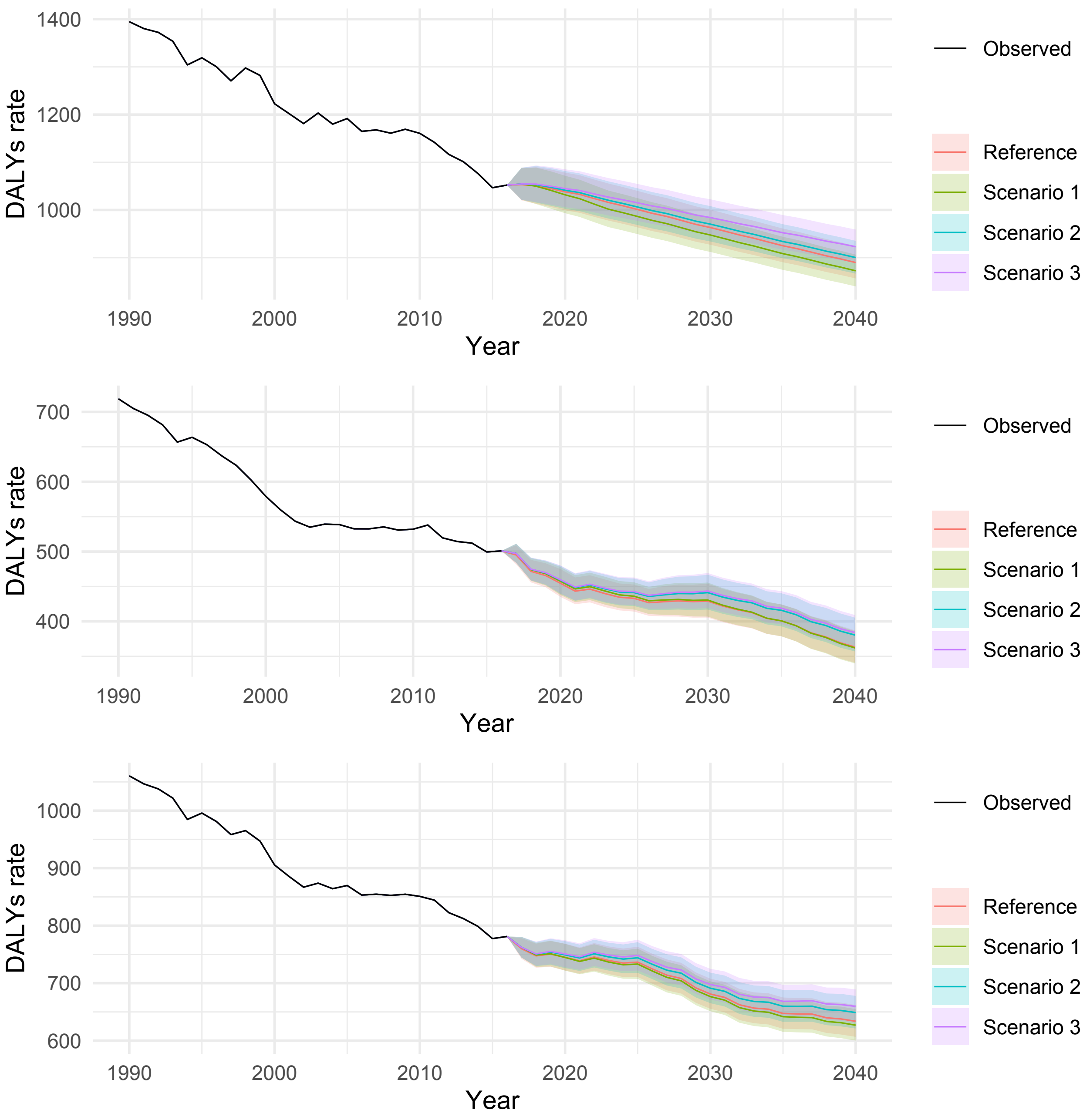

1: best scenario; 2: moderate scenario; 3: worse scenario. It is important to note that the y-axis scales are different for each panel in order to make the differences between scenarios easier to understand.

Supplementary Figure 2: Observed and predicted DALY rate (per 100,000 population) in the 50–69 age group for cardiovascular diseases for reference forecast and three alternative scenarios, 1990–2040: (A) men, (B) women, and (C) both sexes combined

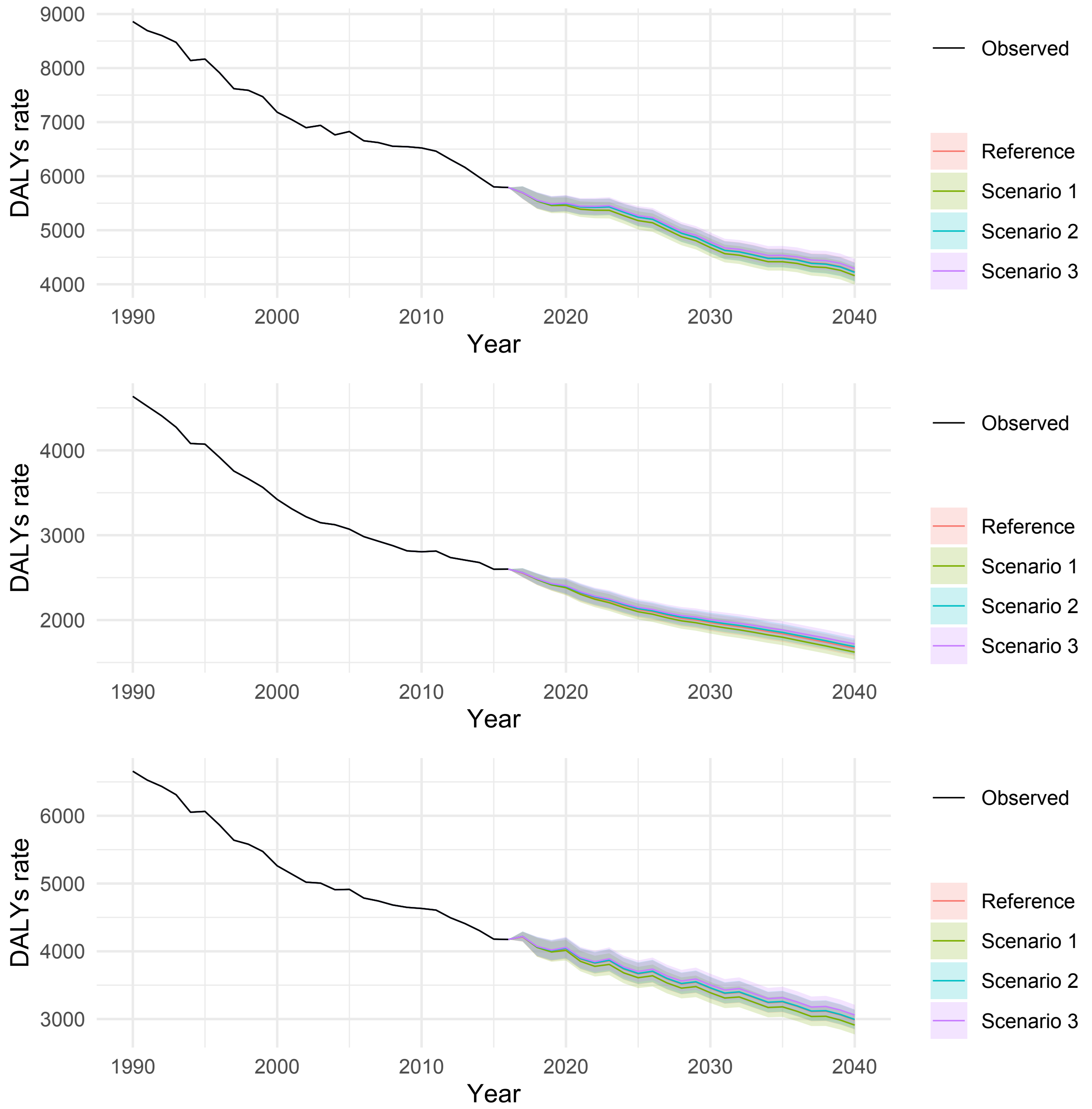

1: best scenario; 2: moderate scenario; 3: worse scenario. It is important to note that the y-axis scales are different for each panel in order to make the differences between scenarios easier to understand.

Supplementary Figure 3: Observed and predicted DALY rate (per 100,000 population) in the ≥70 age group for cardiovascular diseases for reference forecast and three alternative scenarios, 1990–2040: (A) men, (B) women, and (C) both sexes combined

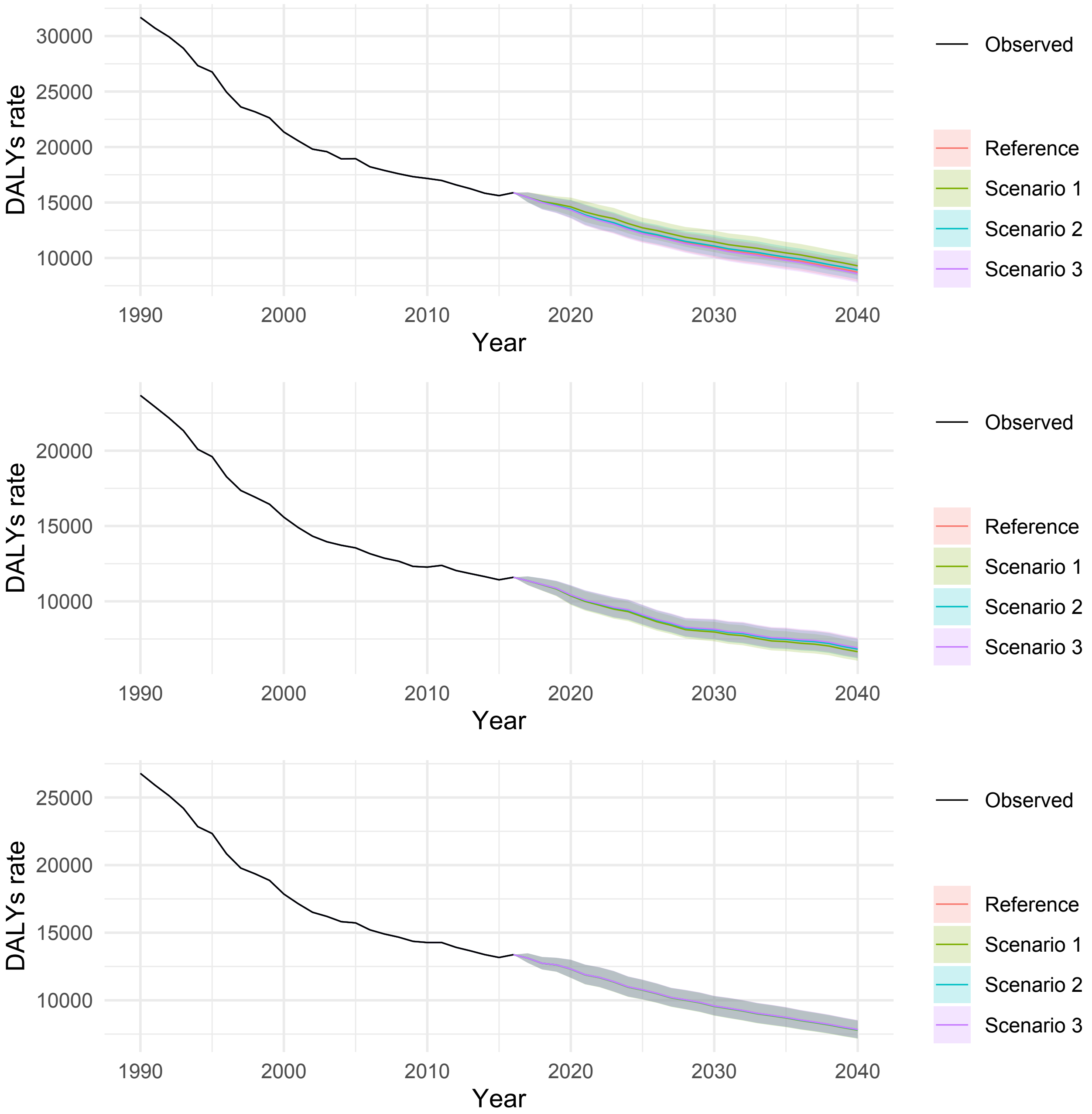

1: best scenario; 2: moderate scenario; 3: worse scenario. It is important to note that the y-axis scales are different for each panel in order to make the differences between scenarios easier to understand.

Supplementary Figure 4: Observed and predicted DALY rate (per 100,000 population) in the 20–49 age group for chronic kidney diseases for reference forecast and three alternative scenarios, 1990–2040: (A) men, (B) women, and (C) both sexes combined

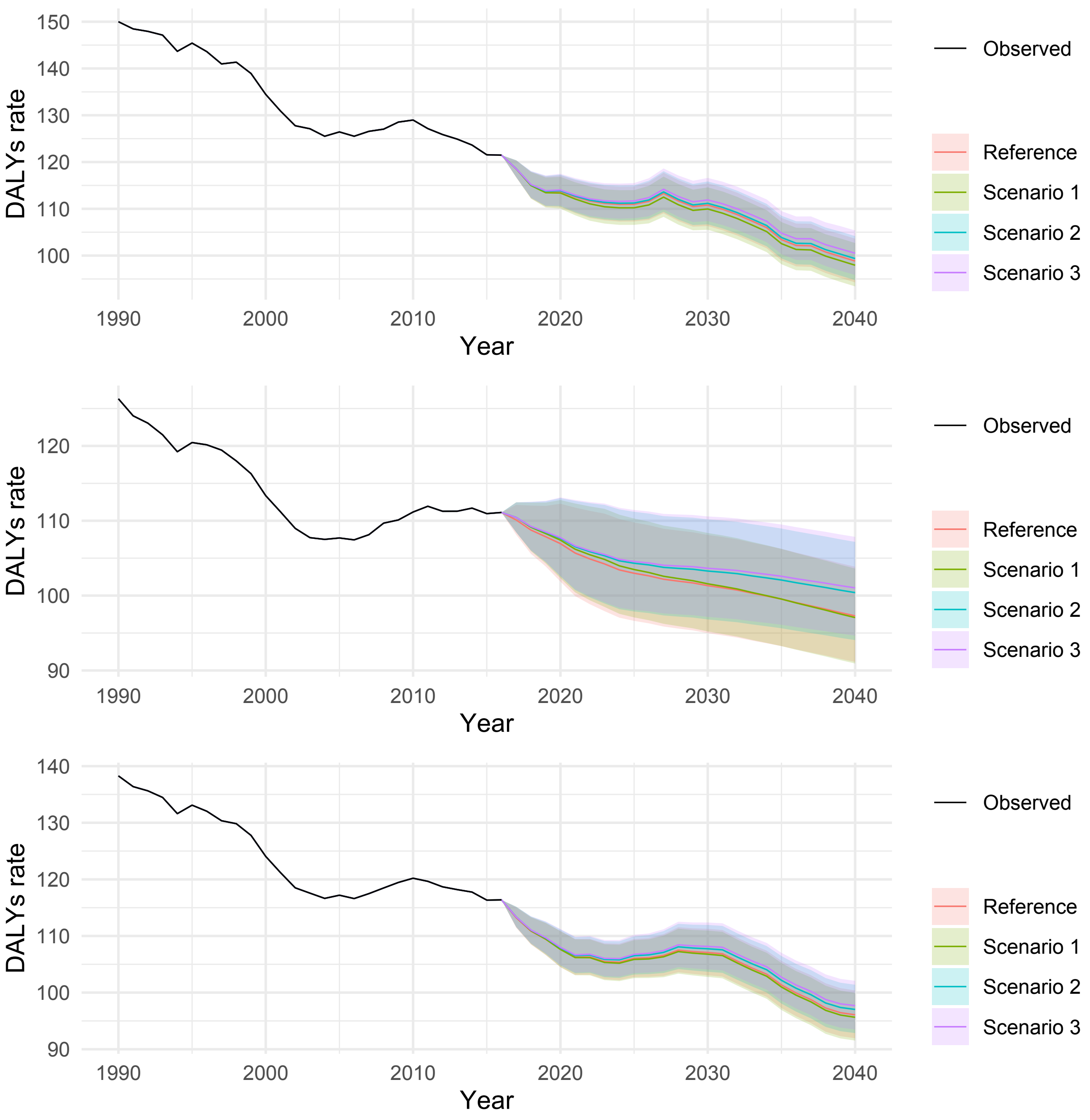

1: best scenario; 2: moderate scenario; 3: worse scenario. It is important to note that the y-axis scales are different for each panel in order to make the differences between scenarios easier to understand.

Supplementary Figure 5: Observed and predicted DALY rate (per 100,000 population) in the 50–69 age group for chronic kidney diseases for reference forecast and three alternative scenarios, 1990–2040: (A) men, (B) women, and (C) both sexes combined

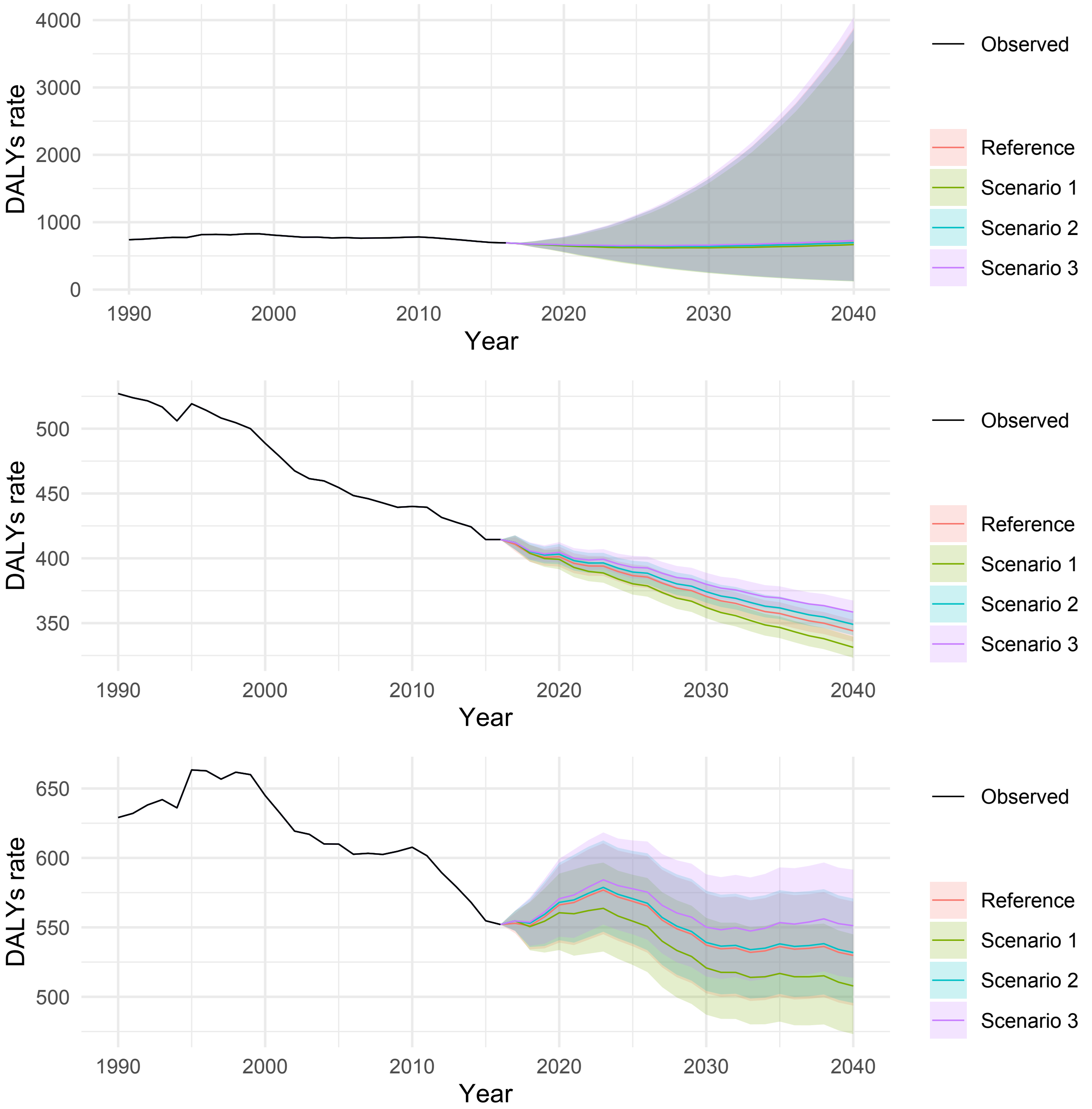

1: best scenario; 2: moderate scenario; 3: worse scenario. It is important to note that the y-axis scales are different for each panel in order to make the differences between scenarios easier to understand.

Supplementary Figure 6: Observed and predicted DALY rate (per 100,000 population) in the ≥70 age group for chronic kidney diseases for reference forecast and three alternative scenarios, 1990–2040: (A) men, (B) women, and (C) both sexes combined

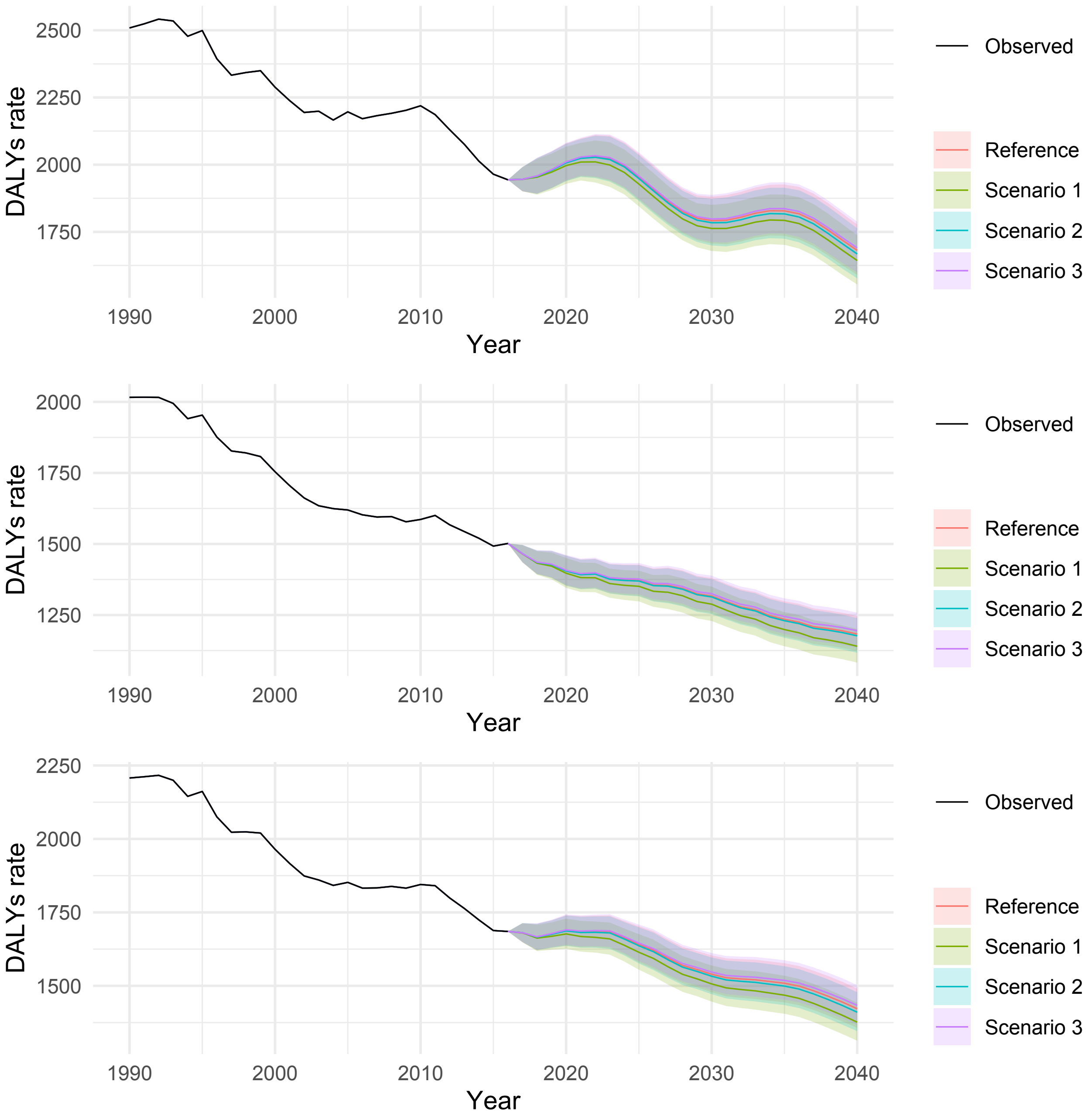

1: best scenario; 2: moderate scenario; 3: worse scenario. It is important to note that the y-axis scales are different for each panel in order to make the differences between scenarios easier to understand.

Supplementary Figure 7: Observed and predicted DALY rate (per 100,000 population) in the 20–49 age group for stomach cancer for reference forecast and three alternative scenarios, 1990–2040: (A) men, (B) women, and (C) both sexes combined

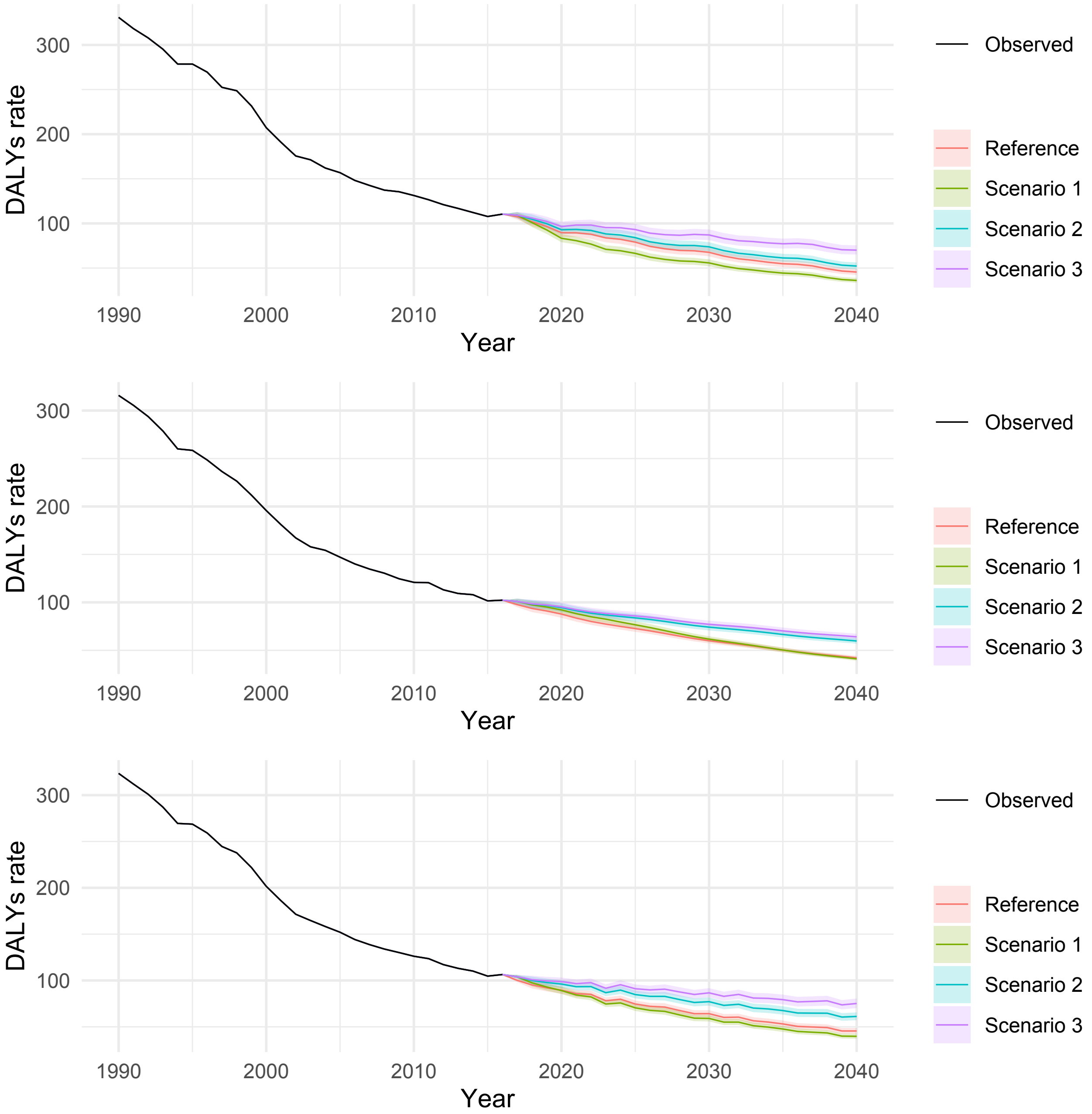

1: best scenario; 2: moderate scenario; 3: worse scenario. It is important to note that the y-axis scales are different for each panel in order to make the differences between scenarios easier to understand.

Supplementary Figure 8: Observed and predicted DALY rate (per 100,000 population) in the 50–69 age group for stomach cancer for reference forecast and three alternative scenarios, 1990–2040: (A) men, (B) women, and (C) both sexes combined

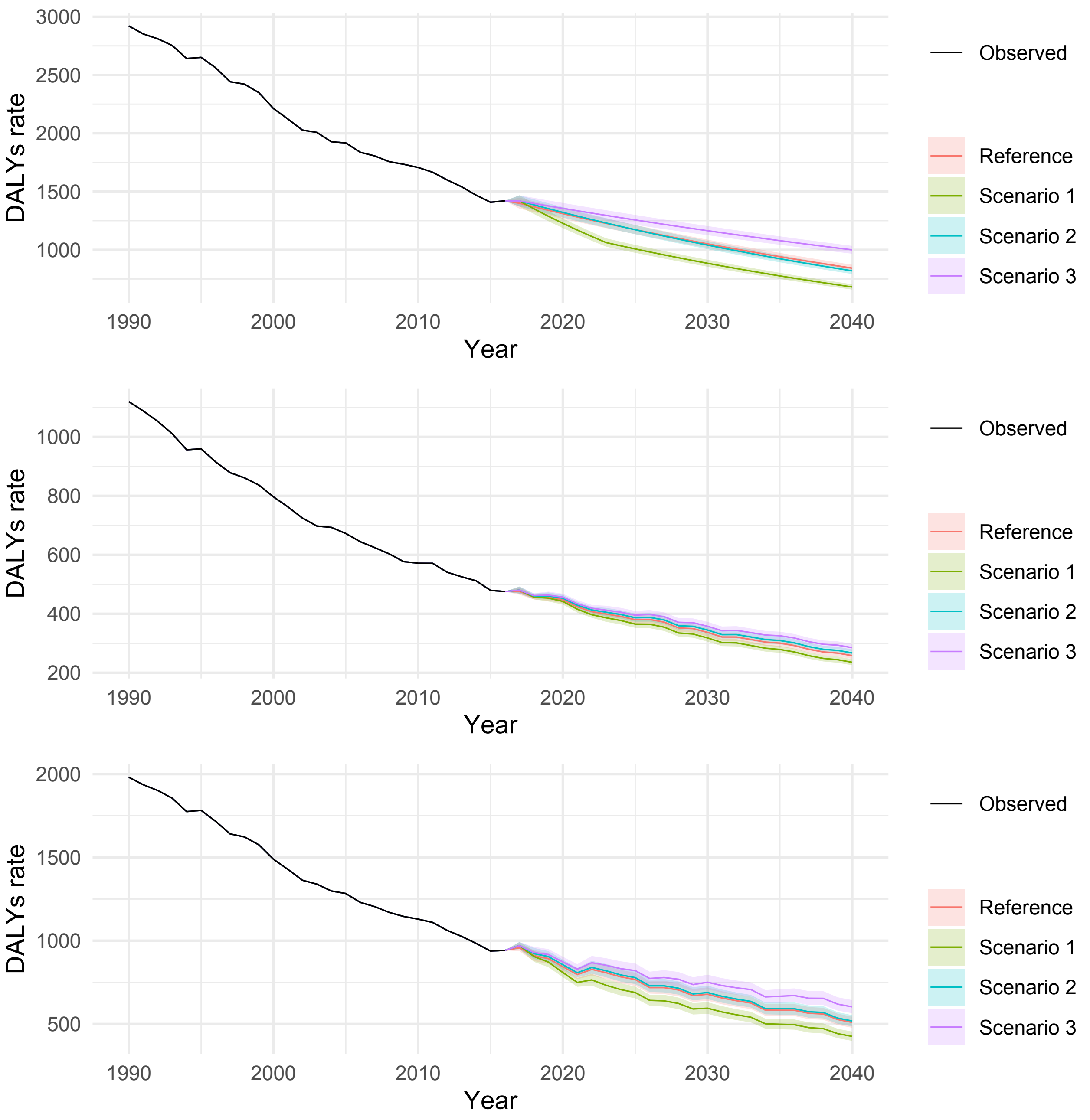

1: best scenario; 2: moderate scenario; 3: worse scenario. It is important to note that the y-axis scales are different for each panel in order to make the differences between scenarios easier to understand.

Supplementary Figure 9: Observed and predicted DALY rate (per 100,000 population) in the ≥70 age group for stomach cancer for reference forecast and three alternative scenarios, 1990–2040: (A) men, (B) women, and (C) both sexes combined

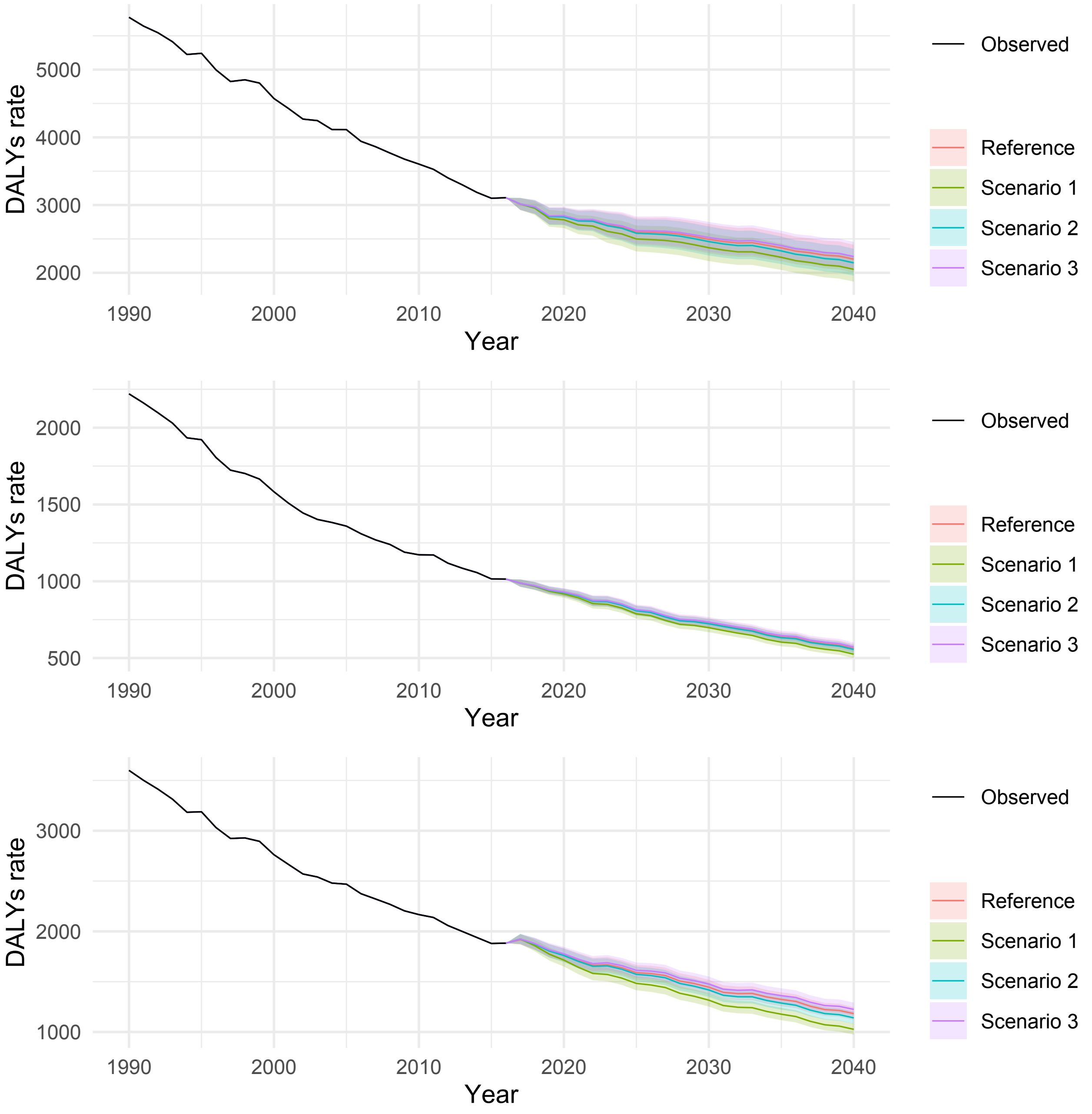

1: best scenario; 2: moderate scenario; 3: worse scenario. It is important to note that the y-axis scales are different for each panel in order to make the differences between scenarios easier to understand.

Supplementary Table 1: Predicted values of average salt intake (grams per day) for reference forecast and three alternative scenarios, by age groups for men (A), women (B), and both sexes combined (C); and for all ages by sex (D), 2017–2040.

	Men (A)											
	20–49				50–69				≥70			
Year	Reference	Scenario 1	Scenario 2	Scenario 3	Reference	Scenario 1	Scenario 2	Scenario 3	Reference	Scenario 1	Scenario 2	Scenario 3
2017	10.3	10.4	10.4	10.4	11.0	11.2	11.2	11.2	10.7	10.8	10.8	10.8
2018	10.1	10.0	10.3	10.4	10.9	10.7	11.1	11.2	10.7	10.3	10.7	10.8
2019	9.9	9.6	10.2	10.4	10.8	10.2	10.9	11.2	10.6	9.9	10.6	10.8
2020	9.8	9.2	10.1	10.4	10.7	9.6	10.8	11.2	10.6	9.4	10.4	10.8
2021	9.6	8.8	10.0	10.4	10.6	9.1	10.7	11.2	10.5	8.9	10.3	10.8
2022	9.5	8.4	9.9	10.4	10.5	8.5	10.5	11.2	10.5	8.5	10.2	10.8
2023	9.3	8.0	9.8	10.4	10.3	8.0	10.4	11.2	10.4	8.0	10.1	10.8
2024	9.2	7.8	9.7	10.4	10.2	7.8	10.2	11.2	10.4	7.8	9.9	10.8
2025	9.1	7.6	9.6	10.4	10.1	7.6	10.1	11.2	10.3	7.6	9.8	10.8
2026	8.9	7.5	9.5	10.4	10.0	7.5	10.0	11.2	10.3	7.5	9.7	10.8
2027	8.8	7.3	9.4	10.4	9.9	7.3	9.8	11.2	10.2	7.3	9.6	10.8
2028	8.6	7.1	9.2	10.4	9.8	7.1	9.7	11.2	10.2	7.1	9.5	10.8
2029	8.5	6.9	9.1	10.4	9.7	6.9	9.5	11.2	10.1	6.9	9.3	10.8

2030	8.3	6.8	9.0	10.4	9.6	6.8	9.4	11.2	10.1	6.8	9.2	10.8
2031	8.2	6.6	8.9	10.4	9.5	6.6	9.3	11.2	10.1	6.6	9.1	10.8
2032	8.0	6.4	8.8	10.4	9.3	6.4	9.1	11.2	10.0	6.4	9.0	10.8
2033	7.9	6.2	8.7	10.4	9.2	6.2	9.0	11.2	10.0	6.2	8.9	10.8
2034	7.8	6.1	8.6	10.4	9.1	6.1	8.8	11.2	9.9	6.1	8.7	10.8
2035	7.6	5.9	8.5	10.4	9.0	5.9	8.7	11.2	9.9	5.9	8.6	10.8
2036	7.5	5.7	8.4	10.4	8.9	5.7	8.6	11.2	9.8	5.7	8.5	10.8
2037	7.3	5.5	8.3	10.4	8.8	5.5	8.4	11.2	9.8	5.5	8.4	10.8
2038	7.2	5.4	8.2	10.4	8.7	5.4	8.3	11.2	9.7	5.4	8.2	10.8
2039	7.0	5.2	8.1	10.4	8.6	5.2	8.1	11.2	9.7	5.2	8.1	10.8
2040	6.9	5.0	8.0	10.4	8.5	5.0	8.0	11.2	9.6	5.0	8.0	10.8

	Women (B)											
	20–49				50–69				≥70			
Year	Reference	Scenario 1	Scenario 2	Scenario 3	Reference	Scenario 1	Scenario 2	Scenario 3	Reference	Scenario 1	Scenario 2	Scenario 3
2017	8.3	8.6	8.6	8.6	9.4	9.5	9.5	9.5	9.4	9.4	9.4	9.4
2018	8.2	8.5	8.5	8.6	9.3	9.3	9.5	9.5	9.3	9.2	9.4	9.4
2019	8.0	8.4	8.5	8.6	9.2	9.0	9.4	9.5	9.3	9.0	9.3	9.4

2020	7.9	8.3	8.5	8.6	9.1	8.8	9.3	9.5	9.3	8.7	9.3	9.4
2021	7.8	8.2	8.5	8.6	9.0	8.5	9.3	9.5	9.2	8.5	9.2	9.4
2022	7.6	8.1	8.4	8.6	8.9	8.3	9.2	9.5	9.2	8.2	9.1	9.4
2023	7.5	8.0	8.4	8.6	8.8	8.0	9.1	9.5	9.1	8.0	9.1	9.4
2024	7.4	7.8	8.4	8.6	8.7	7.8	9.1	9.5	9.1	7.8	9.0	9.4
2025	7.2	7.6	8.4	8.6	8.6	7.6	9.0	9.5	9.1	7.6	8.9	9.4
2026	7.1	7.5	8.3	8.6	8.5	7.5	8.9	9.5	9.0	7.5	8.9	9.4
2027	7.0	7.3	8.3	8.6	8.4	7.3	8.9	9.5	9.0	7.3	8.8	9.4
2028	6.8	7.1	8.3	8.6	8.3	7.1	8.8	9.5	9.0	7.1	8.8	9.4
2029	6.7	6.9	8.3	8.6	8.2	6.9	8.7	9.5	8.9	6.9	8.7	9.4
2030	6.6	6.8	8.2	8.6	8.1	6.8	8.7	9.5	8.9	6.8	8.6	9.4
2031	6.4	6.6	8.2	8.6	8.0	6.6	8.6	9.5	8.8	6.6	8.6	9.4
2032	6.3	6.4	8.2	8.6	7.9	6.4	8.5	9.5	8.8	6.4	8.5	9.4
2033	6.1	6.2	8.2	8.6	7.8	6.2	8.5	9.5	8.8	6.2	8.4	9.4
2034	6.0	6.1	8.1	8.6	7.7	6.1	8.4	9.5	8.7	6.1	8.4	9.4
2035	5.9	5.9	8.1	8.6	7.6	5.9	8.3	9.5	8.7	5.9	8.3	9.4
2036	5.7	5.7	8.1	8.6	7.5	5.7	8.3	9.5	8.7	5.7	8.3	9.4
2037	5.6	5.5	8.1	8.6	7.5	5.5	8.2	9.5	8.6	5.5	8.2	9.4
2038	5.5	5.4	8.0	8.6	7.4	5.4	8.1	9.5	8.6	5.4	8.1	9.4

2039	5.3	5.2	8.0	8.6	7.3	5.2	8.1	9.5	8.5	5.2	8.1	9.4
2040	5.2	5.0	8.0	8.6	7.2	5.0	8.0	9.5	8.5	5.0	8.0	9.4

	Both sexes combined (C)											
	20–49				50–69				≥70			
Year	Reference	Scenario 1	Scenario 2	Scenario 3	Reference	Scenario 1	Scenario 2	Scenario 3	Reference	Scenario 1	Scenario 2	Scenario 3
2017	9.2	9.4	9.4	9.4	10.1	0.3	10.3	10.3	10.0	10.0	10.0	10.0
2018	9.0	9.2	9.4	9.4	10.0	9.9	10.2	10.3	9.9	9.7	9.9	10.0
2019	8.9	9.0	9.3	9.4	9.9	9.5	10.1	10.3	9.9	9.4	9.9	10.0
2020	8.7	8.7	9.2	9.4	9.8	9.2	10.0	10.3	9.8	9.0	9.8	10.0
2021	8.6	8.5	9.2	9.4	9.7	8.8	9.9	10.3	9.8	8.7	9.7	10.0
2022	8.5	8.2	9.1	9.4	9.6	8.4	9.8	10.3	9.8	8.3	9.6	10.0
2023	8.3	8.0	9.1	9.4	9.5	8.0	9.7	10.3	9.7	8.0	9.5	10.0
2024	8.2	7.8	9.0	9.4	9.4	7.8	9.6	10.3	9.7	7.8	9.4	10.0
2025	8.0	7.6	8.9	9.4	9.3	7.6	9.5	10.3	9.6	7.6	9.3	10.0
2026	7.9	7.5	8.9	9.4	9.2	7.5	9.4	10.3	9.6	7.5	9.2	10.0
2027	7.8	7.3	8.8	9.4	9.1	7.3	9.3	10.3	9.6	7.3	9.1	10.0
2028	7.6	7.1	8.7	9.4	9.0	7.1	9.2	10.3	9.5	7.1	9.1	10.0

2029	7.5	6.9	8.7	9.4	8.9	6.9	9.1	10.3	9.5	6.9	9.0	10.0
2030	7.3	6.8	8.6	9.4	8.8	6.8	9.0	10.3	9.4	6.8	8.9	10.0
2031	7.2	6.6	8.6	9.4	8.7	6.6	8.9	10.3	9.4	6.6	8.8	10.0
2032	7.1	6.4	8.5	9.4	8.6	6.4	8.8	10.3	9.4	6.4	8.7	10.0
2033	6.9	6.2	8.4	9.4	8.5	6.2	8.7	10.3	9.3	6.2	8.6	10.0
2034	6.8	6.1	8.4	9.4	8.4	6.1	8.6	10.3	9.3	6.1	8.5	10.0
2035	6.6	5.9	8.3	9.4	8.3	5.9	8.5	10.3	9.2	5.9	8.4	10.0
2036	6.5	5.7	8.2	9.4	8.2	5.7	8.4	10.3	9.2	5.7	8.4	10.0
2037	6.4	5.5	8.2	9.4	8.1	5.5	8.3	10.3	9.2	5.5	8.3	10.0
2038	6.2	5.4	8.1	9.4	8.0	5.4	8.2	10.3	9.1	5.4	8.2	10.0
2039	6.1	5.2	8.1	9.4	7.9	5.2	8.1	10.3	9.1	5.2	8.1	10.0
2040	5.9	5.0	8.0	9.4	7.8	5.0	8.0	10.3	9.0	5.0	8.0	10.0

	All age (D)											
	Men				Women				Both sexes combines			
Year	Reference	Scenario 1	Scenario 2	Scenario 3	Reference	Scenario 1	Scenario 2	Scenario 3	Reference	Scenario 1	Scenario 2	Scenario 3
2017	10.5	10.8	10.8	10.8	9.0	9.2	9.2	9.2	9.7	9.9	9.9	9.9
2018	10.4	10.3	10.7	10.8	8.9	9.0	9.1	9.2	9.6	9.6	9.9	9.9

2019	10.3	9.9	10.6	10.8	8.8	8.8	9.1	9.2	9.5	9.3	9.8	9.9
2020	10.1	9.4	10.5	10.8	8.7	8.6	9.0	9.2	9.4	9.0	9.7	9.9
2021	10.0	8.9	10.3	10.8	8.6	8.4	9.0	9.2	9.2	8.6	9.6	9.9
2022	9.9	8.5	10.2	10.8	8.5	8.2	8.9	9.2	9.1	8.3	9.5	9.9
2023	9.8	8.0	10.1	10.8	8.4	8.0	8.9	9.2	9.0	8.0	9.4	9.9
2024	9.6	7.8	10.0	10.8	8.3	7.8	8.8	9.2	8.9	7.8	9.3	9.9
2025	9.5	7.6	9.8	10.8	8.2	7.6	8.8	9.2	8.8	7.6	9.3	9.9
2026	9.4	7.5	9.7	10.8	8.1	7.5	8.7	9.2	8.7	7.5	9.2	9.9
2027	9.3	7.3	9.6	10.8	8.0	7.3	8.7	9.2	8.5	7.3	9.1	9.9
2028	9.1	7.1	9.5	10.8	7.9	7.1	8.6	9.2	8.4	7.1	9.0	9.9
2029	9.0	6.9	9.3	10.8	7.8	6.9	8.6	9.2	8.3	6.9	8.9	9.9
2030	8.9	6.8	9.2	10.8	7.7	6.8	8.5	9.2	8.2	6.8	8.8	9.9
2031	8.8	6.6	9.1	10.8	7.6	6.6	8.5	9.2	8.1	6.6	8.8	9.9
2032	8.6	6.4	9.0	10.8	7.5	6.4	8.4	9.2	7.9	6.4	8.7	9.9
2033	8.5	6.2	8.9	10.8	7.4	6.2	8.4	9.2	7.8	6.2	8.6	9.9
2034	8.4	6.1	8.7	10.8	7.3	6.1	8.3	9.2	7.7	6.1	8.5	9.9
2035	8.3	5.9	8.6	10.8	7.2	5.9	8.3	9.2	7.6	5.9	8.4	9.9
2036	8.1	5.7	8.5	10.8	7.1	5.7	8.2	9.2	7.5	5.7	8.3	9.9
2037	8.0	5.5	8.4	10.8	6.9	5.5	8.2	9.2	7.4	5.5	8.3	9.9

2038	7.9	5.4	8.2	10.8	6.8	5.4	8.1	9.2	7.2	5.4	8.2	9.9
2039	7.8	5.2	8.1	10.8	6.7	5.2	8.1	9.2	7.1	5.2	8.1	9.9
2040	7.6	5.0	8.0	10.8	6.6	5.0	8.0	9.2	7.0	5.0	8.0	9.9

1: best scenario; 2: moderate scenario; 3: worse scenario

Supplementary Table 2: Predicted values of covariates by age groups for men (A), women (B), and both sexes combined (C); and for all ages by sex (D), 2017–2040.

	Men (A)											
	20–49				50–69				≥70			
Year	SDI	Obesity (%)	Current smoker (%)	Current alcohol drinker (%)	SDI	Obesity (%)	Current smoker (%)	Current alcohol drinker (%)	SDI	Obesity (%)	Current smoker (%)	Current alcohol drinker (%)
2017	0.865	32.0	40.9	26.8	0.865	33.7	31.2	44.5	0.865	24.7	10.9	24.1
2018	0.867	32.2	40.9	25.9	0.867	34.1	30.6	44.5	0.867	24.7	9.9	24.1
2019	0.868	31.8	40.9	24.5	0.868	34.4	30.0	44.5	0.868	24.7	8.9	24.1
2020	0.870	32.6	40.9	22.0	0.870	34.8	29.2	44.5	0.870	24.7	7.9	24.1
2021	0.872	32.9	40.9	23.6	0.872	35.1	28.6	44.5	0.872	24.7	6.9	24.1
2022	0.874	32.9	40.9	21.2	0.874	35.4	27.9	44.5	0.874	24.7	6.0	24.1
2023	0.875	33.4	40.9	19.1	0.875	35.8	27.2	44.5	0.875	24.7	5.0	24.1
2024	0.877	33.8	40.9	19.1	0.877	36.1	26.5	44.5	0.877	24.7	4.0	24.1
2025	0.879	33.9	40.9	18.5	0.879	36.5	25.9	44.5	0.879	24.7	3.0	24.1
2026	0.881	34.3	40.9	17.2	0.881	36.8	25.2	44.5	0.881	24.7	2.0	24.1
2027	0.882	34.6	40.9	18.1	0.882	37.2	24.5	44.5	0.882	24.7	1.0	24.1
2028	0.884	34.9	40.9	16.2	0.884	37.5	23.8	44.5	0.884	24.7	0.0	24.1

2029	0.886	35.2	40.9	14.1	0.886	37.8	23.2	44.5	0.886	24.7	0.0	24.1
2030	0.888	35.5	40.9	14.8	0.888	38.2	22.5	44.5	0.888	24.7	0.0	24.1
2031	0.889	35.8	40.9	13.7	0.889	38.5	21.8	44.5	0.889	24.7	0.0	24.1
2032	0.891	36.1	40.9	12.0	0.891	38.9	21.1	44.5	0.891	24.7	0.0	24.1
2033	0.893	36.4	40.9	11.8	0.893	39.2	20.4	44.5	0.893	24.7	0.0	24.1
2034	0.894	36.7	40.9	9.8	0.894	39.5	19.8	44.5	0.894	24.7	0.0	24.1
2035	0.896	37.0	40.9	8.0	0.896	39.9	19.1	44.5	0.896	24.7	0.0	24.1
2036	0.898	37.3	40.9	8.8	0.898	40.2	18.4	44.5	0.898	24.7	0.0	24.1
2037	0.900	37.6	40.9	7.4	0.900	40.6	17.7	44.5	0.900	24.7	0.0	24.1
2038	0.901	37.9	40.9	5.7	0.901	40.9	17.1	44.5	0.901	24.7	0.0	24.1
2039	0.903	38.2	40.9	5.6	0.903	41.3	16.4	44.5	0.903	24.7	0.0	24.1
2040	0.905	38.5	40.9	4.2	0.905	41.6	15.7	44.5	0.905	24.7	0.0	24.1

	Women (B)											
	20–49				50–69				≥70			
Year	SDI	Obesity (%)	Current smoker (%)	Current alcohol drinker (%)	SDI	Obesity (%)	Current smoker (%)	Current alcohol drinker (%)	SDI	Obesity (%)	Current smoker (%)	Current alcohol drinker (%)
2017	0.865	14.0	11.4	10.7	0.865	21.8	8.3	9.1	0.865	27.2	1.8	1.7

2018	0.867	14.0	11.7	10.7	0.867	21.5	8.3	8.0	0.867	26.5	1.7	1.7
2019	0.868	14.0	12.0	10.7	0.868	21.2	8.3	8.8	0.868	26.5	1.5	1.7
2020	0.870	14.0	12.3	10.7	0.870	20.9	8.3	7.7	0.870	26.5	1.3	1.7
2021	0.872	14.0	12.5	10.7	0.872	20.6	8.3	7.2	0.872	26.5	1.2	1.7
2022	0.874	14.0	12.7	10.7	0.874	20.3	8.3	7.5	0.874	26.5	1.0	1.7
2023	0.875	14.0	12.8	10.7	0.875	20.0	8.3	8.2	0.875	26.5	0.8	1.7
2024	0.877	14.0	12.9	10.7	0.877	19.7	8.3	7.3	0.877	26.5	0.7	1.7
2025	0.879	14.0	13.0	10.7	0.879	19.4	8.3	8.5	0.879	26.5	0.5	1.7
2026	0.881	14.0	13.1	10.7	0.881	19.1	8.3	9.0	0.881	26.5	0.3	1.7
2027	0.882	14.0	13.1	10.7	0.882	18.8	8.3	8.6	0.882	26.5	0.2	1.7
2028	0.884	14.0	13.2	10.7	0.884	18.5	8.3	9.2	0.884	26.5	0.0	1.7
2029	0.886	14.0	13.2	10.7	0.886	18.2	8.3	9.6	0.886	26.5	0.0	1.7
2030	0.888	14.0	13.3	10.7	0.888	17.9	8.3	9.0	0.888	26.5	0.0	1.7
2031	0.889	14.0	13.3	10.7	0.889	17.6	8.3	8.5	0.889	26.5	0.0	1.7
2032	0.891	14.0	13.3	10.7	0.891	17.3	8.3	9.1	0.891	26.5	0.0	1.7
2033	0.893	14.0	13.3	10.7	0.893	17.1	8.3	8.1	0.893	26.5	0.0	1.7
2034	0.894	14.0	13.4	10.7	0.894	16.8	8.3	7.9	0.894	26.5	0.0	1.7
2035	0.896	14.0	13.4	10.7	0.896	16.5	8.3	8.1	0.896	26.5	0.0	1.7
2036	0.898	14.0	13.4	10.7	0.898	16.2	8.3	7.9	0.898	26.5	0.0	1.7

2037	0.900	14.0	13.4	10.7	0.900	15.9	8.3	7.6	0.900	26.5	0.0	1.7
2038	0.901	14.0	13.4	10.7	0.901	15.6	8.3	8.3	0.901	26.5	0.0	1.7
2039	0.903	14.0	13.4	10.7	0.903	15.3	8.3	8.5	0.903	26.5	0.0	1.7
2040	0.905	14.0	13.4	10.7	0.905	15.0	8.3	8.2	0.905	26.5	0.0	1.7

	Both sexes combined (C)											
	20–49				50–69				≥70			
Year	SDI	Obesity (%)	Current smoker (%)	Current alcohol drinker (%)	SDI	Obesity (%)	Current smoker (%)	Current alcohol drinker (%)	SDI	Obesity (%)	Current smoker (%)	Current alcohol drinker (%)
2017	0.865	21.9	25.3	19.8	0.865	28.0	20.1	25.7	0.865	25.3	5.9	11.5
2018	0.867	21.9	25.1	19.8	0.867	27.1	20.1	25.7	0.867	25.3	5.4	11.5
2019	0.868	21.9	25.2	19.8	0.868	28.3	20.1	25.7	0.868	25.3	4.9	11.5
2020	0.870	21.9	25.1	19.8	0.870	27.7	20.1	25.7	0.870	25.3	4.4	11.5
2021	0.872	21.9	25.2	19.8	0.872	27.7	20.1	25.7	0.872	25.3	3.9	11.5
2022	0.874	21.9	25.2	19.8	0.874	27.7	20.1	25.7	0.874	25.3	3.4	11.5
2023	0.875	21.9	25.2	19.8	0.875	27.7	20.1	25.7	0.875	25.3	2.9	11.5
2024	0.877	21.9	25.2	19.8	0.877	27.7	20.1	25.7	0.877	25.3	2.4	11.5
2025	0.879	21.9	25.2	19.8	0.879	27.7	20.1	25.7	0.879	25.3	1.9	11.5

2026	0.881	21.9	25.2	19.8	0.881	27.7	20.1	25.7	0.881	25.3	1.5	11.5
2027	0.882	21.9	25.2	19.8	0.882	27.7	20.1	25.7	0.882	25.3	1.0	11.5
2028	0.884	21.9	25.2	19.8	0.884	27.7	20.1	25.7	0.884	25.3	0.5	11.5
2029	0.886	21.9	25.2	19.8	0.886	27.7	20.1	25.7	0.886	25.3	0.0	11.5
2030	0.888	21.9	25.2	19.8	0.888	27.7	20.1	25.7	0.888	25.3	0.0	11.5
2031	0.889	21.9	25.2	19.8	0.889	27.7	20.1	25.7	0.889	25.3	0.0	11.5
2032	0.891	21.9	25.2	19.8	0.891	27.7	20.1	25.7	0.891	25.3	0.0	11.5
2033	0.893	21.9	25.2	19.8	0.893	27.7	20.1	25.7	0.893	25.3	0.0	11.5
2034	0.894	21.9	25.2	19.8	0.894	27.7	20.1	25.7	0.894	25.3	0.0	11.5
2035	0.896	21.9	25.2	19.8	0.896	27.7	20.1	25.7	0.896	25.3	0.0	11.5
2036	0.898	21.9	25.2	19.8	0.898	27.7	20.1	25.7	0.898	25.3	0.0	11.5
2037	0.900	21.9	25.2	19.8	0.900	27.7	20.1	25.7	0.900	25.3	0.0	11.5
2038	0.901	21.9	25.2	19.8	0.901	27.7	20.1	25.7	0.901	25.3	0.0	11.5
2039	0.903	21.9	25.2	19.8	0.903	27.7	20.1	25.7	0.903	25.3	0.0	11.5
2040	0.905	21.9	25.2	19.8	0.905	27.7	20.1	25.7	0.905	25.3	0.0	11.5

	All age (D)											
	Men				Women				Both sexes combines			

Year	SDI	Obesity (%)	Current smoker (%)	Current alcohol drinker (%)	SDI	Obesity (%)	Current smoker (%)	Current alcohol drinker (%)	SDI	Obesity (%)	Current smoker (%)	Current alcohol drinker (%)
2017	0.865	31.6	28.5	34.0	0.865	20.6	7.7	7.4	0.865	25.2	17.5	19.8
2018	0.867	31.9	27.6	34.0	0.867	20.6	7.8	7.4	0.867	25.2	17.1	19.8
2019	0.868	32.2	26.8	34.0	0.868	20.6	7.7	7.4	0.868	25.2	16.7	19.8
2020	0.870	32.5	25.9	34.0	0.870	20.6	7.8	7.4	0.870	25.2	16.4	19.8
2021	0.872	32.9	25.1	34.0	0.872	20.6	7.7	7.4	0.872	25.2	16.0	19.8
2022	0.874	33.2	24.2	34.0	0.874	20.6	7.7	7.4	0.874	25.2	15.6	19.8
2023	0.875	33.5	23.4	34.0	0.875	20.6	7.7	7.4	0.875	25.2	15.2	19.8
2024	0.877	33.8	22.5	34.0	0.877	20.6	7.7	7.4	0.877	25.2	14.9	19.8
2025	0.879	34.1	21.7	34.0	0.879	20.6	7.7	7.4	0.879	25.2	14.5	19.8
2026	0.881	34.5	20.8	34.0	0.881	20.6	7.7	7.4	0.881	25.2	14.1	19.8
2027	0.882	34.8	19.9	34.0	0.882	20.6	7.7	7.4	0.882	25.2	13.7	19.8
2028	0.884	35.1	19.1	34.0	0.884	20.6	7.7	7.4	0.884	25.2	13.3	19.8
2029	0.886	35.4	18.2	34.0	0.886	20.6	7.7	7.4	0.886	25.2	13.0	19.8
2030	0.888	35.8	17.4	34.0	0.888	20.6	7.7	7.4	0.888	25.2	12.6	19.8
2031	0.889	36.1	16.5	34.0	0.889	20.6	7.7	7.4	0.889	25.2	12.2	19.8
2032	0.891	36.4	15.7	34.0	0.891	20.6	7.7	7.4	0.891	25.2	11.8	19.8

2033	0.893	36.7	14.8	34.0	0.893	20.6	7.7	7.4	0.893	25.2	11.5	19.8
2034	0.894	37.0	14.0	34.0	0.894	20.6	7.7	7.4	0.894	25.2	11.1	19.8
2035	0.896	37.4	13.1	34.0	0.896	20.6	7.7	7.4	0.896	25.2	10.7	19.8
2036	0.898	37.7	12.3	34.0	0.898	20.6	7.7	7.4	0.898	25.2	10.3	19.8
2037	0.900	38.0	11.4	34.0	0.900	20.6	7.7	7.4	0.900	25.2	9.9	19.8
2038	0.901	38.3	10.5	34.0	0.901	20.6	7.7	7.4	0.901	25.2	9.6	19.8
2039	0.903	38.6	9.7	34.0	0.903	20.6	7.7	7.4	0.903	25.2	9.2	19.8
2040	0.905	39.0	8.8	34.0	0.905	20.6	7.7	7.4	0.905	25.2	8.8	19.8

SDI: socio-demographic index

Supplemental Table 3: Estimated sets of parameters in ARIMA (p,d,q) and Akaike Information Criteria (AIC).

Disease	Age group	Sex	p	d	q	AIC
Cardiovascular diseases	20–49	Men	1	0	0	-130.7
		Women	9	0	0	-138.1
		Both sexes combined	7	0	0	-141.0
	50–69	Men	7	0	0	-144.3
		Women	4	0	0	-152.8
		Both sexes combined	3	0	8	-159.1
	≥70	Men	5	0	1	-126.3
		Women	9	0	0	-140.2
		Both sexes combined	4	0	0	-137.0
	All ages	Men	0	0	0	-150.5
		Women	6	0	3	-146.6
		Both sexes combined	4	0	3	-157.4
Chronic kidney disease	20–49	Men	9	0	0	-153.6
		Women	5	0	0	-158.0
		Both sexes combined	7	0	0	-159.6
	50–69	Men	4	0	5	-144.4
		Women	4	0	0	-175.7
		Both sexes combined	5	0	3	-172.1
	≥70	Men	7	0	0	-137.7
		Women	6	0	0	-150.6
		Both sexes combined	4	0	0	-154.2
	All ages	Men	3	0	0	-141.6
		Women	1	0	0	-156.1
		Both sexes combined	7	0	0	-150.4
	20–49	Men	6	0	6	-128.8

Stomach cancer		Women	4	0	3	-137.5
		Both sexes combined	9	0	2	-143.9
	50–69	Men	0	0	0	-128.9
		Women	8	0	0	-148.2
		Both sexes combined	7	0	9	-149.8
	≥70	Men	9	0	0	-124.5
		Women	9	0	0	-144.4
		Both sexes combined	9	0	7	-140.4
	All ages	Men	1	0	0	-134.1
		Women	5	0	0	-174.1
		Both sexes combined	8	0	0	-151.6

62

Supplementary Table 4: Predicted DALY rates (95% prediction intervals) for cardiovascular diseases (1), chronic kidney diseases (2), and stomach cancer (3) by age groups for men (A), women (B), and both sexes combined (C); and for all ages by sex (D), 2017–2040.

	Cardiovascular disease (1)			
	Men (1–A)			
	20–49			
Year	Reference	Scenario 1	Scenario 2	Scenario 3
2017	1053.1 (1020.1–1087.2)	1054.5 (1021.4–1088.5)	1054.5 (1021.4–1088.5)	1054.5 (1021.4–1088.5)
2018	1050.7 (1013.2–1089.7)	1049.9 (1012.4–1088.8)	1053.1 (1015.5–1092.1)	1054.3 (1016.6–1093.3)
2019	1044.9 (1006.3–1085.0)	1041.3 (1002.8–1081.2)	1047.7 (1009.0–1087.9)	1050.0 (1011.2–1090.3)
2020	1038.1 (999.4–1078.3)	1031.8 (993.3–1071.8)	1041.3 (1002.5–1081.7)	1044.7 (1005.7–1085.2)
2021	1032.7 (994.0–1072.9)	1023.7 (985.4–1063.5)	1036.4 (997.6–1076.7)	1040.9 (1001.9–1081.3)
2022	1024.0 (985.7–1063.9)	1012.4 (974.5–1051.8)	1028.1 (989.6–1068.1)	1033.7 (994.9–1073.9)
2023	1015.7 (977.6–1055.3)	1001.5 (964.0–1040.5)	1020.2 (981.9–1059.9)	1026.8 (988.4–1066.8)
2024	1008.6 (970.8–1047.9)	994.2 (956.9–1032.9)	1013.5 (975.5–1052.9)	1021.2 (982.9–1061.0)
2025	1000.9 (963.4–1039.9)	986.3 (949.3–1024.7)	1006.2 (968.5–1045.4)	1014.9 (976.9–1054.5)
2026	993.0 (955.7–1031.6)	978.1 (941.4–1016.2)	998.6 (961.2–1037.5)	1008.4 (970.6–1047.7)
2027	986.4 (949.4–1024.8)	971.3 (934.9–1009.2)	992.4 (955.2–1031.1)	1003.3 (965.7–1042.3)
2028	978.1 (941.4–1016.2)	962.8 (926.7–1000.3)	984.5 (947.6–1022.8)	996.3 (959.0–1035.1)

2029	969.8 (933.4–1007.6)	954.3 (918.6–991.5)	976.5 (939.9–1014.6)	989.3 (952.3–1027.9)
2030	963.2 (927.1–1000.7)	947.5 (912.0–984.4)	970.3 (933.9–1008.1)	984.1 (947.2–1022.4)
2031	955.5 (919.7–992.7)	939.7 (904.5–976.3)	963.0 (926.9–1000.5)	977.8 (941.1–1015.8)
2032	947.6 (912.1–984.5)	931.6 (896.7–967.9)	955.4 (919.6–992.7)	971.1 (934.7–1009.0)
2033	940.7 (905.4–977.3)	924.5 (889.8–960.5)	948.8 (913.3–985.8)	965.5 (929.3–1003.1)
2034	932.7 (897.7–969.0)	916.3 (882.0–952.0)	941.2 (905.9–977.9)	958.7 (922.8–996.1)
2035	924.9 (890.3–960.9)	908.4 (874.3–943.7)	933.7 (898.7–970.1)	952.2 (916.5–989.3)
2036	918.6 (884.2–954.4)	901.9 (868.1–937.0)	927.8 (893.0–963.9)	947.1 (911.6–984.0)
2037	911.2 (877.1–946.7)	894.3 (860.8–929.2)	920.7 (886.2–956.6)	940.9 (905.7–977.6)
2038	903.7 (869.8–938.9)	886.6 (853.4–921.1)	913.5 (879.2–949.0)	934.5 (899.5–970.9)
2039	897.1 (863.5–932.0)	879.9 (846.9–914.2)	907.2 (873.2–942.5)	929.1 (894.3–965.3)
2040	889.8 (856.5–924.5)	872.5 (839.8–906.4)	900.2 (866.5–935.3)	923.0 (888.4–959.0)

	50–69			
Year	Reference	Scenario 1	Scenario 2	Scenario 3
2017	5689.8 (5575.9–5805.9)	5696.0 (5582.0–5812.3)	5696.0 (5582.0–5812.3)	5696.0 (5582.0–5812.3)
2018	5545.9 (5400.7–5695.0)	5540.3 (5395.2–5689.2)	5551.2 (5405.9–5700.4)	5555.0 (5409.6–5704.4)
2019	5474.1 (5332.1–5619.9)	5457.0 (5315.4–5602.3)	5478.5 (5336.4–5624.4)	5486.1 (5343.8–5632.2)

2020	5488.7 (5344.9–5636.3)	5460.0 (5317.0–5606.9)	5492.3 (5348.4–5640.0)	5503.7 (5359.6–5651.8)
2021	5427.7 (5283.9–5575.5)	5388.0 (5245.2–5534.6)	5430.5 (5286.6–5578.3)	5445.6 (5301.3–5593.8)
2022	5421.2 (5270.6–5576.2)	5370.2 (5220.9–5523.7)	5423.2 (5272.5–5578.2)	5442.0 (5290.8–5597.6)
2023	5430.2 (5278.5–5586.2)	5367.7 (5217.8–5522.0)	5431.4 (5279.6–5587.5)	5454.0 (5301.6–5610.8)
2024	5333.2 (5181.9–5489.0)	5270.2 (5120.6–5424.1)	5333.6 (5182.2–5489.4)	5359.6 (5207.4–5516.1)
2025	5241.0 (5073.0–5414.6)	5177.3 (5011.4–5348.8)	5240.6 (5072.6–5414.2)	5269.8 (5100.8–5444.3)
2026	5202.4 (5032.6–5377.9)	5137.5 (4969.9–5310.8)	5201.2 (5031.5–5376.7)	5233.8 (5063.0–5410.4)
2027	5072.9 (4908.3–5243.0)	5008.0 (4845.5–5175.9)	5071.0 (4906.5–5241.0)	5106.3 (4940.6–5277.5)
2028	4946.1 (4784.1–5113.5)	4881.2 (4721.4–5046.4)	4943.5 (4781.7–5110.8)	4981.4 (4818.3–5150.0)
2029	4869.9 (4707.3–5038.0)	4804.5 (4644.1–4970.4)	4866.6 (4704.2–5034.7)	4907.3 (4743.5–5076.8)
2030	4744.1 (4577.3–4917.1)	4678.9 (4514.3–4849.4)	4740.3 (4573.6–4913.1)	4783.2 (4615.0–4957.6)
2031	4630.8 (4467.3–4800.3)	4565.6 (4404.4–4732.7)	4626.4 (4463.1–4795.7)	4671.5 (4506.6–4842.5)
2032	4603.9 (4439.2–4774.7)	4537.7 (4375.3–4706.0)	4598.9 (4434.4–4769.5)	4646.9 (4480.7–4819.3)
2033	4546.3 (4375.3–4723.9)	4479.4 (4311.0–4654.4)	4540.6 (4369.9–4718.0)	4591.3 (4418.7–4770.7)
2034	4484.7 (4318.0–4657.8)	4417.3 (4253.1–4587.8)	4478.5 (4312.0–4651.3)	4531.6 (4363.2–4706.5)
2035	4485.3 (4318.9–4658.1)	4416.5 (4252.6–4586.6)	4478.4 (4312.3–4650.9)	4534.7 (4366.5–4709.4)
2036	4455.4 (4289.2–4628.1)	4385.6 (4222.0–4555.5)	4447.9 (4282.0–4620.3)	4506.9 (4338.8–4681.6)
2037	4394.5 (4227.3–4568.3)	4324.2 (4159.7–4495.2)	4386.4 (4219.5–4559.9)	4447.7 (4278.5–4623.6)
2038	4381.0 (4207.7–4561.5)	4309.6 (4139.1–4487.1)	4372.4 (4199.4–4552.5)	4436.5 (4261.0–4619.3)

2039	4329.7 (4158.3–4508.1)	4257.7 (4089.2–4433.1)	4320.5 (4149.5–4498.5)	4386.9 (4213.3–4567.7)
2040	4230.2 (4060.6–4406.9)	4158.5 (3991.8–4332.2)	4220.6 (4051.4–4396.9)	4288.5 (4116.5–4467.6)

	≥70			
Year	Reference	Scenario 1	Scenario 2	Scenario 3
2017	15482.5 (15045.4–15932.4)	15463.0 (15026.4–15912.3)	15463.0 (15026.4–15912.3)	15463.0 (15026.4–15912.3)
2018	15022.2 (14401.7–15669.5)	15089.1 (14465.9–15739.3)	15018.6 (14398.3–15665.7)	14993.8 (14374.5–15639.8)
2019	14721.0 (14073.9–15397.9)	14871.3 (14217.5–15555.0)	14732.6 (14085.0–15410.0)	14683.9 (14038.5–15359.1)
2020	14378.6 (13600.9–15200.8)	14608.4 (13818.3–15443.8)	14404.6 (13625.4–15228.2)	14333.3 (13558.0–15152.9)
2021	13843.9 (12958.2–14790.2)	14145.7 (13240.7–15112.6)	13883.1 (12994.8–14832.1)	13791.6 (12909.2–14734.3)
2022	13443.7 (12565.2–14383.6)	13815.4 (12912.6–14781.3)	13495.6 (12613.7–14439.1)	13384.5 (12509.9–14320.2)
2023	13111.6 (12244.1–14040.6)	13551.2 (12654.6–14511.4)	13175.7 (12303.9–14109.2)	13045.6 (12182.4–13969.9)
2024	12655.5 (11797.2–13576.2)	13103.0 (12214.4–14056.3)	12730.3 (11867.0–13656.5)	12583.8 (11730.4–13499.4)
2025	12262.3 (11436.5–13147.7)	12718.3 (11861.8–13636.7)	12347.4 (11515.9–13239.0)	12185.1 (11364.5–13065.0)
2026	12004.8 (11180.5–12889.8)	12473.2 (11616.8–13392.8)	12100.5 (11269.7–12992.5)	11921.7 (11103.2–12800.6)
2027	11688.3 (10875.5–12561.9)	12165.9 (11319.9–13075.2)	11793.5 (10973.4–12675.0)	11600.1 (10793.4–12467.1)
2028	11381.6 (10555.3–12272.7)	11867.6 (11006.0–12796.7)	11495.9 (10661.2–12395.8)	11288.7 (10469.1–12172.4)
2029	11165.5 (10290.7–12114.7)	11662.9 (10749.1–12654.3)	11289.1 (10404.6–12248.8)	11067.3 (10200.2–12008.1)

2030	10944.6 (10055.7–11912.2)	11452.4 (10522.2–12464.8)	11077.1 (10177.4–12056.3)	10841.5 (9961.0–11800.0)
2031	10684.4 (9793.1–11656.9)	11199.9 (10265.5–12219.2)	10824.8 (9921.8–11810.1)	10577.1 (9694.7–11539.8)
2032	10505.1 (9596.7–11499.6)	11031.4 (10077.4–12075.7)	10654.0 (9732.7–11662.6)	10393.1 (9494.3–11376.9)
2033	10340.4 (9436.2–11331.2)	10877.6 (9926.4–11919.8)	10497.7 (9579.7–11503.5)	10223.6 (9329.6–11203.2)
2034	10113.2 (9232.5–11077.8)	10657.4 (9729.3–11674.0)	10277.5 (9382.5–11257.9)	9992.6 (9122.5–10945.8)
2035	9906.8 (9044.1–10851.8)	10458.4 (9547.6–11456.0)	10078.1 (9200.5–11039.4)	9782.6 (8930.7–10715.7)
2036	9724.1 (8875.7–10653.6)	10283.7 (9386.5–11266.7)	9902.4 (9038.4–10848.9)	9596.1 (8758.9–10513.4)
2037	9479.6 (8637.3–10404.1)	10042.8 (9150.5–11022.2)	9663.3 (8804.6–10605.6)	9348.9 (8518.3–10260.6)
2038	9221.1 (8385.0–10140.5)	9786.2 (8898.8–10762.0)	9409.3 (8556.2–10347.6)	9088.2 (8264.2–9994.4)
2039	8989.0 (8158.3–9904.3)	9556.8 (8673.6–10529.9)	9181.9 (8333.4–10116.8)	8853.9 (8035.7–9755.5)
2040	8725.5 (7894.4–9644.1)	9293.0 (8407.8–10271.4)	8921.9 (8072.0–9861.2)	8588.9 (7770.8–9493.2)

	Women (1–B)			
	20–49			
Year	Reference	Scenario 1	Scenario 2	Scenario 3
2017	495.1 (481.6–508.9)	497.3 (483.7–511.2)	497.3 (483.7–511.2)	497.3 (483.7–511.2)
2018	471.7 (455.8–488.1)	474.1 (458.1–490.6)	474.6 (458.6–491.1)	474.8 (458.8–491.3)
2019	465.6 (448.7–483.1)	468.3 (451.3–485.9)	469.4 (452.3–487.0)	469.7 (452.7–487.4)

2020	454.6 (435.0–475.0)	457.5 (437.8–478.1)	459.1 (439.3–479.7)	459.6 (439.9–480.3)
2021	443.3 (424.6–462.9)	446.5 (427.6–466.2)	448.5 (429.5–468.3)	449.2 (430.2–469.1)
2022	446.2 (427.2–466.1)	449.7 (430.5–469.7)	452.3 (433.0–472.4)	453.2 (433.8–473.3)
2023	439.9 (420.6–460.1)	443.6 (424.2–464.0)	446.7 (427.1–467.2)	447.8 (428.1–468.3)
2024	434.6 (415.7–454.3)	438.0 (419.0–457.8)	442.1 (422.9–462.1)	443.3 (424.1–463.4)
2025	433.0 (414.0–452.8)	436.0 (417.0–456.0)	441.2 (421.9–461.4)	442.6 (423.3–462.9)
2026	426.8 (407.9–446.5)	429.5 (410.5–449.3)	435.7 (416.5–455.8)	437.3 (418.0–457.5)
2027	428.2 (407.1–450.4)	430.6 (409.4–452.9)	437.9 (416.3–460.6)	439.7 (418.0–462.5)
2028	429.3 (406.9–452.9)	431.4 (408.9–455.1)	439.8 (416.9–464.0)	441.8 (418.8–466.1)
2029	428.3 (405.3–452.6)	430.1 (407.0–454.5)	439.7 (416.0–464.6)	441.8 (418.1–466.8)
2030	429.0 (405.4–454.0)	430.5 (406.9–455.6)	441.2 (416.9–466.8)	443.5 (419.1–469.3)
2031	422.1 (398.9–446.6)	423.3 (400.0–447.9)	434.8 (410.9–460.1)	437.3 (413.3–462.6)
2032	416.7 (393.8–441.0)	417.6 (394.6–441.9)	430.1 (406.4–455.1)	432.7 (408.9–457.9)
2033	412.5 (389.9–436.5)	413.1 (390.5–437.1)	426.5 (403.1–451.3)	429.3 (405.7–454.2)
2034	404.3 (382.0–427.8)	404.6 (382.3–428.2)	418.7 (395.7–443.1)	421.6 (398.4–446.2)
2035	400.7 (378.6–424.2)	400.8 (378.6–424.2)	415.8 (392.8–440.2)	418.8 (395.7–443.4)
2036	393.9 (371.5–417.8)	393.7 (371.2–417.5)	409.5 (386.1–434.3)	412.6 (389.1–437.6)
2037	383.7 (361.1–407.7)	383.2 (360.6–407.1)	399.6 (376.1–424.5)	402.8 (379.1–428.0)
2038	377.8 (355.1–402.0)	377.0 (354.3–401.1)	394.1 (370.4–419.4)	397.5 (373.6–422.9)

2039	369.4 (346.6–393.6)	368.3 (345.7–392.5)	386.0 (362.3–411.4)	389.5 (365.5–415.0)
2040	363.2 (340.9–387.1)	362.0 (339.7–385.7)	380.3 (356.9–405.3)	383.9 (360.2–409.1)

	50–69			
Year	Reference	Scenario 1	Scenario 2	Scenario 3
2017	2552.8 (2501.8–2604.9)	2559.0 (2507.9–2611.2)	2559.0 (2507.9–2611.2)	2559.0 (2507.9–2611.2)
2018	2477.8 (2413.1–2544.2)	2478.7 (2413.9–2545.2)	2484.7 (2419.8–2551.3)	2486.8 (2421.9–2553.5)
2019	2418.8 (2349.3–2490.4)	2414.7 (2345.3–2486.1)	2426.4 (2356.7–2498.2)	2430.6 (2360.8–2502.5)
2020	2390.1 (2300.1–2483.6)	2381.1 (2291.4–2474.3)	2398.5 (2308.1–2492.3)	2404.6 (2314.1–2498.7)
2021	2317.1 (2221.6–2416.7)	2303.6 (2208.6–2402.6)	2326.0 (2230.1–2426.0)	2334.0 (2237.8–2434.3)
2022	2263.3 (2168.9–2361.8)	2245.5 (2151.8–2343.2)	2272.9 (2178.0–2371.8)	2282.6 (2187.4–2382.0)
2023	2226.5 (2128.9–2328.5)	2204.4 (2107.8–2305.4)	2236.7 (2138.7–2339.2)	2248.2 (2149.7–2351.2)
2024	2171.8 (2076.4–2271.7)	2148.0 (2053.6–2246.8)	2182.5 (2086.6–2282.9)	2195.7 (2099.2–2296.6)
2025	2123.6 (2030.3–2221.3)	2098.2 (2006.0–2194.6)	2134.9 (2041.0–2233.0)	2149.5 (2055.1–2248.4)
2026	2096.6 (2004.4–2193.0)	2069.3 (1978.3–2164.5)	2108.4 (2015.7–2205.4)	2124.7 (2031.3–2222.5)
2027	2055.9 (1964.3–2151.9)	2027.1 (1936.7–2121.7)	2068.3 (1976.1–2164.8)	2086.1 (1993.1–2183.4)
2028	2021.1 (1926.6–2120.3)	1990.7 (1897.6–2088.3)	2034.0 (1938.9–2133.8)	2053.2 (1957.2–2154.0)
2029	2000.7 (1905.1–2101.1)	1968.5 (1874.5–2067.3)	2014.2 (1917.9–2115.3)	2035.0 (1937.7–2137.1)

2030	1970.0 (1873.3–2071.7)	1936.3 (1841.2–2036.2)	1984.0 (1886.6–2086.4)	2006.1 (1907.7–2109.7)
2031	1943.9 (1844.8–2048.4)	1908.7 (1811.4–2011.2)	1958.4 (1858.6–2063.6)	1982.0 (1881.0–2088.5)
2032	1921.7 (1822.9–2025.9)	1884.9 (1788.0–1987.1)	1936.7 (1837.1–2041.8)	1961.8 (1860.9–2068.1)
2033	1894.7 (1796.8–1997.9)	1856.4 (1760.5–1957.6)	1910.1 (1811.4–2014.2)	1936.5 (1836.4–2042.0)
2034	1864.6 (1767.8–1966.8)	1825.1 (1730.3–1925.1)	1880.5 (1782.9–1983.5)	1908.1 (1809.0–2012.6)
2035	1838.6 (1743.3–1939.2)	1797.8 (1704.6–1896.1)	1855.0 (1758.8–1956.4)	1883.8 (1786.1–1986.8)
2036	1804.9 (1711.0–1903.8)	1762.9 (1671.3–1859.6)	1821.5 (1726.8–1921.4)	1851.4 (1755.1–1952.9)
2037	1771.5 (1679.1–1868.9)	1728.5 (1638.4–1823.6)	1788.5 (1695.3–1886.8)	1819.4 (1724.5–1919.4)
2038	1738.8 (1647.2–1835.5)	1694.9 (1605.6–1789.2)	1756.1 (1663.6–1853.8)	1788.0 (1693.8–1887.4)
2039	1701.9 (1611.0–1797.9)	1657.2 (1568.7–1750.7)	1719.4 (1627.6–1816.4)	1752.1 (1658.5–1850.9)
2040	1667.8 (1578.0–1762.6)	1622.3 (1535.0–1714.6)	1685.6 (1594.9–1781.5)	1719.1 (1626.6–1816.9)

	≥70			
Year	Reference	Scenario 1	Scenario 2	Scenario 3
2017	11358.6 (11074.4–11650.2)	11366.0 (11081.5–11657.7)	11366.0 (11081.5–11657.7)	11366.0 (11081.5–11657.7)
2018	11110.7 (10717.0–11518.8)	11098.4 (10705.1–11506.1)	11115.4 (10721.6–11523.7)	11121.4 (10727.4–11529.9)
2019	10851.5 (10371.4–11353.8)	10820.5 (10341.8–11321.4)	10853.7 (10373.5–11356.1)	10865.5 (10384.8–11368.4)
2020	10413.8 (9809.1–11055.7)	10365.9 (9764.0–11004.8)	10413.6 (9809.0–11055.5)	10430.6 (9824.9–11073.5)

2021	10061.4 (9443.2–10720.0)	9997.5 (9383.3–10652.0)	10059.0 (9441.0–10717.5)	10080.8 (9461.4–10740.8)
2022	9824.2 (9200.0–10490.7)	9744.8 (9125.6–10405.9)	9819.8 (9195.9–10486.0)	9846.4 (9220.8–10514.4)
2023	9575.8 (8931.4–10266.6)	9481.7 (8843.7–10165.8)	9569.4 (8925.4–10259.7)	9600.5 (8954.5–10293.1)
2024	9418.4 (8780.7–10102.4)	9314.7 (8684.0–9991.2)	9410.0 (8772.9–10093.5)	9445.7 (8806.2–10131.8)
2025	9086.6 (8463.5–9755.6)	8975.8 (8360.3–9636.6)	9076.6 (8454.2–9744.8)	9116.0 (8490.8–9787.1)
2026	8761.2 (8158.2–9408.8)	8644.0 (8049.0–9282.9)	8749.6 (8147.4–9396.3)	8792.3 (8187.2–9442.2)
2027	8530.2 (7936.8–9167.9)	8405.9 (7821.1–9034.4)	8517.0 (7924.5–9153.8)	8563.2 (7967.5–9203.4)
2028	8245.2 (7656.9–8878.7)	8115.3 (7536.2–8738.9)	8230.7 (7643.4–8863.1)	8279.8 (7689.0–8916.0)
2029	8170.8 (7558.7–8832.4)	8032.4 (7430.7–8682.8)	8154.6 (7543.7–8814.9)	8207.7 (7592.8–8872.3)
2030	8112.8 (7479.9–8799.3)	7965.8 (7344.4–8639.8)	8095.0 (7463.5–8779.9)	8152.1 (7516.2–8841.9)
2031	7950.3 (7310.4–8646.2)	7796.8 (7169.3–8479.3)	7931.0 (7292.7–8625.2)	7991.3 (7348.1–8690.8)
2032	7879.5 (7227.9–8589.8)	7718.1 (7079.9–8413.9)	7858.7 (7208.9–8567.1)	7922.7 (7267.6–8636.9)
2033	7699.2 (7055.4–8401.7)	7532.5 (6902.6–8219.7)	7677.2 (7035.3–8377.7)	7743.9 (7096.4–8450.5)
2034	7541.9 (6897.8–8246.1)	7369.7 (6740.3–8057.8)	7518.7 (6876.6–8220.8)	7588.2 (6940.1–8296.7)
2035	7503.6 (6857.0–8211.1)	7323.5 (6692.5–8014.0)	7478.9 (6834.5–8184.0)	7552.1 (6901.4–8264.1)
2036	7404.9 (6766.6–8103.5)	7218.5 (6596.2–7899.5)	7378.9 (6742.8–8075.1)	7455.2 (6812.5–8158.5)
2037	7347.3 (6714.1–8040.3)	7153.7 (6537.2–7828.4)	7319.9 (6689.1–8010.3)	7399.6 (6761.9–8097.4)
2038	7230.7 (6601.1–7920.3)	7031.7 (6419.5–7702.4)	7202.2 (6575.1–7889.1)	7284.5 (6650.2–7979.2)
2039	7026.8 (6401.9–7712.7)	6825.2 (6218.3–7491.5)	6997.5 (6375.2–7680.6)	7081.3 (6451.6–7772.5)

2040	6858.5 (6240.5–7537.6)	6653.7 (6054.2–7312.6)	6828.4 (6213.2–7504.6)	6913.9 (6291.0–7598.6)

	Both sexes combined (2–C)			
	20–49			
Year	Reference	Scenario 1	Scenario 2	Scenario 3
2017	760.0 (742.2–778.1)	762.2 (744.5–780.4)	762.2 (744.5–780.4)	762.2 (744.5–780.4)
2018	747.5 (726.9–768.7)	748.9 (728.2–770.1)	750.4 (729.7–771.7)	751.0 (730.2–772.3)
2019	750.5 (728.4–773.2)	751.1 (729.0–773.8)	754.1 (731.9–777.0)	755.2 (733.0–778.1)
2020	744.9 (722.0–768.6)	744.7 (721.8–768.3)	749.2 (726.2–773.0)	750.8 (727.7–774.7)
2021	738.9 (716.5–762.0)	737.8 (715.4–760.9)	743.8 (721.2–767.1)	745.9 (723.3–769.3)
2022	745.4 (721.9–769.8)	743.5 (720.0–767.8)	751.1 (727.3–775.6)	753.8 (729.9–778.3)
2023	739.4 (715.8–763.8)	736.7 (713.1–761.0)	745.7 (721.8–770.3)	748.9 (724.9–773.6)
2024	735.0 (710.6–760.2)	732.0 (707.7–757.1)	741.9 (717.3–767.3)	745.6 (720.9–771.1)
2025	736.5 (710.6–763.4)	733.2 (707.4–759.9)	744.1 (717.9–771.2)	748.4 (722.0–775.6)
2026	724.8 (699.3–751.2)	721.2 (695.8–747.5)	732.9 (707.1–759.6)	737.6 (711.6–764.5)
2027	714.0 (687.6–741.3)	710.1 (683.9–737.4)	722.6 (695.9–750.3)	727.8 (700.9–755.7)
2028	708.1 (681.9–735.3)	704.0 (677.9–731.0)	717.3 (690.7–744.8)	722.9 (696.2–750.7)
2029	691.8 (665.9–718.7)	687.5 (661.8–714.2)	701.4 (675.1–728.7)	707.4 (681.0–734.9)

2030	681.1 (655.5–707.8)	676.6 (651.1–703.1)	691.2 (665.2–718.3)	697.7 (671.4–725.0)
2031	675.1 (649.1–702.1)	670.4 (644.5–697.2)	685.7 (659.3–713.2)	692.6 (665.9–720.3)
2032	662.4 (635.9–690.1)	657.5 (631.1–684.9)	673.4 (646.4–701.5)	680.7 (653.4–709.1)
2033	656.7 (630.4–684.0)	651.5 (625.4–678.7)	668.2 (641.5–696.0)	675.9 (648.8–704.0)
2034	654.7 (628.2–682.4)	649.3 (623.0–676.7)	666.8 (639.8–695.0)	675.0 (647.6–703.5)
2035	647.3 (620.7–675.1)	641.7 (615.3–669.2)	659.9 (632.7–688.2)	668.4 (640.9–697.1)
2036	646.5 (620.1–674.0)	640.6 (614.4–667.9)	659.6 (632.7–687.7)	668.6 (641.3–697.1)
2037	646.2 (619.7–673.8)	640.1 (613.9–667.4)	659.9 (632.9–688.1)	669.4 (642.0–698.0)
2038	639.8 (613.4–667.3)	633.4 (607.3–660.7)	653.9 (626.9–682.1)	663.8 (636.4–692.4)
2039	637.8 (610.7–666.0)	631.2 (604.4–659.2)	652.5 (624.7–681.4)	662.8 (634.6–692.2)
2040	633.7 (606.6–662.0)	626.9 (600.1–654.9)	648.9 (621.2–677.9)	659.7 (631.5–689.1)

	50–69			
Year	Reference	Scenario 1	Scenario 2	Scenario 3
2017	4210.7 (4139.1–4283.7)	4218.9 (4147.0–4291.9)	4218.9 (4147.0–4291.9)	4218.9 (4147.0–4291.9)
2018	4060.3 (3922.0–4203.4)	4057.3 (3919.2–4200.3)	4068.2 (3929.7–4211.6)	4072.0 (3933.4–4215.6)
2019	4003.3 (3858.0–4154.0)	3989.7 (3844.9–4139.9)	4011.2 (3865.6–4162.2)	4018.8 (3872.9–4170.1)
2020	4037.5 (3887.8–4193.1)	4013.1 (3864.3–4167.7)	4045.5 (3895.5–4201.4)	4057.1 (3906.6–4213.3)

2021	3885.6 (3736.9–4040.3)	3851.8 (3704.4–4005.1)	3893.4 (3744.4–4048.3)	3908.2 (3758.6–4063.7)
2022	3821.1 (3669.0–3979.4)	3777.8 (3627.4–3934.3)	3828.8 (3676.4–3987.5)	3847.0 (3693.9–4006.4)
2023	3860.0 (3699.6–4027.5)	3806.1 (3647.9–3971.2)	3867.9 (3707.1–4035.7)	3889.9 (3728.2–4058.7)
2024	3736.5 (3580.5–3899.3)	3681.8 (3528.0–3842.2)	3744.2 (3587.8–3907.3)	3769.1 (3611.7–3933.3)
2025	3665.7 (3510.4–3828.0)	3609.5 (3456.6–3769.3)	3673.3 (3517.6–3835.9)	3701.3 (3544.4–3865.1)
2026	3698.0 (3537.0–3866.3)	3638.8 (3480.4–3804.4)	3705.7 (3544.4–3874.4)	3737.5 (3574.8–3907.6)
2027	3592.6 (3435.2–3757.2)	3532.6 (3377.9–3694.4)	3600.2 (3442.5–3765.1)	3634.4 (3475.2–3800.9)
2028	3517.4 (3362.8–3679.1)	3456.3 (3304.4–3615.2)	3524.9 (3370.0–3687.0)	3561.8 (3405.3–3725.6)
2029	3542.7 (3384.0–3708.8)	3478.7 (3322.9–3641.9)	3550.3 (3391.3–3716.8)	3590.9 (3430.0–3759.2)
2030	3453.1 (3297.4–3616.0)	3388.4 (3235.7–3548.3)	3460.5 (3304.6–3623.8)	3503.4 (3345.5–3668.7)
2031	3375.8 (3223.7–3535.0)	3310.2 (3161.1–3466.4)	3383.1 (3230.7–3542.7)	3428.3 (3273.9–3590.0)
2032	3394.0 (3238.9–3556.5)	3325.8 (3173.8–3485.0)	3401.4 (3246.0–3564.3)	3450.1 (3292.4–3615.3)
2033	3317.9 (3165.5–3477.7)	3249.0 (3099.8–3405.5)	3325.3 (3172.5–3485.4)	3376.1 (3221.0–3538.6)
2034	3240.3 (3091.7–3396.1)	3170.8 (3025.4–3323.2)	3247.6 (3098.6–3403.6)	3300.3 (3148.9–3458.9)
2035	3251.7 (3100.9–3409.9)	3179.8 (3032.3–3334.5)	3259.0 (3107.8–3417.6)	3315.0 (3161.3–3476.3)
2036	3187.3 (3038.8–3343.0)	3114.6 (2969.5–3266.7)	3194.5 (3045.7–3350.5)	3252.5 (3101.0–3411.4)
2037	3110.7 (2966.1–3262.4)	3037.7 (2896.4–3185.8)	3117.8 (2972.8–3269.9)	3177.4 (3029.7–3332.4)
2038	3115.7 (2969.4–3269.1)	3040.4 (2897.7–3190.2)	3122.8 (2976.3–3276.6)	3185.6 (3036.0–3342.5)
2039	3061.0 (2916.9–3212.2)	2985.0 (2844.5–3132.4)	3068.1 (2923.7–3219.6)	3132.7 (2985.2–3287.4)

2040	2986.6 (2846.2–3133.9)	2910.4 (2773.6–3054.0)	2993.6 (2852.8–3141.3)	3059.5 (2915.6–3210.4)

	≥70			
Year	Reference	Scenario 1	Scenario 2	Scenario 3
2017	13117.0 (12768.0–13475.5)	13118.3 (12769.2–13476.8)	13118.3 (12769.2–13476.8)	13118.3 (12769.2–13476.8)
2018	12735.3 (12282.7–13204.6)	12731.8 (12279.3–13201.0)	12735.8 (12283.1–13205.1)	12737.2 (12284.5–13206.6)
2019	12621.2 (12117.4–13145.9)	12613.1 (12109.6–13137.5)	12620.9 (12117.1–13145.6)	12623.6 (12119.8–13148.5)
2020	12316.2 (11665.3–13003.3)	12303.7 (11653.5–12990.2)	12315.2 (11664.4–13002.3)	12319.2 (11668.2–13006.5)
2021	11893.2 (11192.1–12638.3)	11876.8 (11176.6–12620.8)	11891.5 (11190.5–12636.5)	11896.7 (11195.4–12642.0)
2022	11691.3 (10982.5–12445.9)	11670.8 (10963.2–12424.1)	11688.9 (10980.3–12443.4)	11695.3 (10986.3–12450.2)
2023	11380.3 (10646.5–12164.7)	11356.2 (10623.9–12138.9)	11377.3 (10643.7–12161.5)	11384.8 (10650.6–12169.5)
2024	10991.8 (10274.2–11759.5)	10966.6 (10250.7–11732.6)	10988.2 (10270.9–11755.7)	10996.7 (10278.7–11764.7)
2025	10777.3 (10077.0–11526.2)	10750.8 (10052.2–11497.9)	10773.2 (10073.2–11521.8)	10782.6 (10082.0–11531.9)
2026	10516.5 (9833.2–11247.3)	10488.9 (9807.3–11217.8)	10511.8 (9828.8–11242.3)	10522.2 (9838.5–11253.4)
2027	10206.7 (9539.1–10921.1)	10178.2 (9512.4–10890.6)	10201.6 (9534.3–10915.6)	10212.8 (9544.7–10927.6)
2028	10031.6 (9350.9–10761.9)	10001.8 (9323.2–10729.9)	10025.9 (9345.6–10755.8)	10038.0 (9356.9–10768.7)
2029	9840.9 (9156.2–10576.8)	9810.0 (9127.5–10543.6)	9834.7 (9150.5–10570.2)	9847.7 (9162.5–10584.0)
2030	9578.1 (8888.8–10321.0)	9546.5 (8859.4–10286.9)	9571.6 (8882.7–10313.9)	9585.2 (8895.4–10328.6)

2031	9412.4 (8702.1–10180.6)	9379.7 (8671.8–10145.3)	9405.3 (8695.6–10173.1)	9419.8 (8708.9–10188.7)
2032	9241.0 (8528.4–10013.2)	9207.4 (8497.3–9976.7)	9233.6 (8521.5–10005.1)	9248.8 (8535.5–10021.6)
2033	9034.4 (8327.2–9801.6)	9000.0 (8295.4–9764.3)	9026.6 (8320.0–9793.2)	9042.4 (8334.5–9810.3)
2034	8888.0 (8182.8–9654.0)	8852.6 (8150.2–9615.6)	8879.8 (8175.2–9645.1)	8896.3 (8190.4–9663.0)
2035	8733.5 (8040.2–9486.6)	8697.3 (8006.8–9447.2)	8724.9 (8032.3–9477.2)	8742.1 (8048.1–9495.9)
2036	8541.5 (7863.8–9277.5)	8504.6 (7829.9–9237.4)	8532.5 (7855.6–9267.8)	8550.3 (7872.0–9287.0)
2037	8382.7 (7717.5–9105.2)	8345.1 (7682.9–9064.4)	8373.4 (7709.0–9095.2)	8391.8 (7725.9–9115.1)
2038	8214.3 (7558.8–8926.6)	8176.1 (7523.6–8885.1)	8204.7 (7550.0–8916.2)	8223.6 (7567.3–8936.7)
2039	8012.0 (7365.1–8715.7)	7973.4 (7329.6–8673.7)	8002.2 (7356.1–8705.0)	8021.5 (7373.8–8726.0)
2040	7829.4 (7190.0–8525.6)	7790.3 (7154.2–8483.0)	7819.3 (7180.8–8514.6)	7839.0 (7198.9–8536.1)

	All age (1–D)			
	Men			
Year	Reference	Scenario 1	Scenario 2	Scenario 3
2017	4596.6 (4492.7–4702.8)	4604.9 (4500.9–4711.4)	4604.9 (4500.9–4711.4)	4604.9 (4500.9–4711.4)
2018	4595.5 (4491.7–4701.7)	4594.6 (4490.8–4700.7)	4604.5 (4500.5–4710.9)	4608.0 (4503.9–4714.4)
2019	4594.3 (4490.5–4700.5)	4583.6 (4480.1–4689.5)	4603.4 (4499.4–4709.7)	4610.4 (4506.2–4716.9)
2020	4593.4 (4489.6–4699.5)	4572.9 (4469.6–4678.6)	4602.5 (4498.5–4708.8)	4613.0 (4508.8–4719.6)

2021	4592.4 (4488.6–4698.5)	4562.1 (4459.1–4667.5)	4601.5 (4497.6–4707.9)	4615.5 (4511.3–4722.2)
2022	4591.4 (4487.7–4697.5)	4551.4 (4448.6–4656.6)	4600.6 (4496.7–4706.9)	4618.1 (4513.8–4724.8)
2023	4590.4 (4486.7–4696.4)	4540.7 (4438.1–4645.6)	4599.7 (4495.8–4706.0)	4620.7 (4516.3–4727.4)
2024	4589.4 (4485.7–4695.4)	4538.3 (4435.8–4643.1)	4598.7 (4494.9–4705.0)	4623.2 (4518.8–4730.1)
2025	4588.4 (4484.7–4694.4)	4535.8 (4433.4–4640.6)	4597.8 (4494.0–4704.1)	4625.8 (4521.3–4732.7)
2026	4587.4 (4483.8–4693.4)	4533.4 (4431.0–4638.1)	4596.9 (4493.0–4703.1)	4628.4 (4523.8–4735.3)
2027	4586.4 (4482.8–4692.3)	4531.0 (4428.6–4635.7)	4595.9 (4492.1–4702.1)	4631.0 (4526.4–4738.0)
2028	4585.4 (4481.8–4691.3)	4528.5 (4426.2–4633.2)	4595.0 (4491.2–4701.2)	4633.5 (4528.9–4740.6)
2029	4584.4 (4480.8–4690.3)	4526.1 (4423.9–4630.7)	4594.1 (4490.3–4700.2)	4636.1 (4531.4–4743.2)
2030	4583.4 (4479.9–4689.3)	4523.7 (4421.5–4628.2)	4593.1 (4489.4–4699.3)	4638.7 (4533.9–4745.9)
2031	4582.4 (4478.9–4688.3)	4521.2 (4419.1–4625.7)	4592.2 (4488.5–4698.3)	4641.3 (4536.4–4748.5)
2032	4581.4 (4477.9–4687.2)	4518.8 (4416.7–4623.2)	4591.3 (4487.6–4697.4)	4643.8 (4539.0–4751.2)
2033	4580.4 (4476.9–4686.2)	4516.4 (4414.4–4620.7)	4590.3 (4486.7–4696.4)	4646.4 (4541.5–4753.8)
2034	4579.4 (4476.0–4685.2)	4514.0 (4412.0–4618.3)	4589.4 (4485.8–4695.5)	4649.0 (4544.0–4756.4)
2035	4578.4 (4475.0–4684.2)	4511.5 (4409.6–4615.8)	4588.5 (4484.8–4694.5)	4651.6 (4546.5–4759.1)
2036	4577.4 (4474.0–4683.2)	4509.1 (4407.3–4613.3)	4587.5 (4483.9–4693.5)	4654.2 (4549.1–4761.7)
2037	4576.4 (4473.0–4682.1)	4506.7 (4404.9–4610.8)	4586.6 (4483.0–4692.6)	4656.8 (4551.6–4764.4)
2038	4575.4 (4472.1–4681.1)	4504.3 (4402.5–4608.3)	4585.7 (4482.1–4691.6)	4659.4 (4554.1–4767.0)
2039	4574.4 (4471.1–4680.1)	4501.8 (4400.2–4605.9)	4584.7 (4481.2–4690.7)	4662.0 (4556.7–4769.7)

2040	4573.4 (4470.1–4679.1)	4499.4 (4397.8–4603.4)	4583.8 (4480.3–4689.7)	4664.5 (4559.2–4772.3)

	Women			
Year	Reference	Scenario 1	Scenario 2	Scenario 3
2017	3263.5 (3191.2–3337.4)	3265.8 (3193.5–3339.8)	3265.8 (3193.5–3339.8)	3265.8 (3193.5–3339.8)
2018	3374.1 (3248.4–3504.8)	3375.4 (3249.6–3506.1)	3377.2 (3251.3–3507.9)	3377.8 (3251.9–3508.6)
2019	3350.4 (3220.3–3485.8)	3350.5 (3220.4–3486.0)	3354.0 (3223.7–3489.6)	3355.3 (3224.9–3490.9)
2020	3360.6 (3219.6–3507.8)	3359.6 (3218.7–3506.7)	3364.9 (3223.7–3512.2)	3366.7 (3225.5–3514.2)
2021	3374.3 (3227.2–3528.1)	3372.1 (3225.1–3525.8)	3379.2 (3231.8–3533.2)	3381.6 (3234.2–3535.8)
2022	3262.9 (3118.5–3413.9)	3259.6 (3115.4–3410.5)	3268.1 (3123.6–3419.4)	3271.1 (3126.4–3422.5)
2023	3290.7 (3144.1–3444.1)	3286.3 (3140.0–3439.5)	3296.6 (3149.8–3450.3)	3300.2 (3153.2–3454.1)
2024	3203.1 (3057.5–3355.7)	3198.0 (3052.7–3350.3)	3209.5 (3063.6–3362.3)	3213.6 (3067.5–3366.6)
2025	3246.3 (3090.7–3409.6)	3240.2 (3085.0–3403.3)	3253.2 (3097.3–3417.0)	3258.0 (3101.9–3422.0)
2026	3242.8 (3085.9–3407.7)	3236.0 (3079.4–3400.5)	3250.4 (3093.1–3415.6)	3255.8 (3098.2–3421.3)
2027	3248.9 (3089.7–3416.4)	3241.2 (3082.3–3408.2)	3257.1 (3097.5–3424.9)	3263.1 (3103.1–3431.2)
2028	3271.0 (3110.8–3439.5)	3262.3 (3102.5–3430.4)	3279.8 (3119.1–3448.7)	3286.4 (3125.4–3455.7)
2029	3209.3 (3051.4–3375.3)	3199.9 (3042.5–3365.5)	3218.4 (3060.1–3384.9)	3225.5 (3066.9–3392.4)
2030	3206.3 (3048.5–3372.3)	3196.1 (3038.8–3361.6)	3216.1 (3057.7–3382.6)	3223.7 (3065.1–3390.6)

2031	3140.2 (2983.3–3305.3)	3129.4 (2973.0–3293.9)	3150.3 (2992.9–3315.9)	3158.4 (3000.6–3324.4)
2032	3138.0 (2980.2–3304.2)	3126.4 (2969.1–3292.0)	3148.7 (2990.3–3315.5)	3157.4 (2998.5–3324.6)
2033	3135.4 (2977.6–3301.5)	3122.9 (2965.7–3288.4)	3146.6 (2988.2–3313.3)	3155.8 (2997.0–3323.1)
2034	3140.6 (2981.4–3308.3)	3127.3 (2968.8–3294.3)	3152.4 (2992.6–3320.7)	3162.2 (3002.0–3331.1)
2035	3165.0 (3004.6–3333.9)	3150.7 (2991.1–3318.9)	3177.4 (3016.4–3347.0)	3187.9 (3026.4–3358.1)
2036	3136.5 (2977.3–3304.1)	3121.5 (2963.1–3288.4)	3149.3 (2989.6–3317.7)	3160.3 (3000.0–3329.3)
2037	3126.3 (2967.7–3293.4)	3110.6 (2952.8–3276.9)	3139.7 (2980.4–3307.6)	3151.3 (2991.4–3319.7)
2038	3076.6 (2919.3–3242.5)	3060.4 (2903.8–3225.3)	3090.4 (2932.3–3257.0)	3102.3 (2943.6–3269.5)
2039	3052.9 (2896.7–3217.4)	3035.9 (2880.7–3199.6)	3067.1 (2910.2–3232.4)	3079.5 (2922.0–3245.5)
2040	3040.0 (2884.5–3203.8)	3022.3 (2867.7–3185.2)	3054.6 (2898.4–3219.3)	3067.6 (2910.7–3232.9)

	Both sexes combined			
Year	Reference	Scenario 1	Scenario 2	Scenario 3
2017	4039.6 (3968.1–4112.4)	4047.0 (3975.4–4119.9)	4047.0 (3975.4–4119.9)	4047.0 (3975.4–4119.9)
2018	4004.6 (3883.0–4129.9)	4005.4 (3883.9–4130.8)	4012.9 (3891.2–4138.5)	4015.6 (3893.7–4141.3)
2019	4011.2 (3890.3–4135.8)	4005.6 (3884.8–4130.1)	4020.6 (3899.4–4145.6)	4025.9 (3904.6–4151.0)
2020	3976.4 (3851.4–4105.5)	3964.5 (3839.8–4093.1)	3986.8 (3861.4–4116.2)	3994.7 (3869.1–4124.4)
2021	3939.9 (3815.8–4068.0)	3921.7 (3798.2–4049.2)	3951.2 (3826.7–4079.6)	3961.6 (3836.9–4090.4)

2022	3958.9 (3833.0–4088.9)	3934.2 (3809.1–4063.5)	3971.2 (3844.9–4101.7)	3984.4 (3857.6–4115.3)
2023	3994.0 (3867.2–4124.9)	3962.7 (3836.9–4092.6)	4007.5 (3880.3–4138.8)	4023.4 (3895.7–4155.3)
2024	4016.0 (3886.0–4150.4)	3982.7 (3853.8–4116.0)	4030.6 (3900.1–4165.5)	4049.3 (3918.2–4184.8)
2025	4014.9 (3883.2–4151.0)	3979.7 (3849.2–4114.7)	4030.5 (3898.3–4167.2)	4051.8 (3919.0–4189.2)
2026	3985.2 (3854.8–4119.9)	3948.4 (3819.3–4081.9)	4001.7 (3870.9–4137.0)	4025.6 (3893.9–4161.7)
2027	3951.9 (3822.0–4086.1)	3913.6 (3785.1–4046.6)	3969.3 (3838.9–4104.2)	3995.6 (3864.3–4131.4)
2028	3945.0 (3813.6–4080.9)	3905.0 (3774.9–4039.5)	3963.4 (3831.4–4100.0)	3992.3 (3859.3–4129.9)
2029	3967.1 (3834.9–4103.9)	3925.1 (3794.2–4060.4)	3986.7 (3853.8–4124.1)	4018.4 (3884.4–4157.0)
2030	3998.4 (3864.9–4136.5)	3954.2 (3822.2–4090.8)	4019.2 (3885.0–4158.0)	4053.8 (3918.5–4193.8)
2031	4012.6 (3876.9–4153.0)	3966.3 (3832.2–4105.2)	4034.4 (3897.9–4175.6)	4071.9 (3934.2–4214.4)
2032	3996.7 (3861.1–4137.2)	3948.8 (3814.8–4087.6)	4019.5 (3883.1–4160.8)	4059.5 (3921.7–4202.2)
2033	3964.3 (3829.9–4103.4)	3914.9 (3782.2–4052.3)	3987.9 (3852.7–4127.8)	4030.3 (3893.7–4171.7)
2034	3942.5 (3807.5–4082.2)	3891.6 (3758.3–4029.5)	3967.0 (3831.2–4107.6)	4011.8 (3874.4–4154.0)
2035	3948.3 (3812.1–4089.3)	3895.5 (3761.2–4034.6)	3973.8 (3836.8–4115.8)	4021.4 (3882.7–4165.0)
2036	3975.6 (3838.8–4117.3)	3920.6 (3785.7–4060.3)	4002.3 (3864.6–4145.0)	4052.9 (3913.4–4197.3)
2037	4000.5 (3862.0–4144.0)	3943.3 (3806.8–4084.8)	4028.5 (3889.0–4173.0)	4082.1 (3940.7–4228.5)
2038	4001.3 (3861.5–4146.2)	3942.3 (3804.6–4085.1)	4030.3 (3889.5–4176.3)	4086.6 (3943.8–4234.6)
2039	3977.2 (3838.3–4121.0)	3916.7 (3780.0–4058.4)	4007.0 (3867.2–4152.0)	4065.7 (3923.8–4212.8)

2040	3948.4 (3810.2–4091.6)	3886.5 (3750.5–4027.5)	3979.1 (3839.8–4123.4)	4040.0 (3898.6–4186.5)

	Chronic kidney disease (2)			
	Men (2–A)			
	20–49			
Year	Reference	Scenario 1	Scenario 2	Scenario 3
2017	118.5 (116.7–120.3)	118.6 (116.8–120.4)	118.6 (116.8–120.4)	118.6 (116.8–120.4)
2018	115.0 (112.2–117.9)	115.0 (112.2–117.9)	115.1 (112.3–118.0)	115.2 (112.4–118.1)
2019	113.6 (110.5–116.9)	113.4 (110.3–116.7)	113.8 (110.6–117.0)	113.9 (110.7–117.1)
2020	113.7 (110.4–117.1)	113.4 (110.0–116.8)	113.9 (110.5–117.3)	114.0 (110.7–117.5)
2021	112.5 (109.1–116.1)	112.1 (108.6–115.7)	112.7 (109.2–116.3)	113.0 (109.5–116.6)
2022	111.6 (108.0–115.4)	111.1 (107.4–114.8)	111.8 (108.2–115.6)	112.1 (108.5–115.9)
2023	111.2 (107.5–114.9)	110.4 (106.8–114.2)	111.4 (107.8–115.1)	111.7 (108.1–115.5)
2024	110.9 (107.3–114.7)	110.2 (106.6–114.0)	111.2 (107.5–115.0)	111.6 (107.9–115.4)
2025	111.0 (107.3–114.8)	110.2 (106.6–114.0)	111.2 (107.6–115.0)	111.7 (108.0–115.5)
2026	111.6 (107.6–115.7)	110.8 (106.9–114.9)	111.9 (107.9–116.0)	112.4 (108.4–116.5)
2027	113.3 (109.0–117.7)	112.5 (108.3–116.9)	113.6 (109.4–118.0)	114.2 (109.9–118.6)
2028	111.7 (107.4–116.1)	110.9 (106.6–115.3)	112.0 (107.7–116.5)	112.6 (108.3–117.1)

2029	110.5 (106.2–114.9)	109.7 (105.4–114.1)	110.8 (106.5–115.3)	111.5 (107.2–116.0)
2030	110.8 (106.3–115.5)	110.0 (105.5–114.6)	111.2 (106.7–115.8)	111.9 (107.4–116.6)
2031	109.9 (105.4–114.6)	109.1 (104.6–113.7)	110.3 (105.8–115.0)	111.1 (106.5–115.8)
2032	108.8 (104.3–113.4)	107.9 (103.5–112.6)	109.2 (104.7–113.9)	110.0 (105.5–114.7)
2033	107.4 (103.0–112.0)	106.5 (102.1–111.1)	107.8 (103.4–112.5)	108.7 (104.2–113.4)
2034	106.0 (101.6–110.6)	105.1 (100.7–109.7)	106.4 (102.0–111.1)	107.3 (102.9–112.0)
2035	103.4 (99.0–108.1)	102.6 (98.1–107.2)	103.9 (99.4–108.5)	104.8 (100.3–109.5)
2036	102.2 (97.7–106.8)	101.3 (96.9–106.0)	102.6 (98.1–107.3)	103.6 (99.1–108.4)
2037	102.1 (97.6–106.8)	101.2 (96.8–105.9)	102.6 (98.1–107.3)	103.6 (99.0–108.4)
2038	100.8 (96.3–105.5)	99.9 (95.4–104.6)	101.3 (96.7–106.0)	102.3 (97.8–107.1)
2039	99.8 (95.2–104.6)	98.9 (94.4–103.7)	100.3 (95.7–105.1)	101.4 (96.8–106.3)
2040	98.8 (94.3–103.6)	97.9 (93.4–102.7)	99.4 (94.8–104.2)	100.5 (95.9–105.4)

	50–69			
Year	Reference	Scenario 1	Scenario 2	Scenario 3
2017	680.3 (665.1–695.8)	682.4 (667.2–697.9)	682.4 (667.2–697.9)	682.4 (667.2–697.9)
2018	671.5 (627.9–718.1)	669.5 (626.1–716.0)	673.3 (629.6–720.0)	674.6 (630.8–721.4)
2019	667.9 (596.2–748.3)	662.0 (590.9–741.7)	669.5 (597.5–750.0)	672.1 (599.9–753.0)

2020	660.0 (558.1–780.5)	650.2 (549.8–769.0)	661.2 (559.1–782.0)	665.1 (562.4–786.6)
2021	653.9 (516.6–827.6)	640.4 (505.9–810.5)	654.8 (517.4–828.8)	660.0 (521.4–835.4)
2022	652.6 (481.4–884.6)	635.3 (468.7–861.2)	653.3 (481.9–885.5)	659.7 (486.7–894.3)
2023	647.9 (445.7–941.8)	626.9 (431.3–911.4)	648.3 (445.9–942.4)	656.0 (451.2–953.6)
2024	644.0 (409.7–1012.2)	622.6 (396.1–978.6)	644.1 (409.8–1012.4)	653.0 (415.5–1026.4)
2025	644.9 (379.4–1096.1)	622.9 (366.5–1058.7)	644.7 (379.3–1095.8)	655.0 (385.3–1113.2)
2026	643.0 (350.1–1180.9)	620.5 (337.8–1139.6)	642.6 (349.9–1180.2)	654.1 (356.1–1201.2)
2027	641.1 (321.4–1278.8)	618.1 (309.9–1233.0)	640.5 (321.1–1277.5)	653.2 (327.5–1302.9)
2028	644.0 (297.4–1394.7)	620.3 (286.4–1343.4)	643.0 (296.9–1392.7)	657.1 (303.4–1423.1)
2029	644.7 (274.7–1513.0)	620.4 (264.4–1456.1)	643.5 (274.2–1510.2)	658.9 (280.7–1546.3)
2030	644.8 (252.8–1644.9)	620.0 (243.1–1581.5)	643.4 (252.2–1641.1)	660.0 (258.8–1683.6)
2031	649.5 (234.4–1799.7)	623.9 (225.2–1728.7)	647.7 (233.8–1794.8)	665.8 (240.3–1845.0)
2032	652.6 (217.4–1959.1)	626.3 (208.6–1880.2)	650.6 (216.7–1953.1)	670.1 (223.2–2011.6)
2033	654.7 (201.0–2132.3)	627.7 (192.7–2044.5)	652.4 (200.3–2124.8)	673.2 (206.7–2192.8)
2034	661.0 (187.2–2334.0)	633.2 (179.3–2235.8)	658.4 (186.5–2324.8)	680.8 (192.8–2403.9)
2035	666.4 (174.6–2543.1)	637.8 (167.1–2433.8)	663.5 (173.9–2532.0)	687.5 (180.2–2623.3)
2036	670.4 (162.5–2765.5)	641.0 (155.4–2644.2)	667.2 (161.7–2752.3)	692.6 (167.9–2857.2)
2037	678.3 (152.3–3022.0)	648.0 (145.4–2886.9)	674.8 (151.5–3006.4)	701.9 (157.5–3127.1)
2038	685.9 (143.0–3289.3)	654.6 (136.5–3139.3)	682.1 (142.2–3270.9)	710.9 (148.2–3409.0)

2039	691.7 (134.1–3568.6)	659.6 (127.8–3402.7)	687.6 (133.3–3547.2)	718.0 (139.2–3704.1)
2040	701.2 (126.5–3887.2)	668.0 (120.5–3703.1)	696.7 (125.7–3862.3)	729.0 (131.5–4041.1)

	≥70			
Year	Reference	Scenario 1	Scenario 2	Scenario 3
2017	1944.8 (1900.3–1990.3)	1945.7 (1901.2–1991.2)	1945.7 (1901.2–1991.2)	1945.7 (1901.2–1991.2)
2018	1956.8 (1891.5–2024.3)	1953.6 (1888.5–2021.0)	1956.9 (1891.7–2024.5)	1958.1 (1892.8–2025.7)
2019	1979.5 (1912.5–2048.9)	1972.3 (1905.5–2041.4)	1979.0 (1912.0–2048.3)	1981.3 (1914.3–2050.7)
2020	2008.2 (1939.9–2078.9)	1996.7 (1928.8–2067.0)	2006.9 (1938.6–2077.6)	2010.5 (1942.1–2081.3)
2021	2025.6 (1956.3–2097.4)	2009.9 (1941.1–2081.1)	2023.5 (1954.3–2095.2)	2028.4 (1959.0–2100.2)
2022	2030.3 (1952.9–2110.7)	2010.4 (1933.7–2090.0)	2027.5 (1950.2–2107.8)	2033.5 (1956.0–2114.1)
2023	2022.6 (1940.2–2108.5)	1998.7 (1917.2–2083.6)	2019.1 (1936.8–2104.8)	2026.3 (1943.8–2112.4)
2024	1996.0 (1913.0–2082.6)	1971.0 (1889.1–2056.6)	1991.7 (1908.9–2078.1)	2000.1 (1916.9–2086.8)
2025	1953.4 (1869.4–2041.3)	1927.8 (1844.8–2014.5)	1948.5 (1864.7–2036.2)	1957.9 (1873.6–2045.9)
2026	1908.0 (1823.8–1996.1)	1881.8 (1798.7–1968.7)	1902.6 (1818.6–1990.4)	1912.8 (1828.4–2001.1)
2027	1863.5 (1781.4–1949.3)	1836.7 (1755.8–1921.3)	1857.4 (1775.6–1943.0)	1868.6 (1786.3–1954.7)
2028	1825.5 (1744.8–1910.0)	1798.1 (1718.6–1881.3)	1819.0 (1738.5–1903.1)	1831.0 (1750.0–1915.7)
2029	1800.7 (1719.2–1886.0)	1772.5 (1692.3–1856.5)	1793.5 (1712.4–1878.5)	1806.4 (1724.7–1892.0)

2030	1791.7 (1706.7–1881.0)	1762.6 (1679.0–1850.4)	1784.0 (1699.3–1872.8)	1797.9 (1712.6–1887.4)
2031	1793.1 (1704.0–1886.8)	1762.8 (1675.3–1854.9)	1784.6 (1696.0–1877.9)	1799.6 (1710.3–1893.7)
2032	1804.2 (1714.2–1898.9)	1772.6 (1684.2–1865.6)	1795.0 (1705.5–1889.3)	1811.2 (1720.8–1906.3)
2033	1819.3 (1728.4–1914.9)	1786.3 (1697.0–1880.2)	1809.4 (1719.0–1904.5)	1826.7 (1735.5–1922.8)
2034	1828.5 (1736.7–1925.2)	1794.2 (1704.1–1889.1)	1817.9 (1726.6–1914.0)	1836.5 (1744.2–1933.6)
2035	1828.2 (1735.3–1926.1)	1792.8 (1701.6–1888.7)	1816.9 (1724.6–1914.2)	1836.6 (1743.2–1934.9)
2036	1817.3 (1722.4–1917.3)	1780.9 (1688.0–1878.9)	1805.4 (1711.2–1904.8)	1826.0 (1730.7–1926.5)
2037	1792.2 (1697.3–1892.4)	1755.2 (1662.3–1853.3)	1779.8 (1685.6–1879.3)	1801.2 (1705.9–1901.9)
2038	1756.8 (1661.9–1857.1)	1719.4 (1626.5–1817.6)	1744.0 (1649.8–1843.5)	1766.0 (1670.6–1866.8)
2039	1718.4 (1624.7–1817.5)	1680.8 (1589.1–1777.7)	1705.3 (1612.3–1803.6)	1727.8 (1633.6–1827.5)
2040	1681.1 (1589.8–1777.6)	1643.2 (1554.0–1737.6)	1667.6 (1577.1–1763.4)	1690.7 (1598.9–1787.8)

	Women (2–B)			
	20–49			
Year	Reference	Scenario 1	Scenario 2	Scenario 3
2017	110.1 (108.1–112.1)	110.4 (108.5–112.5)	110.4 (108.5–112.5)	110.4 (108.5–112.5)
2018	108.8 (105.6–112.0)	109.1 (105.9–112.4)	109.2 (106.0–112.5)	109.2 (106.1–112.5)
2019	107.9 (103.9–112.0)	108.3 (104.3–112.4)	108.5 (104.5–112.6)	108.5 (104.5–112.7)

2020	106.9 (101.9–112.3)	107.4 (102.3–112.8)	107.7 (102.6–113.0)	107.8 (102.6–113.1)
2021	105.7 (99.9–111.8)	106.2 (100.4–112.3)	106.5 (100.7–112.7)	106.6 (100.8–112.8)
2022	104.9 (98.8–111.3)	105.5 (99.4–111.9)	105.9 (99.8–112.3)	106.0 (99.9–112.5)
2023	104.2 (97.9–110.9)	104.8 (98.5–111.6)	105.3 (98.9–112.1)	105.5 (99.1–112.3)
2024	103.4 (97.0–110.2)	104.0 (97.6–110.8)	104.6 (98.2–111.5)	104.8 (98.4–111.7)
2025	103.0 (96.6–109.8)	103.5 (97.1–110.3)	104.3 (97.9–111.2)	104.6 (98.1–111.4)
2026	102.6 (96.3–109.4)	103.1 (96.7–109.9)	104.1 (97.7–111.0)	104.4 (97.9–111.2)
2027	102.2 (95.9–108.9)	102.6 (96.2–109.4)	103.8 (97.3–110.6)	104.1 (97.6–110.9)
2028	101.9 (95.6–108.7)	102.3 (95.9–109.1)	103.6 (97.2–110.5)	104.0 (97.5–110.8)
2029	101.7 (95.3–108.5)	102.0 (95.6–108.8)	103.5 (97.1–110.4)	103.9 (97.4–110.8)
2030	101.3 (95.0–108.1)	101.6 (95.2–108.4)	103.3 (96.8–110.2)	103.6 (97.2–110.6)
2031	101.0 (94.7–107.8)	101.2 (94.9–108.0)	103.1 (96.6–110.0)	103.5 (97.0–110.5)
2032	100.7 (94.4–107.5)	100.9 (94.5–107.7)	102.9 (96.4–109.9)	103.4 (96.8–110.3)
2033	100.3 (94.0–107.1)	100.4 (94.1–107.2)	102.6 (96.2–109.5)	103.1 (96.6–110.0)
2034	99.9 (93.6–106.7)	100.0 (93.7–106.7)	102.4 (95.9–109.3)	102.8 (96.3–109.8)
2035	99.5 (93.2–106.2)	99.5 (93.3–106.2)	102.1 (95.6–109.0)	102.6 (96.1–109.5)
2036	99.1 (92.8–105.7)	99.0 (92.8–105.7)	101.7 (95.3–108.6)	102.3 (95.8–109.1)
2037	98.6 (92.4–105.3)	98.5 (92.3–105.2)	101.4 (95.0–108.2)	102.0 (95.5–108.8)
2038	98.2 (92.0–104.8)	98.1 (91.9–104.7)	101.1 (94.7–107.9)	101.7 (95.2–108.5)

2039	97.7 (91.5–104.3)	97.5 (91.4–104.1)	100.7 (94.4–107.5)	101.3 (94.9–108.2)
2040	97.3 (91.1–103.9)	97.1 (90.9–103.6)	100.4 (94.1–107.2)	101.0 (94.6–107.9)

	50–69			
Year	Reference	Scenario 1	Scenario 2	Scenario 3
2017	410.8 (405.4–416.3)	412.2 (406.7–417.7)	412.2 (406.7–417.7)	412.2 (406.7–417.7)
2018	403.7 (397.3–410.3)	403.9 (397.5–410.5)	405.3 (398.8–411.9)	405.7 (399.2–412.3)
2019	400.8 (394.3–407.3)	399.8 (393.4–406.4)	402.5 (396.0–409.1)	403.4 (396.9–410.0)
2020	401.3 (393.6–409.1)	399.2 (391.5–407.0)	403.2 (395.4–411.0)	404.6 (396.8–412.5)
2021	396.1 (388.5–403.9)	393.0 (385.5–400.7)	398.2 (390.6–406.0)	400.1 (392.4–407.9)
2022	394.1 (386.5–401.9)	389.9 (382.3–397.6)	396.4 (388.7–404.2)	398.7 (391.0–406.6)
2023	394.0 (386.4–401.8)	388.7 (381.2–396.3)	396.4 (388.8–404.3)	399.2 (391.5–407.1)
2024	389.8 (382.1–397.7)	384.0 (376.4–391.8)	392.4 (384.7–400.3)	395.6 (387.8–403.6)
2025	386.6 (378.1–395.3)	380.3 (372.0–388.9)	389.4 (380.9–398.1)	393.0 (384.4–401.9)
2026	385.5 (377.1–394.2)	378.7 (370.4–387.2)	388.5 (380.0–397.2)	392.6 (384.0–401.4)
2027	380.9 (372.4–389.5)	373.6 (365.3–382.1)	384.0 (375.5–392.7)	388.5 (379.9–397.3)
2028	377.0 (368.4–385.7)	369.3 (360.9–377.9)	380.3 (371.6–389.1)	385.2 (376.4–394.1)
2029	375.1 (366.6–383.7)	366.9 (358.6–375.4)	378.5 (370.0–387.2)	383.8 (375.2–392.7)

2030	370.7 (362.2–379.3)	362.1 (353.8–370.5)	374.2 (365.7–383.0)	379.9 (371.3–388.8)
2031	367.2 (358.8–375.8)	358.2 (350.0–366.5)	370.9 (362.5–379.6)	377.0 (368.4–385.8)
2032	365.2 (356.7–373.9)	355.7 (347.5–364.2)	369.1 (360.5–377.9)	375.6 (366.9–384.6)
2033	361.9 (353.3–370.8)	352.0 (343.7–360.6)	366.0 (357.3–374.9)	372.8 (364.0–381.9)
2034	358.9 (350.4–367.6)	348.6 (340.3–357.1)	363.1 (354.5–371.9)	370.3 (361.6–379.3)
2035	357.4 (348.9–366.1)	346.7 (338.4–355.1)	361.7 (353.1–370.6)	369.4 (360.6–378.4)
2036	354.5 (346.1–363.1)	343.3 (335.2–351.7)	359.0 (350.4–367.7)	367.0 (358.3–375.9)
2037	351.8 (343.3–360.4)	340.2 (332.1–348.6)	356.4 (347.8–365.1)	364.8 (356.0–373.7)
2038	350.0 (341.6–358.6)	338.0 (329.9–346.3)	354.7 (346.2–363.4)	363.5 (354.8–372.4)
2039	346.9 (338.6–355.5)	334.6 (326.6–342.8)	351.8 (343.4–360.5)	360.9 (352.3–369.8)
2040	344.0 (335.7–352.6)	331.3 (323.3–339.6)	349.0 (340.5–357.7)	358.5 (349.8–367.4)

	≥70			
Year	Reference	Scenario 1	Scenario 2	Scenario 3
2017	1464.8 (1434.8–1495.4)	1465.9 (1435.9–1496.5)	1465.9 (1435.9–1496.5)	1465.9 (1435.9–1496.5)
2018	1434.3 (1393.4–1476.4)	1432.4 (1391.5–1474.4)	1435.1 (1394.2–1477.2)	1436.0 (1395.1–1478.1)
2019	1427.0 (1380.5–1475.0)	1422.0 (1375.7–1469.8)	1427.3 (1380.9–1475.3)	1429.2 (1382.7–1477.3)
2020	1404.9 (1353.1–1458.8)	1397.0 (1345.5–1450.6)	1404.9 (1353.1–1458.7)	1407.7 (1355.8–1461.6)

2021	1392.4 (1341.4–1445.3)	1381.6 (1331.0–1434.1)	1392.0 (1341.0–1444.9)	1395.7 (1344.6–1448.7)
2022	1394.8 (1343.6–1448.0)	1381.0 (1330.3–1433.7)	1394.0 (1342.8–1447.2)	1398.7 (1347.3–1452.0)
2023	1377.2 (1326.6–1429.6)	1360.6 (1310.7–1412.5)	1376.0 (1325.5–1428.4)	1381.5 (1330.8–1434.1)
2024	1373.1 (1320.8–1427.3)	1354.6 (1303.1–1408.2)	1371.6 (1319.4–1425.8)	1377.9 (1325.5–1432.4)
2025	1371.5 (1317.6–1427.5)	1351.0 (1298.0–1406.2)	1369.6 (1315.9–1425.6)	1376.9 (1322.8–1433.1)
2026	1355.6 (1299.9–1413.6)	1333.4 (1278.6–1390.5)	1353.4 (1297.8–1411.3)	1361.4 (1305.5–1419.8)
2027	1354.1 (1294.1–1416.8)	1330.0 (1271.1–1391.7)	1351.5 (1291.7–1414.2)	1360.5 (1300.3–1423.6)
2028	1343.6 (1283.2–1406.9)	1317.8 (1258.6–1379.8)	1340.7 (1280.5–1403.8)	1350.5 (1289.8–1414.1)
2029	1324.7 (1264.3–1388.0)	1297.3 (1238.1–1359.3)	1321.5 (1261.2–1384.6)	1332.0 (1271.3–1395.6)
2030	1317.2 (1256.9–1380.4)	1288.1 (1229.1–1349.9)	1313.6 (1253.5–1376.7)	1325.0 (1264.3–1388.6)
2031	1297.7 (1238.1–1360.0)	1267.1 (1209.0–1328.0)	1293.8 (1234.5–1356.0)	1305.8 (1245.9–1368.6)
2032	1279.4 (1220.5–1341.2)	1247.4 (1190.0–1307.7)	1275.3 (1216.5–1336.8)	1288.0 (1228.7–1350.2)
2033	1268.8 (1209.5–1330.9)	1235.3 (1177.6–1295.8)	1264.4 (1205.3–1326.3)	1277.8 (1218.1–1340.4)
2034	1248.0 (1187.7–1311.3)	1213.2 (1154.7–1274.8)	1243.3 (1183.3–1306.3)	1257.3 (1196.6–1321.1)
2035	1234.9 (1174.5–1298.5)	1198.8 (1140.1–1260.5)	1229.9 (1169.7–1293.2)	1244.7 (1183.7–1308.7)
2036	1225.0 (1163.9–1289.3)	1187.4 (1128.1–1249.7)	1219.7 (1158.9–1283.8)	1235.1 (1173.5–1300.0)
2037	1209.0 (1148.1–1273.1)	1170.1 (1111.2–1232.2)	1203.5 (1142.8–1267.3)	1219.5 (1158.1–1284.2)
2038	1202.5 (1142.0–1266.3)	1162.2 (1103.7–1223.8)	1196.7 (1136.5–1260.2)	1213.5 (1152.4–1277.8)
2039	1194.2 (1134.0–1257.5)	1152.4 (1094.4–1213.6)	1188.1 (1128.2–1251.1)	1205.5 (1144.8–1269.5)

2040	1183.0 (1123.3–1245.9)	1140.0 (1082.5–1200.6)	1176.7 (1117.3–1239.2)	1194.7 (1134.4–1258.2)

	Both sexes combined (2–C)			
	20–49			
Year	Reference	Scenario 1	Scenario 2	Scenario 3
2017	113.2 (111.4–115.0)	113.4 (111.6–115.2)	113.4 (111.6–115.2)	113.4 (111.6–115.2)
2018	110.8 (108.5–113.3)	110.9 (108.6–113.4)	111.0 (108.7–113.5)	111.1 (108.7–113.5)
2019	109.4 (106.6–112.3)	109.5 (106.7–112.3)	109.6 (106.8–112.5)	109.7 (106.9–112.6)
2020	107.6 (104.5–110.9)	107.6 (104.5–110.9)	107.9 (104.8–111.1)	108.0 (104.9–111.2)
2021	106.3 (103.1–109.5)	106.2 (103.1–109.4)	106.6 (103.4–109.8)	106.7 (103.5–109.9)
2022	106.3 (103.2–109.5)	106.2 (103.0–109.4)	106.6 (103.5–109.9)	106.8 (103.6–110.0)
2023	105.5 (102.4–108.7)	105.3 (102.2–108.5)	105.9 (102.7–109.1)	106.0 (102.9–109.3)
2024	105.4 (102.2–108.6)	105.2 (102.0–108.5)	105.8 (102.6–109.1)	106.0 (102.8–109.3)
2025	106.1 (102.8–109.5)	105.9 (102.6–109.3)	106.5 (103.2–109.9)	106.8 (103.4–110.2)
2026	106.2 (102.8–109.7)	105.9 (102.6–109.4)	106.7 (103.3–110.2)	106.9 (103.5–110.5)
2027	106.6 (103.0–110.3)	106.3 (102.7–110.1)	107.1 (103.5–110.9)	107.4 (103.8–111.2)
2028	107.5 (103.6–111.6)	107.2 (103.3–111.3)	108.1 (104.2–112.2)	108.4 (104.5–112.5)
2029	107.2 (103.3–111.3)	107.0 (103.0–111.1)	107.9 (103.9–112.0)	108.3 (104.3–112.4)

2030	107.1 (103.1–111.3)	106.8 (102.8–111.0)	107.7 (103.7–112.0)	108.2 (104.1–112.4)
2031	106.9 (102.8–111.1)	106.5 (102.5–110.7)	107.6 (103.5–111.8)	108.0 (103.9–112.2)
2032	105.6 (101.6–109.7)	105.2 (101.2–109.4)	106.3 (102.3–110.5)	106.8 (102.7–111.0)
2033	104.3 (100.4–108.5)	104.0 (100.0–108.1)	105.1 (101.1–109.3)	105.6 (101.6–109.8)
2034	103.2 (99.2–107.4)	102.9 (98.9–107.0)	104.0 (100.0–108.2)	104.6 (100.5–108.8)
2035	101.4 (97.3–105.6)	101.0 (96.9–105.2)	102.2 (98.1–106.5)	102.7 (98.6–107.0)
2036	99.9 (95.8–104.2)	99.5 (95.5–103.8)	100.8 (96.6–105.1)	101.3 (97.2–105.7)
2037	98.8 (94.6–103.1)	98.4 (94.2–102.7)	99.6 (95.5–104.0)	100.2 (96.0–104.6)
2038	97.3 (93.1–101.6)	96.9 (92.7–101.2)	98.2 (93.9–102.6)	98.8 (94.5–103.2)
2039	96.5 (92.3–100.8)	96.0 (91.9–100.4)	97.4 (93.2–101.8)	98.0 (93.8–102.4)
2040	96.1 (91.9–100.4)	95.6 (91.5–99.9)	97.0 (92.9–101.4)	97.7 (93.5–102.1)

	50–69			
Year	Reference	Scenario 1	Scenario 2	Scenario 3
2017	552.9 (545.7–560.2)	554.7 (547.5–561.9)	554.7 (547.5–561.9)	554.7 (547.5–561.9)
2018	551.2 (534.2–568.7)	550.5 (533.5–568.1)	552.9 (535.9–570.6)	553.8 (536.7–571.5)
2019	557.5 (534.8–581.2)	554.4 (531.9–578.0)	559.3 (536.6–583.1)	561.1 (538.2–584.9)
2020	566.1 (539.0–594.6)	560.5 (533.7–588.7)	568.0 (540.8–596.6)	570.6 (543.3–599.4)

2021	567.9 (537.2–600.3)	559.8 (529.6–591.8)	569.8 (539.0–602.3)	573.3 (542.4–606.0)
2022	572.7 (541.1–606.2)	562.1 (531.1–594.9)	574.6 (542.9–608.2)	579.1 (547.2–612.9)
2023	576.9 (545.0–610.6)	563.7 (532.6–596.6)	578.8 (546.9–612.6)	584.2 (552.0–618.4)
2024	571.9 (540.3–605.3)	558.2 (527.3–590.8)	573.8 (542.1–607.3)	580.1 (548.0–614.0)
2025	568.7 (536.4–603.0)	554.5 (522.9–587.9)	570.7 (538.2–605.1)	577.8 (544.9–612.6)
2026	565.4 (531.7–601.3)	550.6 (517.8–585.5)	567.4 (533.5–603.3)	575.4 (541.1–611.8)
2027	555.2 (521.2–591.5)	540.1 (507.0–575.3)	557.1 (523.0–593.5)	565.9 (531.2–602.8)
2028	548.9 (514.1–586.2)	533.4 (499.5–569.5)	550.9 (515.9–588.2)	560.4 (524.8–598.3)
2029	545.1 (509.9–582.8)	529.1 (494.9–565.6)	547.0 (511.7–584.9)	557.3 (521.3–595.9)
2030	537.2 (502.5–574.4)	520.8 (487.1–556.8)	539.1 (504.3–576.4)	550.1 (514.6–588.2)
2031	534.5 (500.0–571.5)	517.6 (484.1–553.4)	536.5 (501.8–573.6)	548.3 (512.8–586.2)
2032	535.1 (500.4–572.2)	517.6 (484.0–553.4)	537.0 (502.2–574.3)	549.7 (514.1–587.8)
2033	532.0 (497.0–569.5)	514.0 (480.1–550.2)	533.9 (498.8–571.6)	547.4 (511.3–586.0)
2034	533.1 (497.6–571.1)	514.4 (480.2–551.1)	535.0 (499.5–573.1)	549.4 (512.8–588.5)
2035	536.2 (500.2–574.7)	516.8 (482.2–554.0)	538.1 (502.0–576.8)	553.4 (516.3–593.2)
2036	534.3 (498.0–573.2)	514.4 (479.5–551.9)	536.3 (499.9–575.4)	552.4 (514.9–592.6)
2037	534.9 (498.5–573.9)	514.4 (479.5–552.0)	536.9 (500.4–576.1)	553.9 (516.2–594.3)
2038	536.2 (499.8–575.4)	515.1 (480.1–552.7)	538.3 (501.7–577.5)	556.1 (518.3–596.7)
2039	532.1 (495.9–570.9)	510.5 (475.8–547.8)	534.1 (497.8–573.1)	552.7 (515.1–593.0)

2040	529.8 (493.7–568.6)	507.8 (473.2–545.0)	531.8 (495.6–570.8)	551.2 (513.6–591.6)

	≥70			
Year	Reference	Scenario 1	Scenario 2	Scenario 3
2017	1679.7 (1647.8–1712.3)	1680.8 (1648.9–1713.4)	1680.8 (1648.9–1713.4)	1680.8 (1648.9–1713.4)
2018	1665.4 (1621.0–1710.9)	1662.4 (1618.1–1707.9)	1665.8 (1621.4–1711.4)	1667.0 (1622.6–1712.6)
2019	1675.6 (1629.6–1723.0)	1668.6 (1622.8–1715.7)	1675.4 (1629.4–1722.7)	1677.8 (1631.7–1725.2)
2020	1687.8 (1636.9–1740.3)	1676.6 (1626.1–1728.7)	1686.9 (1636.0–1739.3)	1690.5 (1639.6–1743.1)
2021	1683.1 (1631.1–1736.9)	1668.0 (1616.3–1721.2)	1681.6 (1629.6–1735.3)	1686.4 (1634.2–1740.3)
2022	1684.3 (1631.6–1738.6)	1665.0 (1613.0–1718.7)	1682.0 (1629.5–1736.3)	1688.1 (1635.3–1742.6)
2023	1683.0 (1628.3–1739.5)	1659.7 (1605.8–1715.4)	1680.1 (1625.5–1736.5)	1687.3 (1632.6–1744.0)
2024	1662.8 (1606.7–1720.7)	1637.9 (1582.8–1695.0)	1659.2 (1603.3–1717.1)	1667.6 (1611.4–1725.7)
2025	1640.4 (1579.9–1703.2)	1614.1 (1554.6–1675.9)	1636.3 (1576.0–1698.9)	1645.7 (1585.0–1708.7)
2026	1620.8 (1558.1–1686.1)	1593.1 (1531.4–1657.2)	1616.1 (1553.6–1681.2)	1626.6 (1563.7–1692.1)
2027	1593.9 (1532.1–1658.1)	1564.8 (1504.2–1627.9)	1588.6 (1527.0–1652.6)	1600.0 (1538.1–1664.5)
2028	1569.4 (1508.6–1632.7)	1539.1 (1479.5–1601.2)	1563.6 (1503.0–1626.7)	1576.0 (1515.0–1639.6)
2029	1555.2 (1494.8–1618.0)	1523.5 (1464.4–1585.0)	1548.9 (1488.7–1611.4)	1562.3 (1501.6–1625.4)
2030	1539.5 (1477.9–1603.7)	1506.5 (1446.2–1569.2)	1532.6 (1471.3–1596.5)	1547.0 (1485.1–1611.4)

2031	1527.4 (1464.2–1593.3)	1493.0 (1431.2–1557.4)	1520.0 (1457.1–1585.6)	1535.3 (1471.8–1601.6)
2032	1523.5 (1459.0–1590.7)	1487.4 (1424.5–1553.1)	1515.4 (1451.4–1582.3)	1531.8 (1467.1–1599.5)
2033	1520.4 (1454.9–1588.9)	1482.8 (1418.9–1549.6)	1511.8 (1446.7–1579.9)	1529.3 (1463.4–1598.2)
2034	1514.6 (1449.1–1583.1)	1475.5 (1411.7–1542.2)	1505.5 (1440.4–1573.5)	1524.0 (1458.1–1592.9)
2035	1508.8 (1443.6–1576.9)	1468.2 (1404.8–1534.5)	1499.1 (1434.3–1566.8)	1518.6 (1452.9–1587.2)
2036	1499.2 (1434.1–1567.3)	1457.3 (1393.9–1523.5)	1489.0 (1424.3–1556.6)	1509.4 (1443.8–1578.0)
2037	1483.2 (1417.9–1551.5)	1440.1 (1376.7–1506.4)	1472.5 (1407.7–1540.3)	1493.8 (1428.0–1562.6)
2038	1464.5 (1398.7–1533.5)	1420.4 (1356.5–1487.3)	1453.4 (1388.0–1521.8)	1475.5 (1409.1–1544.9)
2039	1444.3 (1378.4–1513.3)	1399.2 (1335.4–1466.1)	1432.7 (1367.4–1501.2)	1455.5 (1389.1–1525.1)
2040	1422.1 (1356.9–1490.3)	1376.1 (1313.1–1442.2)	1410.1 (1345.5–1477.8)	1433.6 (1367.9–1502.4)

	All age (2–D)			
	Men			
Year	Reference	Scenario 1	Scenario 2	Scenario 3
2017	555.5 (542.3–569.1)	558.7 (545.5–572.4)	558.7 (545.5–572.4)	558.7 (545.5–572.4)
2018	561.0 (541.5–581.3)	560.7 (541.1–581.0)	564.5 (544.8–584.9)	565.9 (546.1–586.3)
2019	570.3 (543.5–598.5)	566.1 (539.5–594.1)	573.9 (546.9–602.2)	576.6 (549.5–605.1)
2020	581.7 (549.8–615.4)	573.5 (542.1–606.8)	585.4 (553.3–619.3)	589.6 (557.3–623.7)

2021	594.3 (558.7–632.3)	582.1 (547.2–619.2)	598.1 (562.2–636.3)	603.9 (567.6–642.4)
2022	607.3 (569.3–647.8)	590.7 (553.8–630.1)	611.2 (573.0–651.9)	618.5 (579.9–659.8)
2023	619.7 (580.4–661.7)	598.8 (560.8–639.3)	623.7 (584.1–665.9)	632.7 (592.6–675.6)
2024	631.1 (591.0–673.9)	609.1 (570.4–650.5)	635.1 (594.8–678.2)	645.9 (604.9–689.7)
2025	641.0 (600.2–684.5)	618.1 (578.8–660.0)	645.2 (604.2–688.9)	657.7 (615.9–702.3)
2026	649.4 (607.9–693.7)	625.6 (585.6–668.2)	653.7 (611.9–698.2)	667.9 (625.3–713.5)
2027	656.4 (614.1–701.6)	631.7 (591.0–675.2)	660.7 (618.2–706.2)	676.8 (633.2–723.4)
2028	662.3 (619.2–708.5)	636.7 (595.3–681.1)	666.7 (623.3–713.2)	684.6 (640.0–732.2)
2029	667.5 (623.6–714.5)	641.1 (598.9–686.2)	672.0 (627.8–719.3)	691.6 (646.1–740.3)
2030	672.3 (627.8–720.0)	645.0 (602.3–690.8)	676.9 (632.0–724.9)	698.3 (652.1–747.8)
2031	677.2 (632.2–725.4)	649.0 (605.9–695.2)	681.8 (636.5–730.3)	705.0 (658.2–755.2)
2032	682.3 (636.9–730.9)	653.3 (609.8–699.9)	687.0 (641.2–735.9)	712.1 (664.7–762.9)
2033	687.9 (642.1–737.0)	658.0 (614.2–705.0)	692.7 (646.6–742.1)	719.8 (671.8–771.1)
2034	694.2 (648.0–743.7)	663.3 (619.2–710.6)	699.0 (652.5–748.8)	728.1 (679.6–780.0)
2035	701.1 (654.4–751.1)	669.2 (624.7–717.0)	705.9 (658.9–756.3)	737.1 (688.0–789.7)
2036	708.5 (661.3–759.1)	675.7 (630.6–723.9)	713.5 (665.9–764.5)	746.8 (697.0–800.1)
2037	716.5 (668.7–767.7)	682.6 (637.0–731.4)	721.6 (673.4–773.1)	757.0 (706.5–811.1)
2038	724.8 (676.4–776.7)	689.8 (643.8–739.2)	730.0 (681.2–782.2)	767.7 (716.4–822.6)
2039	733.4 (684.4–785.9)	697.3 (650.7–747.2)	738.7 (689.3–791.6)	778.7 (726.6–834.5)

2040	742.1 (692.5–795.3)	704.9 (657.7–755.4)	747.5 (697.5–801.0)	789.8 (737.0–846.4)

	Women			
Year	Reference	Scenario 1	Scenario 2	Scenario 3
2017	483.7 (474.4–493.1)	484.0 (474.7–493.5)	484.0 (474.7–493.5)	484.0 (474.7–493.5)
2018	486.7 (475.4–498.2)	486.8 (475.6–498.4)	487.1 (475.8–498.6)	487.2 (475.9–498.7)
2019	490.0 (477.9–502.5)	490.0 (477.9–502.5)	490.6 (478.4–503.0)	490.7 (478.6–503.2)
2020	493.3 (480.8–506.2)	493.2 (480.6–506.1)	493.9 (481.4–506.8)	494.2 (481.6–507.1)
2021	496.7 (483.9–509.9)	496.4 (483.6–509.5)	497.4 (484.6–510.6)	497.8 (485.0–511.0)
2022	500.1 (487.2–513.4)	499.7 (486.7–512.9)	500.9 (488.0–514.3)	501.4 (488.4–514.7)
2023	503.6 (490.5–517.0)	502.9 (489.9–516.3)	504.5 (491.4–517.9)	505.0 (491.9–518.5)
2024	507.1 (493.9–520.6)	506.3 (493.1–519.8)	508.1 (494.8–521.6)	508.7 (495.5–522.3)
2025	510.6 (497.3–524.2)	509.7 (496.4–523.3)	511.7 (498.4–525.3)	512.4 (499.1–526.1)
2026	514.1 (500.7–527.9)	513.1 (499.7–526.8)	515.3 (501.9–529.1)	516.2 (502.7–529.9)
2027	517.7 (504.2–531.5)	516.5 (503.0–530.3)	519.0 (505.5–532.8)	519.9 (506.4–533.8)
2028	521.3 (507.7–535.2)	519.9 (506.4–533.8)	522.7 (509.1–536.6)	523.7 (510.1–537.7)
2029	524.9 (511.2–538.9)	523.4 (509.8–537.4)	526.4 (512.7–540.5)	527.5 (513.8–541.6)
2030	528.5 (514.8–542.7)	526.9 (513.2–541.0)	530.1 (516.3–544.3)	531.4 (517.5–545.6)

2031	532.2 (518.4–546.4)	530.4 (516.6–544.6)	533.9 (520.0–548.2)	535.3 (521.3–549.6)
2032	535.9 (522.0–550.2)	533.9 (520.0–548.2)	537.7 (523.7–552.1)	539.2 (525.1–553.6)
2033	539.6 (525.6–554.1)	537.5 (523.5–551.9)	541.5 (527.4–556.0)	543.1 (529.0–557.6)
2034	543.4 (529.2–557.9)	541.1 (527.0–555.6)	545.4 (531.2–560.0)	547.1 (532.8–561.7)
2035	547.2 (532.9–561.8)	544.7 (530.5–559.3)	549.3 (535.0–564.0)	551.1 (536.7–565.8)
2036	550.9 (536.6–565.7)	548.4 (534.1–563.0)	553.2 (538.8–568.0)	555.1 (540.6–569.9)
2037	554.8 (540.3–569.6)	552.0 (537.6–566.8)	557.1 (542.6–572.0)	559.1 (544.6–574.1)
2038	558.6 (544.1–573.6)	555.7 (541.2–570.6)	561.1 (546.5–576.1)	563.2 (548.6–578.3)
2039	562.5 (547.9–577.5)	559.4 (544.9–574.4)	565.1 (550.4–580.2)	567.3 (552.6–582.5)
2040	566.4 (551.7–581.6)	563.2 (548.5–578.2)	569.1 (554.3–584.3)	571.5 (556.6–586.8)

	Both sexes combined			
Year	Reference	Scenario 1	Scenario 2	Scenario 3
2017	526.2 (516.8–535.9)	527.3 (517.8–537.0)	527.3 (517.8–537.0)	527.3 (517.8–537.0)
2018	533.3 (522.0–544.8)	533.4 (522.1–544.9)	534.5 (523.2–546.1)	534.9 (523.6–546.5)
2019	544.2 (533.6–555.0)	543.3 (532.7–554.1)	545.6 (535.0–556.4)	546.4 (535.7–557.2)
2020	557.3 (545.8–569.0)	555.4 (544.0–567.1)	558.9 (547.4–570.6)	560.1 (548.6–571.9)
2021	564.5 (553.2–576.1)	561.7 (550.4–573.2)	566.3 (555.0–577.9)	568.0 (556.6–579.6)

2022	564.8 (551.9–578.1)	561.0 (548.1–574.1)	566.8 (553.8–580.1)	568.9 (555.8–582.2)
2023	570.6 (557.2–584.2)	565.6 (552.4–579.2)	572.7 (559.3–586.4)	575.2 (561.8–589.0)
2024	567.0 (554.0–580.2)	561.8 (549.0–574.9)	569.2 (556.3–582.5)	572.2 (559.1–585.5)
2025	561.7 (547.8–575.9)	556.3 (542.5–570.4)	564.1 (550.2–578.4)	567.4 (553.4–581.8)
2026	562.0 (548.7–575.6)	556.3 (543.1–569.8)	564.6 (551.2–578.3)	568.3 (554.8–582.1)
2027	564.3 (549.7–579.3)	558.3 (543.8–573.1)	567.0 (552.4–582.1)	571.2 (556.4–586.4)
2028	566.1 (551.5–581.2)	559.8 (545.3–574.7)	569.1 (554.3–584.2)	573.6 (558.8–588.9)
2029	578.7 (563.2–594.6)	571.9 (556.6–587.7)	581.8 (566.3–597.8)	587.0 (571.2–603.1)
2030	589.5 (572.8–606.7)	582.3 (565.8–599.3)	592.8 (576.1–610.1)	598.5 (581.5–615.9)
2031	598.0 (581.8–614.8)	590.4 (574.4–606.9)	601.6 (585.3–618.4)	607.8 (591.3–624.8)
2032	608.7 (592.0–625.9)	600.7 (584.2–617.7)	612.6 (595.7–629.9)	619.3 (602.3–636.8)
2033	616.2 (599.2–633.6)	607.7 (590.9–624.9)	620.2 (603.1–637.8)	627.5 (610.2–645.3)
2034	614.3 (596.2–632.9)	605.5 (587.7–623.9)	618.5 (600.3–637.2)	626.2 (607.8–645.2)
2035	615.3 (596.7–634.4)	606.2 (587.9–625.0)	619.7 (601.0–638.9)	627.8 (608.9–647.4)
2036	613.5 (595.4–632.1)	604.1 (586.3–622.4)	618.0 (599.8–636.8)	626.7 (608.2–645.7)
2037	609.5 (591.4–628.2)	599.9 (582.1–618.3)	614.2 (596.0–633.0)	623.2 (604.7–642.3)
2038	611.4 (593.2–630.2)	601.5 (583.6–619.9)	616.3 (598.0–635.2)	625.8 (607.2–645.0)
2039	618.4 (599.0–638.3)	608.0 (589.0–627.6)	623.5 (604.0–643.6)	633.6 (613.8–654.0)

2040	623.7 (604.1–643.9)	612.9 (593.7–632.7)	629.0 (609.3–649.4)	639.7 (619.6–660.4)

	Stomach cancer (3)			
	Men (3–A)			
	20–49			
Year	Reference	Scenario 1	Scenario 2	Scenario 3
2017	107.6 (104.3–111.0)	109.2 (105.9–112.7)	109.2 (105.9–112.7)	109.2 (105.9–112.7)
2018	102.0 (97.0–107.2)	101.0 (96.0–106.2)	104.7 (99.6–110.1)	106.0 (100.8–111.5)
2019	96.6 (91.9–101.6)	92.7 (88.2–97.5)	99.7 (94.8–104.8)	102.3 (97.3–107.5)
2020	89.6 (84.9–94.6)	83.3 (79.0–88.0)	92.9 (88.0–98.1)	96.6 (91.5–101.9)
2021	89.5 (84.7–94.5)	80.7 (76.4–85.2)	93.3 (88.3–98.5)	98.2 (93.0–103.7)
2022	87.9 (82.7–93.4)	76.8 (72.3–81.6)	92.1 (86.7–97.8)	98.2 (92.4–104.3)
2023	83.8 (78.9–89.1)	71.0 (66.8–75.5)	88.3 (83.0–93.9)	95.3 (89.7–101.3)
2024	82.2 (77.1–87.7)	69.4 (65.0–74.0)	87.0 (81.6–92.8)	95.1 (89.2–101.5)
2025	79.1 (74.0–84.4)	66.5 (62.2–71.0)	84.1 (78.7–89.8)	93.1 (87.2–99.4)
2026	74.3 (69.5–79.4)	62.2 (58.2–66.5)	79.4 (74.3–84.9)	89.1 (83.4–95.2)
2027	71.6 (67.0–76.5)	59.7 (55.9–63.8)	76.9 (71.9–82.2)	87.4 (81.7–93.4)
2028	69.8 (65.2–74.7)	58.0 (54.1–62.1)	75.3 (70.3–80.7)	86.7 (81.0–92.9)

2029	69.3 (64.7–74.2)	57.4 (53.6–61.4)	75.2 (70.3–80.6)	87.7 (81.9–93.9)
2030	67.6 (63.2–72.4)	55.8 (52.1–59.7)	73.8 (68.9–79.0)	87.1 (81.3–93.3)
2031	63.4 (59.1–68.1)	52.1 (48.5–55.9)	69.5 (64.8–74.6)	83.1 (77.5–89.2)
2032	60.4 (56.3–64.8)	49.4 (46.0–53.0)	66.5 (62.0–71.4)	80.6 (75.1–86.5)
2033	58.7 (54.5–63.1)	47.8 (44.4–51.4)	64.9 (60.4–69.8)	79.7 (74.1–85.7)
2034	56.5 (52.5–60.8)	45.9 (42.7–49.3)	62.9 (58.5–67.6)	78.1 (72.7–84.0)
2035	54.8 (51.0–59.0)	44.3 (41.2–47.7)	61.3 (57.0–65.9)	77.2 (71.8–83.0)
2036	54.2 (50.4–58.3)	43.6 (40.6–46.9)	60.9 (56.6–65.5)	77.6 (72.2–83.5)
2037	52.4 (48.7–56.4)	42.1 (39.1–45.2)	59.2 (55.0–63.7)	76.5 (71.1–82.3)
2038	49.2 (45.7–52.9)	39.3 (36.5–42.3)	55.9 (51.9–60.1)	73.1 (67.9–78.6)
2039	46.7 (43.4–50.2)	37.1 (34.5–39.9)	53.2 (49.5–57.3)	70.5 (65.6–75.9)
2040	45.5 (42.3–49.0)	36.1 (33.5–38.9)	52.2 (48.5–56.2)	70.1 (65.1–75.5)

	50–69			
Year	Reference	Scenario 1	Scenario 2	Scenario 3
2017	1399.7 (1353.2–1447.8)	1418.9 (1371.7–1467.6)	1418.9 (1371.7–1467.6)	1418.9 (1371.7–1467.6)
2018	1369.9 (1324.4–1417.0)	1352.7 (1307.7–1399.2)	1386.2 (1340.1–1433.8)	1398.2 (1351.7–1446.3)
2019	1339.6 (1295.1–1385.6)	1288.4 (1245.6–1332.7)	1353.0 (1308.1–1399.5)	1376.6 (1330.9–1423.9)

2020	1310.7 (1267.1–1355.7)	1227.9 (1187.1–1270.1)	1321.4 (1277.5–1366.8)	1356.1 (1311.0–1402.7)
2021	1281.9 (1239.3–1326.0)	1169.8 (1130.9–1210.0)	1290.1 (1247.2–1334.5)	1335.5 (1291.1–1381.4)
2022	1254.0 (1212.3–1297.1)	1114.6 (1077.5–1152.9)	1259.7 (1217.8–1303.0)	1315.3 (1271.6–1360.5)
2023	1226.6 (1185.9–1268.8)	1062.0 (1026.7–1098.5)	1230.0 (1189.1–1272.3)	1295.4 (1252.4–1339.9)
2024	1199.9 (1160.0–1241.1)	1034.6 (1000.2–1070.2)	1200.9 (1161.0–1242.2)	1275.8 (1233.4–1319.7)
2025	1173.7 (1134.7–1214.0)	1008.0 (974.5–1042.6)	1172.6 (1133.6–1212.9)	1256.5 (1214.8–1299.7)
2026	1148.1 (1109.9–1187.6)	982.0 (949.4–1015.7)	1144.9 (1106.9–1184.3)	1237.5 (1196.4–1280.0)
2027	1123.0 (1085.7–1161.6)	956.7 (924.9–989.6)	1117.9 (1080.8–1156.4)	1218.8 (1178.3–1260.7)
2028	1098.5 (1062.0–1136.3)	932.0 (901.1–964.1)	1091.6 (1055.3–1129.1)	1200.4 (1160.5–1241.6)
2029	1074.6 (1038.9–1111.5)	908.0 (877.8–939.2)	1065.8 (1030.4–1102.4)	1182.2 (1142.9–1222.8)
2030	1051.1 (1016.2–1087.3)	884.6 (855.2–915.0)	1040.6 (1006.1–1076.4)	1164.3 (1125.6–1204.3)
2031	1028.2 (994.0–1063.5)	861.8 (833.2–891.4)	1016.1 (982.3–1051.0)	1146.7 (1108.6–1186.1)
2032	1005.8 (972.4–1040.3)	839.6 (811.7–868.5)	992.1 (959.2–1026.2)	1129.4 (1091.8–1168.2)
2033	983.8 (951.1–1017.6)	818.0 (790.8–846.1)	968.7 (936.5–1002.0)	1112.3 (1075.3–1150.5)
2034	962.4 (930.4–995.4)	796.9 (770.4–824.3)	945.9 (914.4–978.4)	1095.5 (1059.1–1133.1)
2035	941.4 (910.1–973.7)	776.4 (750.6–803.0)	923.5 (892.8–955.3)	1078.9 (1043.1–1116.0)
2036	920.8 (890.2–952.5)	756.4 (731.2–782.4)	901.7 (871.8–932.7)	1062.6 (1027.3–1099.1)
2037	900.7 (870.8–931.7)	736.9 (712.4–762.2)	880.5 (851.2–910.7)	1046.5 (1011.7–1082.5)
2038	881.1 (851.8–911.4)	717.9 (694.0–742.6)	859.7 (831.1–889.2)	1030.7 (996.4–1066.1)

2039	861.9 (833.2–891.5)	699.4 (676.1–723.4)	839.4 (811.5–868.3)	1015.1 (981.4–1050.0)
2040	843.1 (815.1–872.0)	681.4 (658.7–704.8)	819.6 (792.4–847.8)	999.8 (966.5–1034.1)

	≥70			
Year	Reference	Scenario 1	Scenario 2	Scenario 3
2017	3011.4 (2924.3–3101.0)	3015.6 (2928.5–3105.4)	3015.6 (2928.5–3105.4)	3015.6 (2928.5–3105.4)
2018	2967.1 (2867.5–3070.2)	2952.4 (2853.2–3054.9)	2967.9 (2868.3–3071.0)	2973.4 (2873.6–3076.7)
2019	2832.4 (2709.4–2961.1)	2800.4 (2678.8–2927.6)	2830.0 (2707.0–2958.5)	2840.5 (2717.1–2969.4)
2020	2832.5 (2708.8–2961.9)	2782.6 (2661.1–2909.7)	2826.8 (2703.4–2955.9)	2842.6 (2718.4–2972.3)
2021	2774.0 (2633.8–2921.7)	2707.8 (2570.9–2851.9)	2765.2 (2625.5–2912.4)	2785.8 (2645.0–2934.1)
2022	2772.9 (2625.7–2928.3)	2689.5 (2546.7–2840.2)	2761.0 (2614.4–2915.7)	2786.7 (2638.8–2942.8)
2023	2709.8 (2534.6–2897.1)	2611.5 (2442.6–2792.0)	2695.0 (2520.8–2881.3)	2725.2 (2549.0–2913.5)
2024	2675.5 (2487.9–2877.2)	2573.3 (2392.9–2767.4)	2657.9 (2471.5–2858.3)	2692.6 (2503.8–2895.6)
2025	2604.2 (2412.3–2811.3)	2499.8 (2315.6–2698.6)	2584.1 (2393.6–2789.6)	2622.6 (2429.4–2831.3)
2026	2598.9 (2403.8–2809.8)	2489.8 (2302.9–2691.8)	2575.9 (2382.5–2784.9)	2619.2 (2422.6–2831.7)
2027	2591.3 (2388.2–2811.6)	2477.6 (2283.5–2688.2)	2565.4 (2364.4–2783.5)	2613.4 (2408.6–2835.6)
2028	2570.5 (2366.0–2792.6)	2452.8 (2257.7–2664.8)	2541.9 (2339.7–2761.5)	2594.2 (2387.9–2818.3)
2029	2534.3 (2327.2–2759.9)	2413.6 (2216.3–2628.4)	2503.3 (2298.6–2726.1)	2559.5 (2350.3–2787.4)

2030	2494.0 (2286.3–2720.7)	2370.5 (2173.0–2585.9)	2460.7 (2255.7–2684.3)	2520.6 (2310.7–2749.7)
2031	2461.9 (2256.3–2686.4)	2335.4 (2140.2–2548.3)	2426.2 (2223.5–2647.4)	2490.0 (2281.9–2717.0)
2032	2439.6 (2236.1–2661.6)	2309.6 (2116.9–2519.7)	2401.4 (2201.1–2619.9)	2469.1 (2263.1–2693.8)
2033	2444.2 (2239.6–2667.5)	2309.4 (2116.0–2520.4)	2403.2 (2202.0–2622.8)	2475.5 (2268.2–2701.7)
2034	2406.1 (2204.8–2625.7)	2268.9 (2079.1–2476.0)	2363.0 (2165.4–2578.7)	2438.6 (2234.6–2661.2)
2035	2368.1 (2170.2–2584.1)	2228.6 (2042.3–2431.9)	2323.0 (2128.9–2534.9)	2401.8 (2201.1–2620.9)
2036	2320.1 (2126.0–2532.1)	2179.2 (1996.8–2378.2)	2273.4 (2083.1–2481.1)	2354.8 (2157.8–2569.9)
2037	2295.6 (2100.2–2509.1)	2151.8 (1968.7–2352.0)	2246.7 (2055.5–2455.8)	2331.5 (2133.1–2548.4)
2038	2260.4 (2064.5–2474.8)	2114.7 (1931.4–2315.3)	2209.8 (2018.3–2419.4)	2297.4 (2098.3–2515.4)
2039	2246.6 (2049.4–2462.8)	2097.6 (1913.5–2299.5)	2193.8 (2001.2–2405.0)	2285.1 (2084.4–2505.0)
2040	2200.5 (2006.1–2413.7)	2050.5 (1869.4–2249.1)	2146.3 (1956.7–2354.2)	2239.7 (2041.9–2456.7)

	Women (3–B)			
	20–49			
Year	Reference	Scenario 1	Scenario 2	Scenario 3
2017	97.7 (95.0–100.4)	101.0 (98.3–103.8)	101.0 (98.3–103.8)	101.0 (98.3–103.8)
2018	93.7 (89.9–97.7)	97.4 (93.4–101.5)	98.2 (94.2–102.4)	98.5 (94.5–102.7)
2019	91.0 (87.2–95.1)	95.1 (91.1–99.3)	96.7 (92.6–101.0)	97.3 (93.2–101.7)

2020	87.8 (83.9–91.9)	92.2 (88.1–96.5)	94.6 (90.4–99.0)	95.4 (91.2–99.9)
2021	83.6 (79.9–87.6)	88.3 (84.3–92.4)	91.4 (87.2–95.7)	92.5 (88.3–96.8)
2022	80.1 (76.5–83.9)	85.0 (81.2–89.0)	88.7 (84.7–92.9)	90.1 (86.0–94.3)
2023	77.4 (73.9–81.0)	82.5 (78.8–86.4)	86.9 (82.9–91.0)	88.5 (84.5–92.6)
2024	74.9 (71.6–78.5)	79.5 (75.9–83.2)	85.3 (81.4–89.3)	87.1 (83.2–91.2)
2025	72.7 (69.3–76.2)	76.6 (73.1–80.3)	83.8 (80.0–87.9)	85.9 (81.9–90.0)
2026	70.3 (67.0–73.7)	73.7 (70.3–77.3)	82.2 (78.4–86.2)	84.5 (80.6–88.6)
2027	67.6 (64.5–70.9)	70.6 (67.3–74.0)	80.2 (76.5–84.0)	82.6 (78.8–86.6)
2028	64.8 (61.8–68.0)	67.3 (64.2–70.6)	77.9 (74.3–81.7)	80.6 (76.8–84.5)
2029	62.2 (59.3–65.3)	64.3 (61.3–67.4)	75.9 (72.3–79.6)	78.7 (75.0–82.5)
2030	60.0 (57.2–63.0)	61.7 (58.8–64.7)	74.2 (70.7–77.8)	77.1 (73.6–80.9)
2031	58.1 (55.4–61.0)	59.4 (56.6–62.3)	72.8 (69.4–76.3)	75.9 (72.4–79.7)
2032	56.3 (53.7–59.1)	57.2 (54.5–60.0)	71.5 (68.1–75.0)	74.8 (71.3–78.5)
2033	54.4 (51.9–57.1)	55.0 (52.4–57.7)	70.0 (66.7–73.5)	73.5 (70.1–77.1)
2034	52.4 (49.9–54.9)	52.7 (50.2–55.3)	68.3 (65.1–71.7)	72.0 (68.6–75.5)
2035	50.3 (47.9–52.8)	50.3 (47.9–52.8)	66.5 (63.4–69.8)	70.3 (67.0–73.8)
2036	48.4 (46.1–50.8)	48.1 (45.8–50.5)	64.9 (61.8–68.1)	68.7 (65.5–72.1)
2037	46.6 (44.4–49.0)	46.2 (44.0–48.5)	63.4 (60.4–66.6)	67.4 (64.2–70.7)
2038	45.1 (43.0–47.4)	44.4 (42.3–46.6)	62.2 (59.2–65.3)	66.3 (63.2–69.6)

2039	43.7 (41.6–45.8)	42.8 (40.7–44.9)	61.0 (58.1–64.0)	65.2 (62.1–68.5)
2040	42.2 (40.2–44.3)	41.1 (39.1–43.1)	59.7 (56.9–62.7)	64.1 (61.0–67.3)

	50–69			
Year	Reference	Scenario 1	Scenario 2	Scenario 3
2017	476.3 (464.6–488.3)	480.2 (468.4–492.3)	480.2 (468.4–492.3)	480.2 (468.4–492.3)
2018	456.2 (448.4–464.0)	456.7 (449.0–464.6)	460.4 (452.6–468.3)	461.7 (453.9–469.6)
2019	455.7 (444.2–467.6)	453.2 (441.7–465.0)	460.5 (448.8–472.5)	463.1 (451.4–475.1)
2020	447.6 (436.6–458.8)	442.0 (431.2–453.1)	452.7 (441.7–464.1)	456.6 (445.4–468.1)
2021	423.7 (411.9–435.7)	415.6 (404.1–427.4)	429.1 (417.2–441.3)	434.0 (421.9–446.3)
2022	407.2 (396.6–418.2)	396.7 (386.4–407.4)	412.9 (402.2–424.0)	418.8 (407.9–430.0)
2023	399.0 (384.9–413.7)	386.1 (372.5–400.3)	405.1 (390.8–419.9)	412.0 (397.5–427.1)
2024	391.1 (377.4–405.3)	377.1 (363.9–390.9)	397.5 (383.5–412.0)	405.4 (391.2–420.2)
2025	379.8 (366.6–393.5)	365.0 (352.3–378.1)	386.5 (373.1–400.4)	395.3 (381.6–409.6)
2026	380.6 (366.6–395.1)	364.5 (351.1–378.4)	387.7 (373.5–402.5)	397.7 (383.1–412.9)
2027	371.6 (357.9–385.9)	354.7 (341.6–368.3)	379.0 (365.0–393.6)	389.9 (375.5–404.9)
2028	352.0 (338.7–365.8)	334.8 (322.2–347.9)	359.5 (345.9–373.5)	370.8 (356.8–385.3)
2029	349.5 (336.3–363.3)	331.3 (318.7–344.4)	357.4 (343.8–371.5)	369.7 (355.6–384.3)

2030	336.8 (323.2–350.9)	318.1 (305.3–331.4)	344.7 (330.8–359.1)	357.6 (343.2–372.6)
2031	321.2 (308.4–334.6)	302.4 (290.3–315.0)	329.2 (316.0–342.9)	342.5 (328.8–356.7)
2032	321.1 (308.2–334.5)	301.2 (289.1–313.8)	329.4 (316.2–343.2)	343.7 (329.9–358.1)
2033	312.7 (300.1–325.8)	292.3 (280.6–304.6)	321.2 (308.3–334.7)	336.0 (322.5–350.1)
2034	303.9 (291.5–316.9)	283.2 (271.6–295.3)	312.6 (299.8–325.9)	328.0 (314.6–341.9)
2035	300.3 (288.1–313.1)	278.9 (267.5–290.8)	309.2 (296.6–322.4)	325.4 (312.1–339.2)
2036	292.2 (280.2–304.7)	270.4 (259.3–282.0)	301.2 (288.9–314.1)	317.8 (304.8–331.4)
2037	279.6 (268.0–291.7)	257.9 (247.2–269.0)	288.6 (276.6–301.1)	305.3 (292.7–318.6)
2038	270.4 (259.2–282.2)	248.6 (238.2–259.3)	279.4 (267.8–291.5)	296.5 (284.2–309.4)
2039	266.5 (255.2–278.4)	244.1 (233.7–255.0)	275.7 (264.0–288.0)	293.4 (280.9–306.5)
2040	257.9 (246.9–269.4)	235.4 (225.4–245.9)	267.1 (255.7–279.0)	285.0 (272.9–297.7)

	≥70			
Year	Reference	Scenario 1	Scenario 2	Scenario 3
2017	986.5 (963.0–1010.6)	987.9 (964.4–1012.1)	987.9 (964.4–1012.1)	987.9 (964.4–1012.1)
2018	968.9 (944.4–994.1)	966.5 (942.1–991.6)	969.8 (945.3–995.0)	971.0 (946.4–996.2)
2019	940.9 (916.6–965.8)	934.9 (910.8–959.7)	941.3 (917.0–966.2)	943.6 (919.2–968.6)
2020	926.3 (902.0–951.2)	916.9 (892.8–941.5)	926.3 (902.0–951.2)	929.6 (905.2–954.6)

2021	905.0 (876.2–934.7)	892.3 (863.9–921.6)	904.5 (875.7–934.2)	908.9 (880.0–938.7)
2022	870.4 (837.9–904.1)	854.8 (823.0–887.9)	869.5 (837.1–903.2)	874.7 (842.1–908.6)
2023	868.4 (835.2–902.9)	849.6 (817.2–883.3)	867.1 (834.0–901.5)	873.4 (840.0–908.0)
2024	845.9 (813.5–879.6)	825.4 (793.8–858.3)	844.2 (811.9–877.9)	851.4 (818.8–885.3)
2025	809.5 (778.2–842.1)	787.8 (757.2–819.5)	807.5 (776.2–840.0)	815.3 (783.7–848.2)
2026	799.4 (767.5–832.7)	775.9 (744.8–808.2)	797.1 (765.2–830.3)	805.7 (773.5–839.3)
2027	769.9 (738.3–803.0)	745.3 (714.6–777.2)	767.3 (735.8–800.2)	776.6 (744.6–809.9)
2028	745.5 (715.0–777.3)	719.7 (690.3–750.3)	742.6 (712.2–774.2)	752.4 (721.7–784.5)
2029	739.9 (709.5–771.6)	712.4 (683.1–742.9)	736.6 (706.4–768.2)	747.3 (716.6–779.4)
2030	726.8 (696.2–758.7)	697.9 (668.5–728.5)	723.2 (692.8–755.0)	734.6 (703.7–766.9)
2031	709.8 (680.2–740.6)	679.7 (651.4–709.3)	706.0 (676.6–736.6)	717.9 (688.1–749.1)
2032	693.5 (664.8–723.5)	662.4 (634.9–691.1)	689.5 (660.9–719.3)	702.0 (672.9–732.4)
2033	679.5 (650.9–709.4)	647.3 (620.0–675.7)	675.2 (646.8–704.9)	688.3 (659.3–718.6)
2034	653.9 (624.3–684.8)	621.2 (593.1–650.6)	649.4 (620.0–680.2)	662.8 (632.8–694.2)
2035	637.0 (608.1–667.3)	603.6 (576.2–632.2)	632.4 (603.7–662.4)	646.2 (616.9–676.9)
2036	630.5 (601.9–660.5)	595.8 (568.8–624.2)	625.6 (597.2–655.4)	640.1 (611.0–670.5)
2037	606.4 (578.4–635.7)	571.5 (545.1–599.1)	601.4 (573.6–630.4)	616.0 (587.6–645.7)
2038	593.3 (565.1–622.9)	557.7 (531.1–585.5)	588.1 (560.1–617.5)	603.1 (574.4–633.3)
2039	583.5 (555.1–613.5)	547.0 (520.3–575.1)	578.1 (549.9–607.8)	593.6 (564.7–624.1)

2040	561.3 (534.0–590.1)	524.8 (499.2–551.7)	555.9 (528.8–584.4)	571.5 (543.6–600.7)

	Both sexes combined (3–C)			
	20–49			
Year	Reference	Scenario 1	Scenario 2	Scenario 3
2017	100.2 (98.3–102.1)	104.0 (102.0–106.0)	104.0 (102.0–106.0)	104.0 (102.0–106.0)
2018	95.2 (91.4–99.2)	97.5 (93.6–101.6)	100.0 (96.0–104.2)	100.9 (96.9–105.1)
2019	92.1 (88.3–96.1)	93.0 (89.1–97.0)	97.8 (93.8–102.0)	99.6 (95.5–103.9)
2020	89.5 (85.7–93.5)	89.1 (85.4–93.1)	96.2 (92.1–100.4)	98.8 (94.6–103.1)
2021	85.9 (82.2–89.7)	84.3 (80.7–88.1)	93.3 (89.3–97.5)	96.7 (92.5–101.0)
2022	85.0 (81.3–88.9)	82.3 (78.7–86.1)	93.4 (89.3–97.7)	97.7 (93.4–102.2)
2023	78.2 (74.7–81.7)	74.6 (71.4–78.0)	86.9 (83.1–90.8)	91.6 (87.6–95.8)
2024	79.8 (75.9–83.8)	75.8 (72.1–79.6)	89.6 (85.3–94.2)	95.4 (90.9–100.2)
2025	74.6 (71.0–78.4)	70.5 (67.1–74.1)	84.8 (80.7–89.1)	91.1 (86.7–95.7)
2026	72.1 (68.6–75.8)	67.8 (64.5–71.2)	82.9 (78.9–87.1)	89.8 (85.5–94.4)
2027	71.3 (67.4–75.4)	66.7 (63.0–70.5)	82.9 (78.3–87.7)	90.6 (85.7–95.9)
2028	67.6 (63.9–71.5)	62.9 (59.4–66.5)	79.4 (75.1–84.1)	87.6 (82.8–92.7)
2029	64.2 (60.6–67.9)	59.4 (56.1–62.8)	76.3 (72.1–80.7)	84.9 (80.2–89.9)

2030	64.2 (60.6–67.9)	59.1 (55.8–62.5)	77.2 (72.9–81.7)	86.7 (81.9–91.7)
2031	60.2 (56.7–63.8)	55.1 (52.0–58.4)	73.2 (69.0–77.6)	82.9 (78.2–87.9)
2032	60.5 (57.0–64.1)	55.1 (51.9–58.4)	74.3 (70.1–78.8)	85.0 (80.1–90.1)
2033	56.5 (53.0–60.3)	51.2 (48.0–54.7)	70.3 (65.8–75.0)	81.1 (76.0–86.5)
2034	55.1 (51.6–58.9)	49.7 (46.5–53.1)	69.3 (64.9–74.1)	80.7 (75.5–86.2)
2035	53.1 (49.7–56.8)	47.6 (44.6–50.9)	67.5 (63.2–72.2)	79.3 (74.2–84.8)
2036	50.5 (47.2–54.0)	45.0 (42.1–48.2)	64.9 (60.7–69.4)	76.9 (71.9–82.3)
2037	49.8 (46.5–53.3)	44.2 (41.3–47.3)	64.8 (60.5–69.3)	77.4 (72.4–82.9)
2038	49.2 (46.0–52.6)	43.4 (40.6–46.5)	64.7 (60.5–69.2)	78.0 (72.9–83.5)
2039	45.5 (42.5–48.7)	40.0 (37.4–42.8)	60.5 (56.6–64.8)	73.7 (68.9–78.8)
2040	45.5 (42.5–48.8)	39.8 (37.1–42.6)	61.2 (57.2–65.6)	75.2 (70.2–80.5)

	50–69			
Year	Reference	Scenario 1	Scenario 2	Scenario 3
2017	957.6 (936.6–979.0)	970.5 (949.3–992.3)	970.5 (949.3–992.3)	970.5 (949.3–992.3)
2018	909.9 (877.4–943.7)	905.3 (872.9–938.8)	922.4 (889.4–956.6)	928.5 (895.3–962.9)
2019	892.4 (861.2–924.8)	871.5 (841.0–903.1)	904.8 (873.1–937.5)	916.8 (884.7–950.0)
2020	843.8 (814.5–874.0)	808.8 (780.7–837.8)	855.5 (825.9–886.2)	872.7 (842.4–904.0)

2021	796.3 (768.6–825.1)	749.2 (723.1–776.3)	807.5 (779.4–836.7)	829.2 (800.2–859.1)
2022	827.8 (790.7–866.7)	764.5 (730.2–800.4)	839.6 (801.9–879.0)	867.8 (828.9–908.5)
2023	807.4 (769.0–847.7)	731.9 (697.1–768.4)	818.9 (780.0–859.8)	852.1 (811.6–894.6)
2024	783.1 (743.5–824.7)	706.4 (670.7–744.0)	794.4 (754.2–836.6)	832.0 (789.9–876.3)
2025	767.2 (728.8–807.6)	688.7 (654.2–725.0)	778.3 (739.4–819.3)	820.6 (779.5–863.8)
2026	718.1 (682.2–755.9)	641.6 (609.5–675.4)	728.7 (692.2–767.0)	773.3 (734.7–814.1)
2027	718.3 (679.5–759.3)	638.6 (604.1–675.1)	728.9 (689.5–770.5)	778.7 (736.6–823.2)
2028	704.0 (666.1–744.0)	622.9 (589.4–658.3)	714.5 (676.0–755.1)	768.4 (727.0–812.1)
2029	670.0 (632.2–710.1)	590.0 (556.7–625.3)	680.1 (641.7–720.8)	736.3 (694.7–780.4)
2030	678.4 (639.6–719.5)	594.5 (560.5–630.5)	688.6 (649.3–730.4)	750.5 (707.6–795.9)
2031	655.9 (618.2–696.0)	572.0 (539.1–607.0)	666.0 (627.6–706.7)	730.6 (688.5–775.2)
2032	639.5 (602.7–678.6)	555.0 (523.1–588.9)	649.4 (612.0–689.0)	717.1 (675.8–760.9)
2033	625.6 (589.6–663.9)	540.4 (509.2–573.4)	635.3 (598.7–674.2)	706.2 (665.5–749.5)
2034	582.8 (548.9–618.8)	501.0 (471.8–531.9)	592.0 (557.5–628.5)	662.4 (623.9–703.3)
2035	582.3 (547.2–619.7)	498.1 (468.1–530.1)	591.5 (555.9–629.5)	666.3 (626.1–709.1)
2036	582.2 (547.1–619.4)	495.6 (465.7–527.3)	591.4 (555.8–629.3)	670.6 (630.2–713.5)
2037	564.2 (529.1–601.6)	478.0 (448.2–509.6)	573.2 (537.6–611.2)	654.3 (613.6–697.6)
2038	559.9 (525.1–596.9)	472.0 (442.7–503.2)	568.9 (533.6–606.5)	653.7 (613.1–696.9)
2039	526.4 (493.5–561.5)	441.7 (414.1–471.1)	535.0 (501.6–570.6)	618.7 (580.1–660.0)

2040	509.4 (477.1–543.8)	425.3 (398.3–454.1)	517.7 (484.9–552.8)	602.8 (564.5–643.5)

	≥70			
Year	Reference	Scenario 1	Scenario 2	Scenario 3
2017	1917.9 (1868.2–1969.1)	1923.3 (1873.4–1974.6)	1923.3 (1873.4–1974.6)	1923.3 (1873.4–1974.6)
2018	1871.2 (1814.5–1929.6)	1856.8 (1800.6–1914.8)	1873.2 (1816.4–1931.7)	1879.0 (1822.1–1937.7)
2019	1805.7 (1740.5–1873.3)	1773.1 (1709.1–1839.6)	1804.6 (1739.4–1872.2)	1815.8 (1750.2–1883.8)
2020	1764.3 (1698.3–1832.8)	1714.4 (1650.3–1781.0)	1760.2 (1694.4–1828.6)	1776.6 (1710.2–1845.7)
2021	1708.5 (1637.2–1783.1)	1642.9 (1574.3–1714.6)	1701.7 (1630.6–1775.9)	1722.9 (1650.9–1798.0)
2022	1662.4 (1590.5–1737.6)	1581.9 (1513.4–1653.4)	1652.9 (1581.4–1727.6)	1678.7 (1606.1–1754.6)
2023	1668.8 (1596.0–1745.0)	1571.4 (1502.9–1643.1)	1656.5 (1584.2–1732.0)	1687.6 (1613.9–1764.6)
2024	1638.2 (1566.3–1713.4)	1535.1 (1467.8–1605.6)	1623.2 (1552.0–1697.8)	1658.9 (1586.1–1735.0)
2025	1589.6 (1516.3–1666.5)	1482.5 (1414.2–1554.2)	1572.5 (1500.0–1648.5)	1612.0 (1537.6–1689.9)
2026	1580.6 (1506.4–1658.4)	1467.0 (1398.2–1539.3)	1560.9 (1487.6–1637.7)	1605.0 (1529.7–1684.1)
2027	1560.8 (1485.8–1639.5)	1441.7 (1372.5–1514.4)	1538.6 (1464.8–1616.3)	1587.1 (1510.9–1667.2)
2028	1506.3 (1434.0–1582.3)	1384.8 (1318.3–1454.6)	1482.5 (1411.3–1557.2)	1533.9 (1460.2–1611.3)
2029	1480.5 (1409.3–1555.4)	1354.5 (1289.4–1423.0)	1454.6 (1384.6–1528.1)	1509.7 (1437.1–1586.0)
2030	1445.4 (1375.8–1518.5)	1316.1 (1252.7–1382.6)	1417.7 (1349.4–1489.4)	1476.0 (1404.9–1550.6)

2031	1393.6 (1326.2–1464.3)	1262.8 (1201.8–1326.9)	1364.5 (1298.5–1433.7)	1425.0 (1356.2–1497.4)
2032	1381.2 (1314.5–1451.2)	1245.6 (1185.5–1308.7)	1350.0 (1284.9–1418.5)	1414.3 (1346.1–1486.0)
2033	1382.8 (1315.2–1453.9)	1241.1 (1180.4–1304.9)	1349.3 (1283.4–1418.7)	1418.0 (1348.6–1490.9)
2034	1347.6 (1281.3–1417.3)	1203.7 (1144.5–1265.9)	1312.7 (1248.2–1380.6)	1383.8 (1315.8–1455.4)
2035	1323.5 (1256.1–1394.5)	1176.5 (1116.6–1239.6)	1287.1 (1221.5–1356.1)	1360.9 (1291.6–1434.0)
2036	1303.6 (1237.3–1373.4)	1153.3 (1094.6–1215.0)	1265.6 (1201.2–1333.3)	1342.4 (1274.1–1414.2)
2037	1255.9 (1191.9–1323.3)	1105.7 (1049.4–1165.1)	1217.2 (1155.1–1282.5)	1295.0 (1229.0–1364.6)
2038	1222.6 (1160.2–1288.3)	1071.2 (1016.6–1128.8)	1182.8 (1122.5–1246.4)	1262.4 (1198.0–1330.3)
2039	1213.3 (1150.5–1279.5)	1058.0 (1003.2–1115.8)	1171.9 (1111.2–1235.8)	1254.6 (1189.6–1323.1)
2040	1182.3 (1120.8–1247.1)	1026.1 (972.7–1082.3)	1140.0 (1080.7–1202.5)	1224.3 (1160.6–1291.4)

	All age (3–D)			
	Men			
Year	Reference	Scenario 1	Scenario 2	Scenario 3
2017	925.9 (898.9–953.6)	934.0 (906.8–962.0)	934.0 (906.8–962.0)	934.0 (906.8–962.0)
2018	925.4 (892.5–959.4)	924.5 (891.7–958.5)	934.1 (900.9–968.5)	937.5 (904.2–972.0)
2019	923.4 (888.0–960.2)	913.1 (878.1–949.5)	932.1 (896.4–969.3)	939.0 (903.0–976.4)
2020	920.3 (883.8–958.2)	900.8 (865.1–937.9)	929.1 (892.3–967.4)	939.3 (902.1–978.0)

2021	916.3 (879.4–954.7)	887.7 (852.0–924.9)	925.1 (887.9–963.9)	938.7 (900.9–978.1)
2022	911.7 (874.7–950.3)	874.3 (838.8–911.3)	920.6 (883.2–959.5)	937.5 (899.5–977.1)
2023	906.8 (869.8–945.2)	860.7 (825.6–897.2)	915.6 (878.3–954.5)	935.8 (897.7–975.6)
2024	901.5 (864.7–939.9)	854.4 (819.5–890.7)	910.4 (873.2–949.1)	933.9 (895.8–973.6)
2025	896.1 (859.5–934.2)	847.9 (813.3–884.0)	905.0 (868.0–943.5)	931.7 (893.7–971.4)
2026	890.6 (854.2–928.5)	841.4 (807.0–877.2)	899.5 (862.7–937.8)	929.4 (891.4–969.0)
2027	885.0 (848.8–922.7)	834.8 (800.7–870.4)	893.9 (857.3–931.9)	927.0 (889.1–966.5)
2028	879.3 (843.4–916.8)	828.2 (794.4–863.5)	888.2 (851.9–926.1)	924.5 (886.7–963.9)
2029	873.7 (837.9–910.9)	821.7 (788.1–856.7)	882.6 (846.5–920.2)	922.0 (884.3–961.3)
2030	868.0 (832.5–905.0)	815.1 (781.8–849.8)	876.9 (841.1–914.3)	919.4 (881.8–958.6)
2031	862.4 (827.1–899.1)	808.5 (775.5–843.0)	871.3 (835.7–908.4)	916.8 (879.4–955.9)
2032	856.7 (821.7–893.2)	802.0 (769.3–836.2)	865.7 (830.3–902.5)	914.2 (876.9–953.2)
2033	851.1 (816.3–887.4)	795.6 (763.1–829.5)	860.1 (824.9–896.7)	911.7 (874.4–950.5)
2034	845.6 (811.0–881.6)	789.2 (756.9–822.8)	854.5 (819.6–890.9)	909.0 (871.9–947.8)
2035	840.0 (805.7–875.8)	782.8 (750.8–816.1)	848.9 (814.2–885.1)	906.4 (869.4–945.1)
2036	834.5 (800.4–870.1)	776.5 (744.7–809.6)	843.4 (809.0–879.4)	903.9 (866.9–942.4)
2037	829.0 (795.1–864.4)	770.2 (738.7–803.0)	838.0 (803.7–873.7)	901.3 (864.4–939.7)
2038	823.6 (789.9–858.7)	764.0 (732.7–796.5)	832.5 (798.5–868.0)	898.7 (861.9–937.0)
2039	818.2 (784.7–853.1)	757.8 (726.8–790.1)	827.1 (793.3–862.4)	896.1 (859.5–934.3)

2040	812.8 (779.6–847.5)	751.6 (720.9–783.7)	821.7 (788.1–856.7)	893.5 (857.0–931.6)

	Women			
Year	Reference	Scenario 1	Scenario 2	Scenario 3
2017	394.2 (389.2–399.3)	397.2 (392.2–402.4)	397.2 (392.2–402.4)	397.2 (392.2–402.4)
2018	378.4 (373.6–383.2)	379.9 (375.1–384.7)	382.0 (377.2–386.8)	382.7 (377.9–387.6)
2019	368.8 (363.1–374.6)	369.0 (363.3–374.8)	373.0 (367.3–378.9)	374.5 (368.7–380.4)
2020	370.9 (365.5–376.4)	369.7 (364.4–375.2)	375.9 (370.4–381.4)	378.1 (372.6–383.6)
2021	354.3 (349.1–359.6)	351.9 (346.8–357.2)	359.8 (354.5–365.1)	362.6 (357.2–368.0)
2022	349.8 (344.6–355.0)	346.2 (341.1–351.3)	355.8 (350.6–361.1)	359.3 (354.0–364.6)
2023	359.3 (352.7–366.1)	354.3 (347.8–361.0)	366.2 (359.5–373.1)	370.5 (363.7–377.4)
2024	347.4 (341.2–353.7)	341.6 (335.5–347.8)	354.7 (348.4–361.2)	359.5 (353.1–366.1)
2025	337.8 (331.1–344.7)	331.2 (324.7–337.9)	345.6 (338.7–352.6)	351.0 (344.0–358.1)
2026	339.3 (332.8–346.0)	331.8 (325.4–338.3)	347.8 (341.1–354.6)	353.9 (347.1–360.9)
2027	325.7 (319.4–332.1)	317.6 (311.4–323.8)	334.4 (328.0–341.0)	341.0 (334.4–347.7)
2028	319.5 (313.3–325.8)	310.6 (304.6–316.8)	328.6 (322.3–335.1)	335.7 (329.2–342.4)
2029	327.3 (320.2–334.6)	317.4 (310.4–324.5)	337.4 (330.0–344.9)	345.3 (337.8–353.0)
2030	318.7 (311.9–325.7)	308.2 (301.6–314.9)	329.1 (322.1–336.4)	337.5 (330.3–344.9)

2031	309.5 (302.4–316.7)	298.4 (291.6–305.3)	320.2 (312.9–327.6)	329.0 (321.5–336.6)
2032	310.4 (303.4–317.5)	298.5 (291.7–305.3)	321.7 (314.5–329.1)	331.2 (323.8–338.9)
2033	299.3 (292.6–306.2)	287.0 (280.5–293.6)	310.8 (303.8–317.9)	320.6 (313.4–328.0)
2034	292.1 (285.5–298.8)	279.3 (273.0–285.7)	303.9 (297.0–310.9)	314.1 (307.0–321.3)
2035	298.2 (291.0–305.6)	284.3 (277.4–291.4)	310.8 (303.3–318.5)	321.9 (314.1–329.9)
2036	292.2 (285.1–299.4)	277.8 (271.1–284.7)	305.1 (297.8–312.7)	316.6 (309.0–324.4)
2037	283.5 (276.5–290.8)	268.8 (262.1–275.7)	296.7 (289.3–304.2)	308.4 (300.7–316.3)
2038	283.9 (276.9–291.1)	268.4 (261.8–275.2)	297.6 (290.2–305.2)	310.0 (302.3–317.9)
2039	274.9 (268.1–281.9)	259.2 (252.8–265.8)	288.7 (281.6–296.1)	301.3 (293.9–309.0)
2040	267.3 (260.6–274.1)	251.3 (245.0–257.7)	281.2 (274.2–288.4)	294.1 (286.7–301.5)

	Both sexes combined			
Year	Reference	Scenario 1	Scenario 2	Scenario 3
2017	656.5 (643.9–669.4)	660.5 (647.8–673.5)	660.5 (647.8–673.5)	660.5 (647.8–673.5)
2018	637.6 (627.1–648.2)	638.0 (627.6–648.7)	642.0 (631.5–652.7)	643.5 (632.9–654.2)
2019	629.9 (619.8–640.3)	627.0 (616.9–637.3)	634.9 (624.6–645.3)	637.7 (627.4–648.2)
2020	636.0 (625.6–646.6)	629.6 (619.3–640.1)	641.6 (631.1–652.3)	645.8 (635.3–656.6)
2021	620.7 (608.0–633.7)	611.2 (598.7–624.0)	626.7 (613.9–639.8)	632.3 (619.3–645.5)

2022	617.6 (605.2–630.3)	604.8 (592.7–617.3)	624.1 (611.5–636.9)	631.0 (618.3–644.0)
2023	617.5 (605.2–630.1)	601.5 (589.5–613.8)	624.5 (612.1–637.3)	632.9 (620.2–645.8)
2024	603.8 (591.6–616.3)	587.3 (575.4–599.4)	611.2 (598.9–623.8)	620.7 (608.2–633.5)
2025	583.6 (569.4–598.1)	566.7 (552.9–580.8)	591.2 (576.9–605.9)	601.8 (587.2–616.7)
2026	583.1 (570.8–595.7)	565.3 (553.4–577.5)	591.2 (578.8–604.0)	603.1 (590.4–616.1)
2027	573.0 (560.9–585.4)	554.7 (543.0–566.7)	581.5 (569.2–594.1)	594.5 (581.9–607.3)
2028	561.1 (549.2–573.3)	542.3 (530.8–554.1)	569.9 (557.8–582.3)	584.0 (571.6–596.6)
2029	569.0 (555.0–583.3)	549.1 (535.5–562.9)	578.4 (564.2–593.0)	593.9 (579.3–608.9)
2030	558.4 (544.5–572.8)	538.0 (524.6–551.9)	568.2 (554.0–582.8)	584.7 (570.1–599.7)
2031	547.6 (534.1–561.5)	526.8 (513.8–540.1)	557.7 (543.9–571.8)	575.2 (561.0–589.7)
2032	548.9 (535.0–563.1)	527.2 (513.9–540.8)	559.4 (545.3–573.9)	578.3 (563.7–593.2)
2033	545.3 (530.9–560.1)	522.9 (509.2–537.1)	556.3 (541.6–571.3)	576.3 (561.1–591.9)
2034	535.0 (521.1–549.3)	512.3 (499.0–526.0)	546.2 (532.0–560.8)	567.1 (552.4–582.3)
2035	533.8 (519.9–548.1)	510.3 (497.0–524.0)	545.4 (531.2–560.1)	567.6 (552.7–582.8)
2036	527.0 (513.1–541.2)	503.0 (489.7–516.6)	538.9 (524.7–553.5)	562.0 (547.2–577.2)
2037	507.4 (493.2–522.0)	483.6 (470.0–497.5)	519.3 (504.8–534.3)	542.8 (527.6–558.4)
2038	505.6 (491.8–519.8)	481.1 (467.9–494.6)	517.9 (503.8–532.5)	542.5 (527.7–557.8)
2039	501.5 (487.8–515.5)	476.4 (463.4–489.8)	514.2 (500.1–528.6)	539.8 (525.0–554.9)

2040	491.2 (477.7–505.1)	465.9 (453.1–479.1)	504.1 (490.2–518.4)	530.3 (515.7–545.4)

1: best scenario; 2: moderate scenario; 3: worse scenario
